# Discovery of potent low-toxicity antimicrobial peptides through diffusion modeling

**DOI:** 10.1038/s41467-026-75030-8

**Published:** 2026-07-07

**Authors:** Konstantinos Markakis, Shanghyeon Kim, Cheng-En Tan, Ilias Tagkopoulos

**Affiliations:** 1 Department of Computer Science, University of California, Davis, Davis, CA USA; 2 Genome Center, University of California, Davis, CA USA; 3 USDA/NSF AI Institute for Next Generation Food Systems (AIFS), University of California, Davis, CA USA

**Keywords:** Computational platforms and environments, Virtual screening

## Abstract

The rapid emergence of multidrug-resistant bacteria has created an urgent need for improved antimicrobial discovery and screening platforms. Here, we present ARCADIAMP, a generative and virtual screening platform that couples an iterative-learning discrete denoising diffusion probabilistic model with a two-stage Evolutionary Scale Modeling 2 (ESM2)-based antibacterial activity classifier to generate, classify, and prioritize potent AMPs with high activity, low toxicity, and favorable serum stability. Eight of the ten experimentally screened peptide candidates showed antimicrobial activity (MIC ≤ 32 μg/mL), while one generated candidate, Arcinin, demonstrated strong activity against ESKAPE pathogens (MIC 8–32 μg/mL), low hemolytic activity (LC_50_ > 512 μg/mL for human red blood cells), and strong serum-retained activity (MIC 32 μg/mL in 50% bovine serum for four ESKAPE species). Electron microscopy, membrane depolarization assays, time-kill kinetics, and molecular dynamics simulations showed that Arcinin acts through sub-microsecond insertion and penetration consistent with the behavior of other well-known AMPs. In a bacteria-infected wound murine model, Arcinin achieved a 4-log reduction in bacterial burden, which facilitated subsequent re-epithelialization and wound recovery. By framing antimicrobial discovery as an AI-assisted iterative optimization problem, ARCADIAMP links activity, toxicity, and efficacy and provides a scalable template for discovering therapeutically promising biologics.

## Abstract (web summary)

In this study, Markakis et al. use a generative AI model to design antimicrobial peptides that are both potent and safe. Their lead candidate, Arcinin, kills drug-resistant bacteria, spares human cells, and clears infection in a mouse wound model.

## Introduction

Overuse and misuse of antibiotics have driven the evolution of multidrug-resistant (MDR) bacterial strains^1–3^. The rapid emergence of MDR bacteria poses a formidable global health threat, with projections estimating 5 to 10 million annual deaths by 2050 if left unaddressed^4,5^. As the effectiveness of conventional antibiotics continues to decline^6,7^, antimicrobial peptides (AMPs) have emerged as a promising therapeutic alternative^7–9^. Unlike traditional antibiotics, which typically target specific proteins or nucleic acids, AMPs primarily disrupt bacterial membranes, a mechanism that offers a high fitness barrier to the rapid evolution of resistance observed with traditional protein-targeting, RNA-targeting antibiotics^7–14^. Indeed, colistin and daptomycin remain important last-resort treatments for MDR infections^15^. However, despite decades of interest, no new AMP has been approved for clinical use since their introduction^15–17^. Although hundreds of peptides have shown strong in vitro activity, many candidates have failed in clinical trials, highlighting the major barriers to translation: in vivo toxicity and poor serum stability^15,17–21^.

Recently, machine learning (ML) and other computational approaches have been applied to accelerate AMP discovery^22,23^. By enabling high-throughput in silico evaluation of large sequence libraries, ML-based methods can de-risk development and shorten discovery timelines^22,23^. These approaches include regression models for predicting antimicrobial activity^22,23^, binary classifiers for AMP identification^24–27^, and generative models^16^ such as generative adversarial networks (GANs)^28^, variational autoencoders (VAEs)^16^, and diffusion models^29^ for designing novel sequences. However, most existing approaches optimize antimicrobial activity alone, often yielding potentially potent candidates that are ultimately unsuitable for therapeutic use because of high toxicity or poor pharmacokinetic properties^30–32^.

To address these limitations, we introduce ARCADIAMP, a generative AI platform designed to co-optimize for a high therapeutic index^33,34^, that is, high activity combined with low toxicity. By integrating an iterative-learning discrete denoising diffusion probabilistic (D3PM)^35,36^ generative model, a two-stage Evolutionary Scale Modeling 2 (ESM2)-based^37^ antibacterial activity classifier, and a sequence novelty filter that compares generated candidates with published AMP sequences, ARCADIAMP explores peptide sequence space to identify de novo candidates with clinical potential. Two key computational advances underpin this framework: therapeutic-index-based sample weighting, which connects activity with pharmacokinetic relevance during training, and iterative learning through repeated generate-classify/diversify-learn cycles. Together, these advances led to an 80% experimental validation success rate for antimicrobial activity and culminated in the discovery of Arcinin, a novel peptide with potent activity against ESKAPE pathogens (*Enterococcus faecalis*, *Staphylococcus aureus*, *Klebsiella pneumoniae*, *Acinetobacter baumannii*, *Pseudomonas aeruginosa*, and *Enterobacter species*). These pathogens are designated by the World Health Organization as high-priority targets because of their ability to “escape” the biocidal effects of conventional antibiotics through diverse resistance mechanisms^18^. Notably, although the final “E” in ESKAPE denotes Enterobacter species, we used *Escherichia coli* as the primary Gram-negative representative because it accounts for the most populous subset of experimentally validated antimicrobial activity data entries inside AMP databases. This data-driven prioritization enabled the development of highly accurate classifiers and ultimately led to the discovery of Arcinin, which demonstrated strong activity (MIC 8–32 μg/mL), negligible toxicity, and strong stability, comparable to existing AMPs that have reached phase 3 clinical trials^16,38^.

## Results

### Iterative learning and cascade filtering result in improved, clinical-grade AMP candidates

**Fig. 1:**
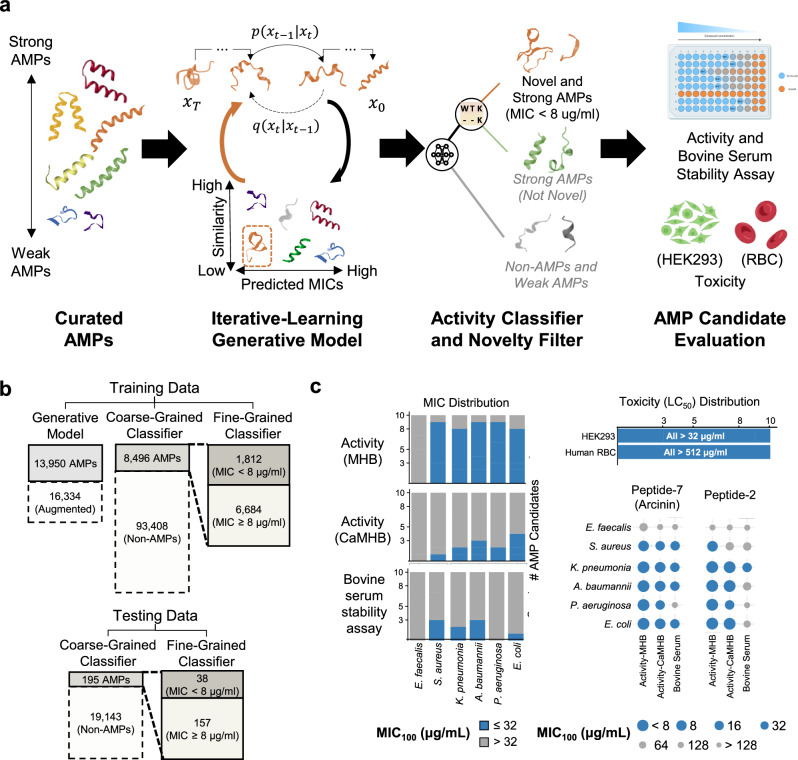
Overview of the AMP screening platform. **a** Core components of the platform, including the iterative-learning D3PM-based generative model, the antibacterial activity classifier, and the sequence novelty filter. Screened AMP sequences were experimentally validated for activity, toxicity, and serum stability. **b** Training datasets used for the generative model and the two-stage classifier. **c** Summary of the in vitro validation results, including antibacterial activity, toxicity, and serum stability profiles of the ten screened candidates. Some elements were created in BioRender. Tan, C. (2026) https://BioRender.com/ybh4kny; Tan, C. (2026) https://BioRender.com/4ltx7x6; Tan, C. (2026) https://BioRender.com/eddp88y.

The AMP screening pipeline consists of a generative model based on a D3PM architecture^36^, followed by two filters (Fig. 1a; Supplementary Figs. 1, 2). The first filter is a two-stage ESM2-based antibacterial activity classifier comprising a coarse-grained model that distinguishes AMPs from non-AMPs and a fine-grained model that identifies strong AMPs (MIC < 8 μg/mL). The second filter is a sequence novelty filter that compares each candidate with known AMPs (Supplementary Figs. 3 and 4), thereby ensuring that selected candidates are both novel and strongly antibacterial (Fig. 1).

We found that the model trained without pretraining generated sequences with significantly stronger predicted antibacterial activity, albeit with lower sequence novelty (Supplementary Fig. 5). To prioritize antibacterial activity, we therefore trained the model from randomly initialized weights rather than fine-tuning a pretrained model. We also compared D3PM with OADM, the alternative framework provided in the original EvoDiff study. Although OADM performed better across the full set of 30,000 generated sequences in terms of predicted antibacterial activity (Supplementary Fig. 5), D3PM outperformed OADM when we focused on the top 100 sequences with the strongest predicted antibacterial activity (Supplementary Fig. 6). We therefore selected D3PM because it performed better in the practical screening scenario of prioritizing the strongest AMP candidates.

**Fig. 2:**
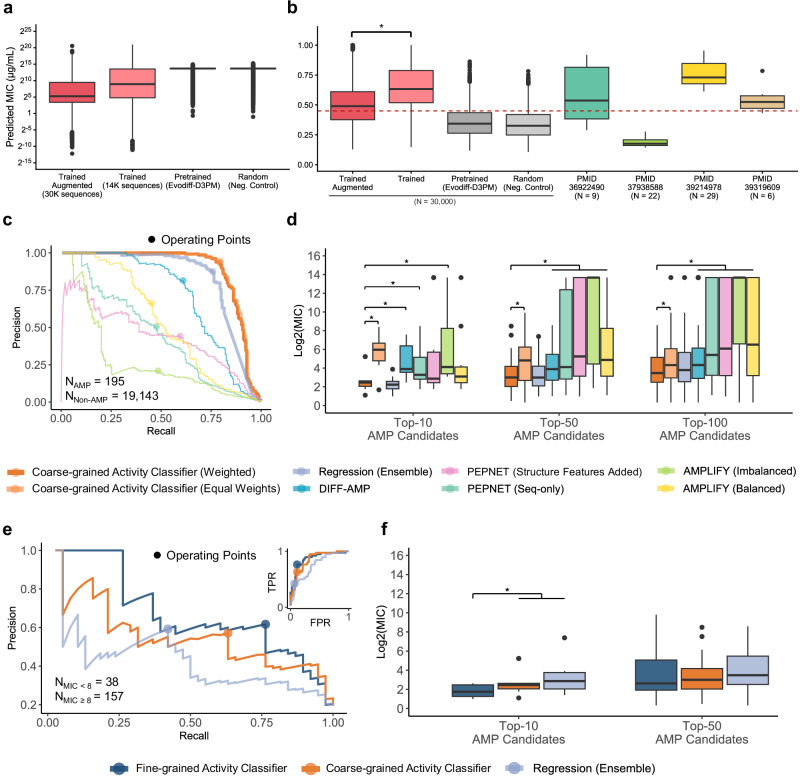
Performance of the iterative-learning generative model and two-stage activity classifier. **a** Predicted MICs of peptides generated by models with augmented training, without augmented training, pretrained D3PM, and random-sequence controls. *N* = 300,000 for the model with augmented training and *N* = 30,000 for the other models. Box plots are shown on a log2 scale. For box plots, the center line indicates the median (Q2), box boundaries indicate the 25th and 75th percentiles (Q1 and Q3), and whiskers extend to the most extreme values within 1.5 × IQR. For log-scaled panels, box-plot statistics were calculated after log transformation. Statistical significance was assessed using a two-sided Wilcoxon rank-sum test. **b** Sequence novelty across models and published studies, calculated as similarity between generated sequences and 27,636 published AMPs. Box plots are defined as in a, without log transformation. Statistical significance was assessed using a two-sided Wilcoxon rank-sum test. **c** Precision-recall curves for AMP versus non-AMP classification using the coarse-grained 8 M activity classifier and alternative methods, including an ensemble regressor constructed from previously published models (recall, 79% vs 76%; precision, 93% vs 88%; F1-score, 86% vs 81%; balanced accuracy, 90% vs 88%). Bootstrapping results (20% test data, 100 iterations) are also shown: recall, 81 ± 6% vs 77 ± 6% (*p* = 3.1 × 10^−6^); precision, 96 ± 4% vs 91 ± 6% (*p* = 8.2 × 10^−12^); F1-score, 88 ± 4% vs 83 ± 4% (*p* = 5.6 × 10^−14^); and balanced accuracy, 90 ± 3% vs 88 ± 3% (*p* = 2.4 × 10^−6^). Statistical significance was assessed using a two-sided t-test. Box plots are defined as in a, without log transformation. **d** Experimentally reported log2MIC values for the top-ranked candidates identified by each model; lower values indicate stronger potency. Box plots are defined as in a. Statistical significance was assessed using a two-sided t-test (the exact p-value of the weighted coarse-grained classifier vs. PEPNET (seq-only), PEPNET (structure features added), AMPLIFY (imbalanced), and AMPLIFY (balanced), is shown as follows: 8.6*10^−5^, 8.7*10^−7^, 5.1*10^−12^, 2.9*10^-5^, for Top-50 candidates, and 2.9*10^−^^10^, 4.0*10^−11^, <2.2*10^−16^, and 3.7*10^−11^ for Top-100 candidates). Box plots are defined as in (**a**). **e** Precision**-**recall and ROC curves for classification of strong AMPs (MIC < 8 μg/mL) by the fine-grained, coarse-grained, and ensemble regression models. **f** Experimentally reported log2MIC values for the top-ranked candidates identified by each model; lower values indicate stronger potency. Box plots are defined as in a. Statistical significance was assessed using a one-sided t-test.

We further found that iterative learning, that is, incorporating high-scoring predictions into later training rounds, improved model performance. The initial model was trained on 13,950 AMP sequences targeting ESKAPE^39^ species from the DBAASP^40^ database, using equal sample weights. We then augmented the training set with another 16,334 generated sequences with predicted MIC < 32 μg/mL by a published MIC predictor by Huang et al.^22^, and applied sample-wise weighting based on each peptide’s estimated therapeutic index (Supplementary Fig. 7). After this augmented training, the generative model produced sequences with highly improved predicted MICs (median MIC 37.8 μg/mL vs 495.3 μg/mL; p-value < 2.2 × 10^−16^; Fig. 2a) and greater sequence novelty (0.49 vs 0.63, *p*-value < 2.2 × 10^−^^16^ for median similarity (normalized BLOSUM62^41^ alignment score), and 0.56 vs 0.69, p-value < 2.2 × 10^−^^16^ for median identity; Fig. 2b and Supplementary Fig. 8). We also compared the model with two published and accessible architectures, including BroadAMP-GPT^42^ (GPT-based) and Pep-Diffusion^43^ (latent diffusion-based). Although our model generated sequences with lower average novelty than Pep-Diffusion, it produced sequences with exceptionally stronger predicted antibacterial activity than all compared models (Supplementary Fig. 9). We additionally evaluated performance across further augmented training iterations. More iterations further improved both predicted antibacterial activity (Supplementary Table 1; Supplementary Fig. 10a) and sequence novelty (Supplementary Fig. 10b, c), showcasing the utility of this approach for efficient generation of strong and novel peptides.

We also applied the sample-wise weighting scheme during training of both the coarse-grained and fine-grained activity classifiers (Supplementary Fig. 11a, b). As a result, the coarse-grained classifier outperformed previous models on the test set, which comprised 195 AMPs and 19,143 non-AMPs (Fig. 2b), including an ensemble MIC regressor built from four published models (F1 score 0.86 vs 0.81, *p*-value < 2.2 × 10^−16^; Fig. 2c; Supplementary Fig. 12; Supplementary Table 2) and screened AMPs with significantly lower MICs than published binary classifiers and the coarse-grained classifier trained with equal weights (22.5–64.7% lower average Log_2_MIC values for top-50 MICs, *p*-value < 0.015; Fig. 2d). The fine-grained classifier further improved upon the coarse-grained classifier (F1-score of 0.68 vs 0.60, *p*-value < 2.2 × 10^−16^ for MIC < 8 μg/mL; Fig. 2e, f; Supplementary Figs. 13 and 14).

### Discovery of Arcinin, a potent AMP with low toxicity and high in-vitro serum stability

**Fig. 3:**
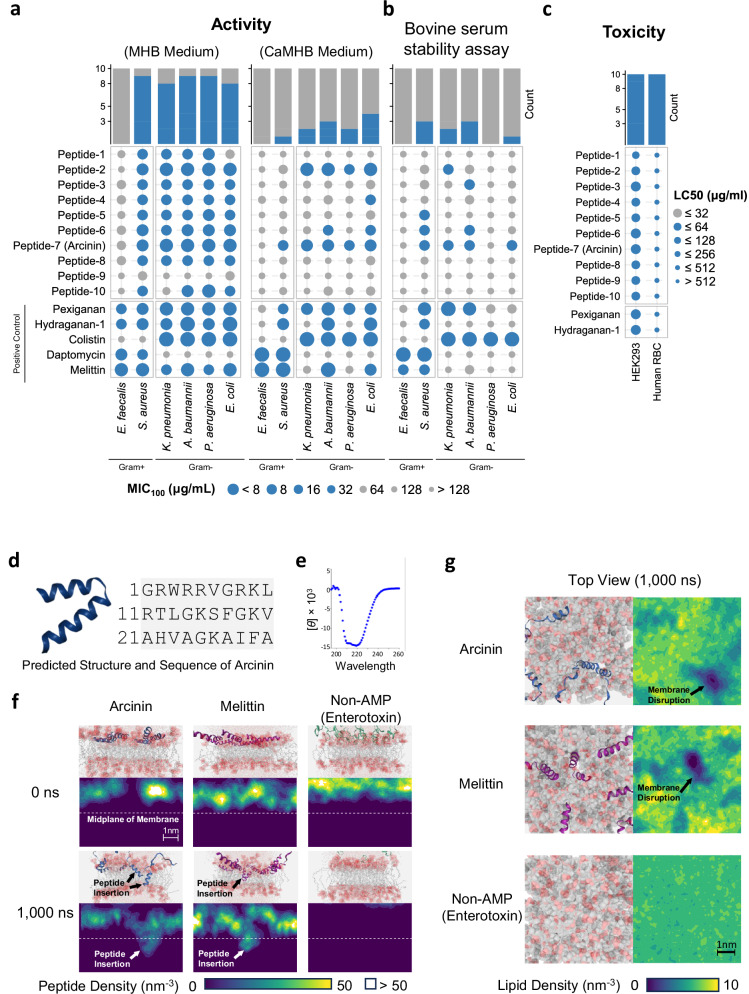
Molecular dynamics and in-vitro experimental results of AMP candidates. **a** MICs of ten AMP candidates and published AMPs against ESKAPE species in MHB and CaMHB media. **b** Serum stability assessment of the same peptides in 50% bovine serum, reported as MIC values. **c** Cytotoxicity profiles (LC_50_) of AMP candidates and published AMPs against HEK293 cells and human red blood cells (RBC). **d** The predicted structure and sequence of Arcinin; **e** CD spectrum of Arcinin, showing two minima near 208 and 222 nm, consistent with an α-helical conformation in a bacterial membrane-mimetic environment. **f** Peptide density in peptide-membrane systems containing Arcinin, melittin, or the non-AMP control (enterotoxin, PDB ID: 7CSS) at 0 and 1000 ns, showing insertion for Arcinin and melittin but not for enterotoxin. **g** Top-to-bottom view of the peptide-membrane system at 1000 ns together with the corresponding DMPC lipid-density plot.

To evaluate the platform, we generated 1,000,000 candidate sequences and applied the screening filters. The top 10,000 candidates ranked by the coarse classifier were retained. Among these, 76 sequences achieved a fine-classifier probability > 0.8 for strong activity and also passed the novelty filter. From these 76, we selected ten peptides for synthesis and testing. Eight of the ten candidates exhibited MIC ≤ 32 μg/mL in standard MHB against four Gram-negative species and S. aureus. Notably, Peptide-2 and Peptide-7 (Arcinin) achieved MICs of 8 μg/mL against all four Gram-negative species in MHB (Table 1), while maintaining MICs ≤ 32 μg/mL in cation-adjusted MHB (CaMHB) (Figs. 1c and 3a; Supplementary Table 3).

**Table 1:** Antibacterial activity (MICs) and toxicity (LC_50_) of AMP candidates compared with published AMPs

Peptide	(Sequence)	Activity (MIC) (MHB Medium) (μg/mL)						Toxicity (LC_50_) (μg/mL)	
		Gram (+)		Gram (-)				HEK293	Human Red Blood Cell
		*E. faecalis* ATCC 29212	*S. aureus* ATCC 25923	*K. pneumoniae* ATCC 700603	*A. baumannii* ATCC 19606	*P. aeruginosa* ATCC 27853	*E. coli* ATCC 25922		
Peptide-1	GWWSKFRKKLKRNAWKIGNKIRPRGIKRGL	128	**32**	**32**	**32**	**16**	64	160.79	>512
Peptide-2	KKISSLKKKIGHFARKIKGPLGRIAIQSLV	>128	**16**	**8**	**8**	**8**	**8**	114.66	>512
Peptide-3	RRKSRFKSIVKKVVKHLSKGVKIYITPMF	64	**32**	**32**	**32**	**32**	**32**	45.94	>512
Peptide-4	GNWKTVWKKVRKAAKKLVGRRIRIVVQQLL	64	**32**	**32**	**32**	**32**	**32**	70.05	>512
Peptide-5	KWWKRLGRLAWRGPRKVAGSALKKLFKIGK	128	**32**	**32**	**32**	**16**	**32**	81.64	>512
Peptide-6	KSWSKSLRRLWNSLRKYGKKIARPIIWK	64	**16**	**32**	**16**	**16**	**16**	60	>512
Peptide-7 (Arcinin)	GRWRRVGRKLRTLGKSFGKVAHVAGKAIFA	64	**16**	**8**	**8**	**8**	**8**	50.84	>512
Peptide-8	RWLRPAIGRVIRWPRKIPGPIRRAIRKLR	64	**32**	**32**	**32**	**32**	**32**	113.1	>512
Peptide-9	KWNSRVGKRLKRLFKRIGKALHWLFQG	>128	64	>128	>128	>128	64	81.21	>512
Peptide-10	RWAGRLFRRLGKIARKVGKIKKQAQGGLK	>128	**32**	128	**16**	**8**	**32**	79.62	>512
Pexiganan	GIGKFLKKAKKFGKAFVKILKK	**32**	**16**	**8**	**8**	**8**	**8**	37.75	>512
Hydraganan-1	GVGKKLWFALKKPLGLVKFFKLL	**32**	**16**	**16**	**4**	**16**	**4**	40.53	>512
Melittin	GIGAVLKVLTTGLPALISWIKRKRQQ	**8**	**8**	**32**	**8**	**32**	**8**	5.32	NA
Colistin		>128	>128	2	1	2	1	NA	NA
Daptomycin		16	32	>128	>128	>128	>128	NA	NA

Values in bold indicate MICs of less than 32 μg/mL.

**Fig. 4:**
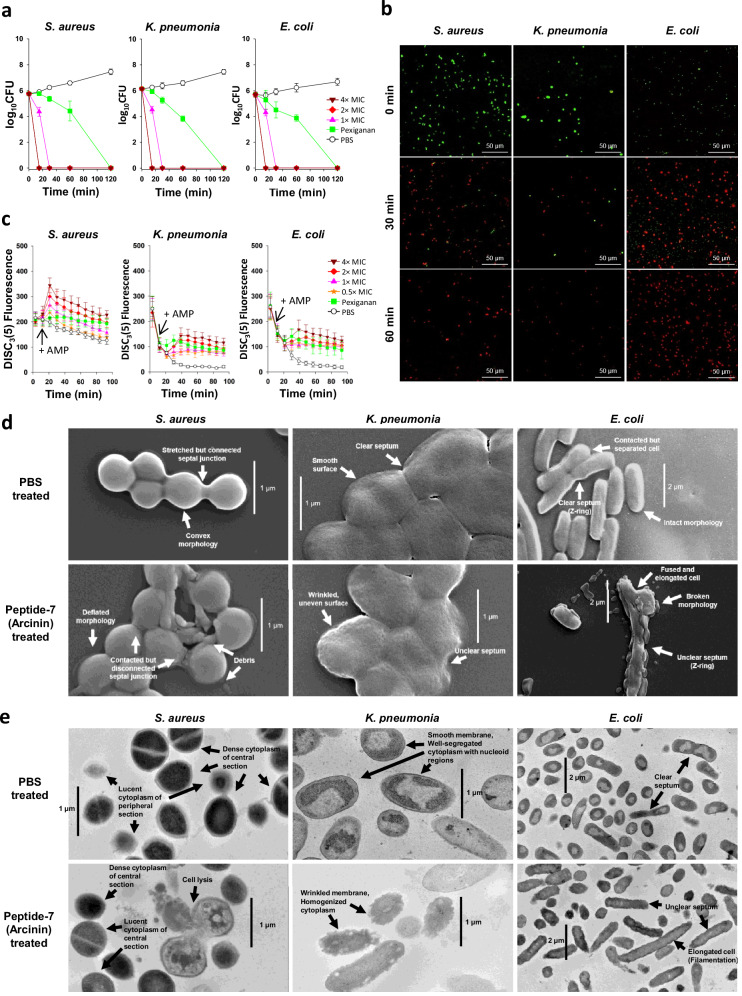
Experimental validation of AMP activity and mechanism of action. **a** Time-kill kinetics of *S. aureus*, *K. pneumoniae*, and *E. coli* treated with Arcinin at 1×, 2×, and 4× MIC. Data are presented as mean ± s.d. derived from *n* = 3 independent biological replicates. At 15 min, significance was determined by two-tailed unpaired t-tests between Arcinin (1x MIC) and PBS: *S. aureus* (t(4) = 7.28, *p* = 0.0019, Cohen’s d = −5.95, 95% CI: [−2.119, −0.949]), *K. pneumoniae* (t(4) = 10.36, *p* = 0.0005, Cohen’s d = −8.46, 95% CI: [−2.186, −1.262]), and *E. coli* (t(4) = 6.26*, p* = 0.0033, Cohen’s d = −5.11, 95% CI: [−1.905, −0.735]). These experiments were repeated three times independently with similar results. PBS and Pexiganan served as negative and positive controls, respectively. **b** Confocal fluorescence microscopy of SYTO9-stained live cells (green) and PI-stained damaged cells (red) in *S. aureus*, *K. pneumoniae*, and *E. coli* treated with Arcinin at 1× MIC. **c** Membrane depolarization assay using DiSC_3_(5) in *S. aureus*, *K. pneumoniae*, and *E. coli* treated with Arcinin at 0.5×, 1×, 2×, and 4× MIC. Data points represent the mean ± s.d. of *n* = 3 independent biological replicates. At 93 min, significance was determined by two-tailed unpaired t-tests between Arcinin (1x MIC) and PBS: *S. aureus* (t(4) = 2.44, *p* = 0.0712, Cohen’s d = 1.99, 95% CI: [−4.21, 65.54]), *K. pneumoniae* (t(4) = 7.5, *p* = 0.0017, Cohen’s d = 6.12, 95% CI: [33.68, 73.32]), and *E. coli* (t(4) = 8.97*, p* = 0.0009, Cohen’s d = 7.32, 95% CI: [57.01, 108.13]). These experiments were repeated three times independently with similar results. Pexiganan and PBS served as positive and negative controls, respectively. **d**, **e** Scanning electron microscopy and transmission electron microscopy images of *S. aureus*, *K. pneumoniae*, and *E. coli* treated with Arcinin at 1× MIC.

Arcinin also demonstrated favorable in vitro serum stability, maintaining an MIC of 32 μg/mL in the presence of 50% bovine serum for four ESKAPE species, a performance comparable to that of the reference AMP pexiganan, which has advanced to phase 3 clinical trials^38^ (Fig. 3b; Supplementary Table 4). This immediate stability against serum-associated challenges allows the peptide to overcome the first major hurdle faced by fast-acting agents. By maintaining effective concentrations in the presence of serum, Arcinin can exert rapid bactericidal activity (Fig. 4a) before longer-term clearance processes such as metabolism or excretion reduce its serum stability. All ten peptides exhibited promising safety profiles, with no significant hemolysis observed up to 512 μg/mL, and LC_50_ values in HEK293 cells comparable to those of published AMPs (Fig. 3c; Table 1; Supplementary Fig. 15). These findings suggest that the training set and weighting strategy inherently biased the generator toward lower-toxicity sequences, likely reflecting the therapeutic nature of many known AMPs.

### Molecular dynamics simulation infers the mechanism of action of Arcinin

To investigate Arcinin’s mechanism of action, we performed molecular dynamics (MD) simulations of peptide-membrane interactions. Arcinin’s α-helical structure in a bacterial membrane-mimetic environment (SDS micelles) was supported by its CD spectrum (Fig. 3d, e**;** Supplementary Fig. 16). Melittin, a well-characterized α-helical AMP, and a non-AMP peptide (one of the enterotoxins of *E. coli*; PDB ID: 7CSS) were included as positive and negative controls, respectively. All peptides attached to the membrane initially, and Arcinin was also observed to attach to the membrane in 100 mM NaCl solution (Supplementary Fig. 17). Although Arcinin and melittin both adopt a two-helix structure connected by a central loop, they penetrated the membrane through distinct mechanisms. In Arcinin, the first helix began to transition into an irregular structure, such as a loop or turn, at approximately 500 ns, while the second helix remained intact and penetrated the membrane (Supplementary Fig. 18a). In contrast, melittin retained its two-helix structure throughout the simulation and penetrated the membrane through its central loop (Supplementary Fig. 18b). Arcinin penetrated the membrane at 850 ns, similar to melittin at 750 ns. By contrast, most non-AMP molecules retained a similar overall structure and detached from the membrane without insertion (Supplementary Figs. 19, 20a, b). During penetration, peptide density in the inner leaflet increased significantly for both Arcinin and melittin (Fig. 3f; Supplementary Fig. 20c), indicating successful traversal of the bilayer. Membrane holes appeared after peptide penetration (Fig. 3f, g; Supplementary Figs. 20d, 21), and lipid density decreased significantly in both cases. Together, these results suggest that Arcinin, like melittin, penetrates and disrupts the lipid bilayer, leading to membrane destabilization and bacterial inhibition.

### Arcinin acts as a bacterial membrane disruptor

To further characterize Arcinin’s antimicrobial effect, we performed multiple experiments to probe its mechanism of action. Time-kill experiments against *S. aureus*, *K. pneumoniae*, and *E. coli* demonstrated potent, concentration-dependent bactericidal activity (Fig. 4a). At 2× and 4× MIC, Arcinin eradicated bacterial populations within 30–60 minutes, reducing viable counts below the detection limit, a rapid bactericidal effect typical of membrane-active AMPs. To confirm that cell death resulted from membrane disruption, we conducted live/dead staining using SYTO9 (green, stains all cells) and propidium iodide (red, penetrates cells with compromised membranes). Fluorescence microscopy after 30–60 minutes of peptide exposure revealed a marked increase in red-stained cells at higher concentrations (Fig. 4b), indicating that Arcinin rapidly permeabilized bacterial membranes.

We next assessed membrane depolarization using the potential-sensitive dye DiSC_3_(5) (Fig. 4c). Arcinin triggered a rapid and sustained increase in fluorescence in all three species, indicating collapse of membrane potential, a hallmark of membrane-targeting AMPs. *S. aureus* exhibited a stable baseline before peptide addition, whereas *K. pneumoniae* and *E. coli* showed mild baseline instability, a known artifact arising from this dye’s interaction with Gram-negative outer membranes. Nonetheless, all species exhibited immediate depolarization after Arcinin treatment.

Morphological effects were visualized by scanning electron microscopy (SEM) (Fig. 4d). In *S. aureus*, Arcinin-treated cells exhibited a deflated appearance (loss of turgor), abnormal septal separation, and disorganized debris, all consistent with membrane and cell-wall damage. In *E. coli*, cells lost their characteristic rod shape and appeared as amorphous fused structures with obliterated septa and abundant membranous debris, reflecting catastrophic envelope failure. By contrast, *K. pneumoniae* cells displayed subtler changes: the normally smooth capsular surface became wrinkled, and septa appeared faint. This discrepancy is notable because, despite identical MICs for *K. pneumoniae* and *E. coli* and similar depolarization profiles, the morphological endpoint in *K. pneumoniae* suggested a less overtly lytic death characterized primarily by surface alterations rather than wholesale lysis.

Transmission electron microscopy (TEM) provided additional insight into intracellular effects (Fig. 4e). In *S. aureus*, Arcinin caused overt lysis in some cells, whereas others remained structurally intact but displayed electron-lucent cytoplasm consistent with cytoplasmic leakage. In *K. pneumoniae*, TEM confirmed surface wrinkling and revealed homogenization of the cytoplasm. In *E. coli*, Arcinin induced similar cytoplasmic homogenization, with the most striking phenotype being filamentation, that is, highly elongated cells with indistinct septa. Comparative analysis of SEM and TEM images (Supplementary Figs. 22, 23) further highlighted these differences: SEM revealed relatively subtle surface wrinkling, whereas TEM showed profound loss of internal integrity and cytoplasmic homogenization.

Taken together, these findings indicate that Arcinin’s action is not consistent with wholesale lysis expected from a detergent-like “carpet” model. Instead, they support a more specific pore-forming mechanism in which limited membrane lesions trigger catastrophic failure of internal homeostasis. Although Arcinin was not explicitly engineered for a particular mode of action, it emerged as a potent AMP that combines low cytotoxicity with a targeted, membrane-penetrating mechanism that effectively kills bacteria.

### Arcinin demonstrates therapeutic efficacy in a murine wound infection model

**Fig. 5:**
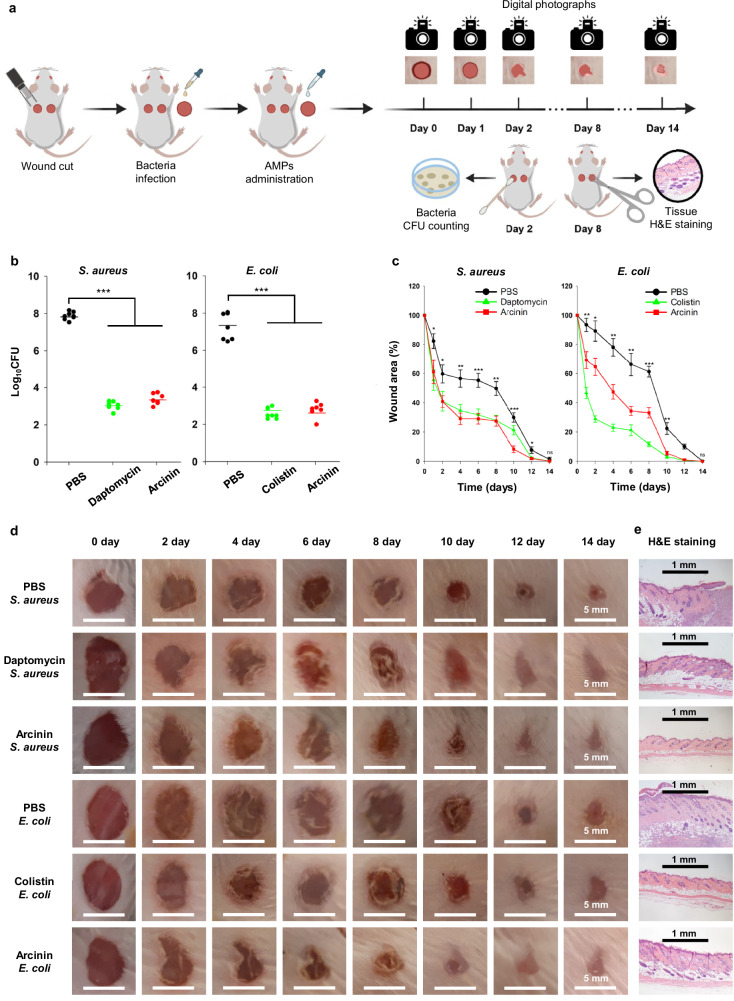
In vivo therapeutic efficacy of AMP in a wound infection model. **a** Schematic illustration of the murine full-thickness excisional wound model, outlining the experimental timeline for infection and topical treatment. **b** Bacterial burden in wound tissues at Day 2 post-infection. Data are presented as mean ± s.d. (*n* = 8 mice per group). Statistical significance was determined by one-way ANOVA. Arcinin treatment reduced viable bacterial counts by more than 4-log for both *S. aureus* (t(21) = 18.72, *p* < 0.0001, Cohen’s d = 8.09, 95% CI: [4.05, 5.07]) and *E. coli* (t(21) = 37.91, *p* < 0.0001, Cohen’s d = 18.33, 95% CI: [4.23, 4.72]). **c** Wound closure (%) over time in *S. aureus* and *E. coli* models. Data represent mean ± s.e.m. (*n* = 7–8 mice per group). The Arcinin group (red) significantly outperformed the PBS group (black) from Day 1 through Day 12. Statistical significance was assessed by two-way repeated-measure ANOVA. Statistical significance between Arcinin and PBS on day 6: *S. aureus* (t(13) = 4.51, *p* = 0.0006, Cohen’s d = 2.33, 95% CI: [13.69, 38.91]), and *E. coli* (t(12) = 3.82, *p* = 0.0024, Cohen’s d = 2.04, 95% CI: [13.73, 50.2]). **d** Representative digital photographs of infected wounds over the 14-day healing period across treatment groups. **e** Histopathological analysis of H&E-stained wound sections harvested on Day 8. The Arcinin group showed enhanced re-epithelialization and reduced inflammatory cell infiltration compared to the PBS group. In *E. coli*-infected wounds, the Arcinin group displayed a more cyanotic appearance compared to *S. aureus*-infected tissues, likely due to endotoxin induced by the *E. coli* outer membrane disruption.

To assess the therapeutic potential of Arcinin in a complex physiological environment, we employed a murine full-thickness excisional wound model infected with either *S. aureus* or *E. coli* (Fig. 5a). The primary objective of this in vivo validation was to evaluate Arcinin’s capacity to resolve localized infection, which remains the fundamental bottleneck in infected wound recovery. Accordingly, we prioritized the reduction of bacterial burden. Treatment with Arcinin achieved a 4.56 Log_10_CFU reduction in viable bacterial counts for *S. aureus* (*p* < 0.0001) and a 4.47 Log_10_CFU reduction for *E. coli* (*p* < 0.0001), demonstrating significantly higher efficacy than the control groups (Fig. 5b). This profound sterilization confirms that the ARCADIAMP-generated lead retains its potent bactericidal activity even within a complex physiological milieu challenged by endogenous proteases. Building upon this antimicrobial clearance, we monitored wound recovery as a supporting functional readout (Fig. 5c, d). In this non-splinted excisional model for antimicrobial evaluation, the Arcinin group demonstrated a significant lead in recovery, achieving approximately 71% wound closure by Day 6 compared to 45% in the negative control group for *S. aureus* (*p* = 0.0006) and 66% closure compared to 34% for *E. coli* (*p* = 0.0024), demonstrating a two-day lead in the healing timeline compared to the negative control (Fig. 5c). While tissue contraction is an inherent component of the murine healing process in this model, the superior macroscopic recovery profile (Fig. 5d) was further corroborated by histopathological evidence (Fig. 5e). H&E staining on Day 8 revealed enhanced re-epithelialization and significantly reduced inflammatory cell infiltration in the Arcinin group compared to the PBS control (Fig. 5e). Taken together, these multi-level observations—from bacterial clearance to tissue-level regeneration—underscore Arcinin’s capacity to successfully transition the wound from a stalled inflammatory state to the proliferative phase of healing.

## Discussion

To address the need for antibiotic discovery that combines broad activity with favorable safety and exposure, we present an integrated platform that co-optimizes antimicrobial potency and toxicity by coupling an iterative-learning discrete diffusion generator with a therapeutic-index-weighted training scheme, a two-stage activity classifier, and a novelty filter, followed by targeted wet-lab and mechanistic assays. Across the ten synthesized candidates, all showed measurable antibacterial activity, and eight exhibited MIC ≤ 32 μg/mL against at least four ESKAPE pathogens, while also showing negligible hemolysis and low HEK293 toxicity. The lead peptide, Arcinin, additionally retained activity in 50% serum. These results indicate that explicitly rewarding the therapeutic index during training can steer generation toward clinically promising sequences rather than merely potent ones.

Several methodological advances contributed to this outcome. First, the therapeutic-index-weighted iterative-learning diffusion loop improved sequence novelty while maintaining potency, suggesting that retraining on high-scoring, low-toxicity peptides shifts the discovery platform toward favorable regions of sequence space. Second, the two-stage architecture, comprising AMP detection followed by strong-AMP prioritization, delivered higher precision-recall performance and better top-k MIC ranking than the baselines, thereby reducing synthesis waste and increasing the wet-lab hit rate. Molecular dynamics combined with experimental validation further clarified the mechanism of action and supported a pore-forming membrane-disruption model rather than a carpet-like lysis mechanism, providing mechanistic plausibility for the rapid time-kill kinetics that we observed. Whether Arcinin engages membranes in a mechanistically distinct manner relative to other membrane-active AMPs remains to be determined by future studies. While this targeted disruption suggests a robust barrier to resistance, formal experimental evolution studies remain necessary to define the long-term resistance landscape of Arcinin and its clinical potential. Because the framework is modality- and objective-agnostic, it offers a modular architecture for AI-assisted discovery of biologics beyond AMPs, including cell-penetrating and immunomodulatory peptides.

By combining these components with a modified training process, the pipeline achieved strong screening performance. Among the ten AMP candidates selected, all exhibited low toxicity. Eight demonstrated high antibacterial activity (MIC 8–32 μg/mL) against five ESKAPE species in MHB. Two of these candidates maintained strong activity in both CaMHB and the in vitro serum stability assay. Notably, Arcinin showed strong antibacterial activity in both MHB and CaMHB, together with favorable in vitro serum stability. Arcinin’s serum-retained MIC, RBC LC_50_ > 512 μg/mL, and low HEK293 cytotoxicity compare favorably with the published peptide controls included in our benchmarking panel (Fig. 3). Arcinin’s therapeutic potential was further underscored by its robust performance in the murine full-thickness excisional wound model. This environment, which closely mimics the physiological milieu of human diabetic foot ulcers and surgical-site infections, challenges peptides with interstitial fluid containing approximately twofold higher calcium and magnesium concentrations than CaMHB. Together with endogenous proteases and exudate sequestration, these factors represent a substantial biological hurdle for pharmacokinetic stability. Arcinin effectively navigated these challenges, with treated wounds closing significantly faster than PBS controls and performing comparably to clinical standards such as colistin and daptomycin. These results indicate that our therapeutic-index-weighted optimization successfully captured the delicate balance between potent bactericidal activity and the stability required for efficacy in a living system. On the computational side, expanding the training data toward species-specific objectives and incorporating chemistry-aware constraints, such as D-amino acids or non-canonical residues, could further improve both performance and reliability. We envision that continued refinement of this framework could enable the discovery of clinical-grade peptides with broad bioactivity, thereby substantially expanding our options for addressing urgent health challenges such as antibiotic resistance.

## Methods

### Dataset for AMP screening pipeline development

We collected 13,950 AMP sequences containing only the 20 standard L-form amino acids^44^ and targeting ESKAPE pathogens^39^ from the DBAASP database^40^ for training the generative model and antibacterial activity predictors. Of these, 8,496 sequences targeted *E. coli* and were used to train the activity prediction models, whereas the full set was used to train the generative model. To test the antibacterial activity predictor, we collected 195 AMP sequences from the CAMPR4 database^13^ that were not present in our training set, all composed of standard amino acids and targeting *E. coli* ATCC 25922. We also compiled a large set of putative non-AMP peptides to augment the training and test sets. The initial non-AMP pool consisted of 202,166 sequences filtered from UniProt^45^ according to criteria described by Gabere and Noble^46^: peptides annotated as “antibacterial” or localized to the Golgi, cytoplasm, endoplasmic reticulum, or mitochondria were excluded so that the remaining sequences were presumed non-antimicrobial. The characteristics of these peptide datasets are summarized in Supplementary Fig. 30–35, including the composition of the curated dataset and the MIC distribution of *E. coli*-targeting AMPs (Supplementary Fig. 30), activity-value comparisons across experimental conditions, strains, and media (Supplementary Fig. 31), peptide-length distributions of the training and test sets for the generative model and the antibacterial activity classifiers (Supplementary Figs. 32, 33), and the predicted secondary structure, amphiphilicity, and charge of AMP sequences in the training and test sets (Supplementary Figs. 34, 35).

### The active iterative learning procedure of the generative model

We used the 13,950 AMP sequences to train the generative model, which was based on EvoDiff-D3PM^35^ — a recently published protein language foundation model using discrete diffusion (D3PM)^36^ (Supplementary Fig. 2a). Rather than fine-tuning a pretrained model, we trained the model from random initialization because we found that direct training generated AMP candidates with stronger predicted antibacterial activity. In addition, all 13,950 AMP sequences were assigned equal weights during this initial training stage.

We further improved the generative model by augmenting its training set with de novo sequences it generated. Specifically, after initial training on the 13,950 AMPs without sample weighting, we generated ~100,000 sequences of length 20 residues as a representative average AMP length. After applying the Huang et al.^22^ regression model and a 50% sequence similarity threshold to the initial training set, 16,334 peptides possessed an MIC < 32 μg/mL and were added to the initial training data (Supplementary Fig. 2d). We then defined sample-specific weights based on each peptide’s antimicrobial activity and toxicity. For all peptides with known data (the 13,950 DBAASP AMPs), we gathered MIC values against various ESKAPE species together with hemolytic toxicity measurements (LC_50_, the concentration causing 50% red blood cell lysis). We computed a Log_10_ therapeutic index for each peptide as Log_10_(LC_50_) - Log_10_(MIC) for each species. This value was mapped to an initial weight through a piecewise function and then normalized across species to yield a final weight (Supplementary Fig. 7). For peptides in the augmented set, or for AMPs missing MIC or LC_50_ data, we applied default values (Log_10_(MIC) = 2.0, corresponding to 100 μg/mL; Log_10_(LC_50_) = 2.4, an average toxicity value) so that every sample had a defined weight during second-iteration training (Supplementary Fig. 2e).

To evaluate the benefit of additional training iterations beyond the main two-iteration pipeline, we performed sequential training in which the coarse-grained classifier and the sequence novelty filter were used to progressively select generated candidates for incorporation into each subsequent training set (Supplementary Fig. 2f). Five iterations were conducted in total: one initial training round followed by four successive augmentation rounds. At each iteration, candidate sequences were generated while preserving the length distribution of the training dataset within the 10–50 amino acid range. Augmented sequences were selected using the updated coarse-grained classifier with a probability threshold of ≥ 0.99998, together with novelty constraints of ≤ 0.50 identity and ≤ 0.45 similarity relative to known AMPs. For shorter peptides (10–13 amino acids), more permissive thresholds were applied (probability ≥ 0.999975; identity ≤ 0.50; similarity ≤ 0.70) to account for the reduced sequence diversity inherent to this length range. The resulting augmented datasets progressively expanded the training set — approximately doubling it at each round — while maintaining the same length distribution across the full 10–50 amino acid range. We compared the resulting model with several alternatives, including the OADM-based model from the EvoDiff study^35^, two published models, BroadAMP-GPT^42^ (GPT-based) and Pep-Diffusion^43^ (latent-space diffusion), and versions of our model trained with varying numbers of augmented-training iterations.

### The training procedure of the antibacterial activity classifiers

We implemented a two-stage ESM2-based antibacterial activity classifier. The first classifier (coarse-grained) distinguished AMPs from non-AMPs, whereas the second (fine-grained) identified strong AMPs (MIC < 8 μg/mL, targeting *E. coli*). t-SNE^47^ plots showed that some AMPs could be separated from non-AMPs (Supplementary Fig. 27a). However, no distinct clusters of strong AMPs with MIC < 8 μg/mL were observed (Supplementary Fig. 27b). Both classifiers were based on pretrained ESM-2 models^37^ (MetaAI) obtained from the official GitHub repository and fine-tuned on different training sets (Fig. 1b). For training and testing the coarse-grained classifier, we anticipated that most generated candidates would be non-AMPs; therefore, we augmented our datasets with randomly selected non-AMP sequences from the initial pool, matched to the AMP length distribution. After evaluating different levels of non-AMP augmentation (Supplementary Figs. 28 and 29; Supplementary Table 5), we chose to include 93,408 non-AMP sequences in the training set together with 8,496 E. coli-targeting AMPs, and 19,143 non-AMP sequences in the test set, corresponding to an approximately 100:1 ratio of non-AMPs to AMPs. The coarse-grained classifier was then compared against three published AMP classification methods, AMPlify^24^, PepNet^26^, and Diff-AMP^27^, including available configurations that used peptide-structure-related features (for PepNet) or balanced versus imbalanced training sets (for AMPlify). For the fine-grained classifier, we excluded the augmented non-AMPs and labeled AMPs with MIC < 8 μg/mL as positive examples. We then compared its performance with that of the coarse-grained classifier and the ensemble MIC regression model, the best-performing model for distinguishing AMPs from non-AMPs, to assess performance in screening strong AMPs (MIC < 8 μg/mL).

### Sequence novelty evaluation

To ensure novelty among generated sequences, we filtered candidate AMPs based on similarity to known peptides. Each generated sequence was aligned to a reference set of 27,636 well-characterized AMPs compiled from five databases (DBAASP, CAMPR4, DBAMP^48^, DRAMP^49^, and APD^50^) using the Needleman-Wunsch global alignment algorithm^51^ with a BLOSUM62 substitution matrix^41^ and specified gap penalties (−11 for gap opening, -1 for gap extension internally, and 0 for terminal gaps). For each generated peptide, we identified the most similar known AMP and recorded the alignment score and percent identity (Supplementary Fig. 4). We normalized the alignment score to a [0,1] scale by dividing it by the candidate’s self-alignment score, such that a sequence aligned to itself yields 1.0. We also calculated an identity score, defined as the fraction of identical residues within the aligned region from the first to the last aligned residue. A candidate was considered novel if its normalized alignment score was <0.45 and its identity was <0.65 relative to the closest known AMP.

### Molecular dynamics simulation

Molecular dynamics (MD) simulations were conducted to examine peptide-membrane interactions. The 3D structure of Arcinin was predicted with PEP-FOLD4^52^, whereas Melittin (PDB ID: 2MLT) and a non-AMP peptide (heat-stable enterotoxin; PDB ID: 7CSS) from the Protein Data Bank^53^ served as positive and negative controls, respectively.

Simulation systems containing peptides, lipid bilayers, water, and ions were prepared using CHARMM-GUI^54^. To first examine peptide-membrane association, we simulated Arcinin attachment to the bilayer. The simulation box was set to 8 × 8 × 45 nm^3^, with 20 nm water layers above and below the bilayer, which is thick enough to prevent peptides from artificially appearing on the opposite side of the box and reaching the inner leaflet through periodic boundary conditions. The bilayer comprised 74 DMPC (1,2-ditetradecanoyl-sn-glycero-3-phosphocholine) molecules per leaflet (148 total), which is thinner than the standard CHARMM-GUI^54^ membrane configuration for the same area (100 DMPC molecules per leaflet). We used this thinner DMPC membrane and increased the temperature to 333 K, following Ulmschneider et al.^55^, to accelerate penetration. This setup is justified by the stability of peptide-membrane systems at elevated temperatures (up to 95 °C)^51,52,55–57^. GROMACS^58^ input files were generated with the CHARMM36m^59^ force fields, followed by energy minimization, six equilibration stages, and a production run. To investigate membrane penetration, Arcinin, melittin, and the non-AMP peptide were positioned 2 nm from the bilayer midplane. Molecule counts (4 Arcinin, 7 melittin, and 25 non-AMP molecules) were adjusted to approximate equivalent outer-leaflet coverage (~8×8 nm²). All systems were minimized, equilibrated, and simulated for 1 μs using GROMACS 2024.3 on Jetstream2^60^ through ACCESS^61^ resources.

### In vitro validation

#### Measurement of MIC in ESKAPE pathogens

The following ESKAPE pathogens were used as representative test organisms: *E. faecalis* ATCC 29212, *S. aureus* ATCC 25923, *K. pneumoniae* ATCC 700603, *A. baumannii* ATCC 19606, *P. aeruginosa* ATCC 27853, and *E. coli* ATCC 25922. Bacterial stock cultures were thawed and subcultured for three days in Mueller-Hinton broth (MHB; BD Difco) or cation-adjusted MHB (CaMHB) before the assay. On the day of the experiment, each culture was diluted to ~1 × 10^6^ CFU/mL in the appropriate broth. Peptide solutions were prepared at 256 μg/mL in the same medium and serially diluted two-fold. For each dilution, 50 μL of bacterial suspension was mixed with 50 μL of diluted peptide in a 96-well plate, yielding a final bacterial density of ~5 × 10^5^ CFU/mL per well. Plates were incubated at 37 °C for 18 hours. Bacterial growth was then assessed by measuring absorbance at 600 nm with a Synergy H1 microplate reader (BioTek). The MIC was defined as the lowest peptide concentration producing an OD600 ≤ 0.060. This conservative cutoff was chosen to account for empirical baseline fluctuations in sterile media (typically 0.030–0.060), ensuring that any reported MIC represents complete growth inhibition indistinguishable from background noise. Each experiment was performed in triplicate.

#### In vitro serum stability assay

Bacterial stocks were thawed and subcultured in cation-adjusted Mueller-Hinton broth (CaMHB) for three consecutive days before the assay. On the day of testing, bacterial cultures were diluted to a final concentration of 1 × 10^6^ CFU/mL in a 1:1 mixture of CaMHB and fetal bovine serum (FBS; Gibco, USA). AMP solutions were initially prepared at 256 μg/mL and serially diluted eight times in the same CaMHB-FBS mixture. A total of 50 μL of bacterial solution and 50 μL of AMP solution were combined in each well of a 96-well plate, resulting in a final bacterial concentration of 5 × 10^5^ CFU/mL. Plates were incubated at 37 °C for 18 hours. Bacterial growth was assessed by measuring absorbance at 600 nm using a Synergy H1 microplate reader (BioTek, USA). MIC values were defined as the lowest AMP concentration that completely inhibited bacterial growth. All experiments were conducted in triplicate.

#### Cytotoxicity assay in HEK293 cells

HEK293 cells were cultured in Dulbecco’s Modified Eagle Medium (DMEM) with 10% fetal bovine serum and 50 μg/mL gentamicin. Cells were thawed and passaged three times prior to the assay. On day 1, cells were adjusted to 100,000 cells/mL, and 100 μL of this suspension (~10,000 cells) was seeded into each well of a 96-well culture plate. Cells were incubated overnight at 37 °C with 5% CO_2_. On day 2, peptide solutions were prepared at double the desired final concentrations in culture medium. The medium in each well was gently aspirated down to 50 μL, and 50 μL of peptide solution was added to yield a final volume of 100 μL per well. Cells were incubated with peptides for 24 h. Melittin and DMSO were included as positive controls to confirm assay sensitivity to cell death; untreated cells served as the negative control. On day 3, 10 μL of medium was removed from each well and replaced with 10 μL of WST-1 reagent (Abcam Cell Proliferation Assay Kit). After 2 hours of incubation at 37 °C, absorbance was measured with a plate reader to assess cell viability, following the manufacturer’s WST-1 assay instructions.

#### Hemolysis assay in human red blood cells

Within four days of receipt, human-packed red blood cells (Zen-Bio, USA) were used for hemolysis testing. Cells were washed three times with PBS by centrifugation at 4000 × *g* for 15 minutes per wash. The washed RBCs were resuspended in PBS to 16% (v/v). Peptide stock solutions were serially diluted in PBS. Next, 100 μL of 16% RBC suspension was mixed with 100 μL of each peptide dilution in a 96-well plate, yielding final conditions of 8% RBC. For the technical positive and negative controls, 1% Triton X-100 and PBS were used to define 100% and 0% lysis, respectively. After incubation for 1 hour at 37 °C, plates were centrifuged at 1000 × g for 5 minutes. From each well, 100 μL of supernatant was carefully transferred to a new plate without disturbing the cell pellet. Free hemoglobin in the supernatant was measured at 571 nm using a Synergy HTX microplate reader (BioTek). Each condition was tested in triplicate. The LC_50_ (peptide concentration causing 50% hemolysis) was determined by fitting a four-parameter logistic curve to the hemolysis-versus-concentration data using the AAT Bioquest online calculator (https://www.aatbio.com/tools/ld50-calculator).

### In vivo validation

#### Animals and ethics statement

All animal experiments were approved by the Institutional Animal Care and Use Committee (IACUC) of the University of California, Davis. Male inbred BALB/c mice (BALB/cAnNCrl) were purchased from Charles River Laboratories (Hollister, CA, USA) at 7–8 weeks of age. Following an acclimation period, mice at 8–9 weeks of age were used for all in vivo studies. Animals were housed in a pathogen-free facility at the University of California, Davis, under a standard 12:12-hour light/dark cycle. The ambient temperature was maintained at 68 °F to 79 °F with a relative humidity of 30–70%, and all mice were provided ad libitum access to a standard laboratory diet and water.

#### Murine infected wound healing model

To evaluate the in vivo therapeutic efficacy of the candidate AMPs, we established a full-thickness excisional wound model (*n* = 7–8 per group). Mice were anesthetized with 1–5% isoflurane inhalation. Pre-emptive analgesia was administered subcutaneously (meloxicam, 5 mg/kg), and dorsal hair was removed using electric clippers. The surgical site was sterilized with povidone-iodine and 70% ethanol. A local anesthetic (lidocaine, up to 7 mg/kg) was injected subcutaneously around the wound area. Two full-thickness circular excision wounds (6 mm in diameter) were created on the dorsal midline using a sterile biopsy punch. The wounds were inoculated with 10^7^ CFU/mL of *E. coli* or *S. aureus* suspension. Two hours post-infection, the wounds were allowed to dry and then treated topically with either 10 μL of PBS (negative control), 10 μL of clinical AMPs (5 mg/kg; positive control: colistin for *E. coli* and daptomycin for *S. aureus*), or 10 μL of Arcinin (5 mg/kg; candidate AMP). All peptides were dissolved in PBS. After infection, the wounds were treated topically with either PBS (negative control), clinical AMPs (positive control: colistin for *E. coli* and daptomycin for *S. aureus*), or Arcinin (candidate AMP). The wound healing progress was monitored for 14 days post-surgery. Digital photographs were taken together with a reference scale, and the wound area was quantified using ImageJ software (NIH, USA). The percentage of wound closure was calculated as ((A0 - At) / A0) × 100, where A0 is the initial wound area on Day 0 (the day of surgery) and At is the wound area on day t.

#### Sample collection and histopathological analysis

On Day 2 post-infection, wound surface samples were collected from the eight experimental mice using sterile swabs to verify bacterial colonization. The swabs were streaked onto MH agar plates and incubated at 37 °C for 24 hours to assess CFU presence. In addition, one randomly selected mouse per group (*n* = 1) was euthanized on Day 8 specifically for histopathological analysis and qualitative imaging (SEM, TEM, and confocal microscopy). To ensure morphological consistency, representative images were selected from at least three different fields of view per biological sample. Harvested wound tissues were fixed in 10% neutral buffered formalin, embedded in paraffin, and processed for hematoxylin and eosin staining. To ensure an objective assessment and minimize investigator bias, all wound areas were quantified using ImageJ software (NIH, USA) based on standardized digital photographs. The initial sample size for the murine infected wound healing model was *n* = 8 mice per group. To ensure data integrity, outliers were identified using Grubbs’ test (α = 0.05). A single statistical outlier was identified and removed from five of the six experimental groups to preserve the continuity of the healing profile. No significant outliers were detected in the *S. aureus*-Arcinin group, which maintained its original sample size. Consequently, the analytical sample size was *n* = 7 for five groups and *n* = 8 for the *S. aureus*-Arcinin group. Statistical significance for the wound-closure time course was determined using two-way repeated-measures ANOVA, while the quantitative reduction in bacterial burden (Log_10_CFU) was analyzed using one-way ANOVA.

### AMP action mechanism demonstration

#### Bacterial killing dynamics assay

*S. aureus* ATCC 25923, *K. pneumoniae* ATCC 700603, and *E. coli* ATCC 25922 were evaluated for time-kill kinetics. Bacterial cultures were prepared as described above and diluted to ~1 × 10^6^ CFU/mL in MHB on the day of assay. In a 96-well plate, 50 μL of bacterial suspension was combined with 50 μL of peptide solution at the specified multiple of its MIC. The final bacterial concentration was ~5 × 10^5^ CFU/mL. Arcinin was tested at 1×, 2×, and 4× MIC. At time points 0, 15, 30, 60, and 120 minutes, samples from each well were serially diluted and plated on MHB agar, then incubated at 37 °C for 18 hours to enumerate surviving colonies. Pexiganan at 1× MIC served as a positive control. Each experiment was performed in triplicate.

#### Membrane permeabilization assay

*S. aureus* ATCC 25923, *K. pneumoniae* ATCC 700603, and *E. coli* ATCC 25922 stocks were thawed and subcultured three times in MHB over three days before the assay. On the day of the experiment, mid-log-phase bacterial cultures were harvested, washed twice with PBS, and diluted to OD_600_ = 0.1. DiSC_3_(5) dye was added to a final concentration of 2 μM, and cells were incubated at room temperature in the dark for 30 minutes to allow dye loading and membrane potential stabilization. Fluorescence (excitation/emission: 622/670 nm) was then measured every 60 seconds using a Synergy H1 microplate reader (BioTek, USA). After 10 minutes, the reading was paused, and peptide samples were added to the wells at final concentrations corresponding to 0.5×, 1×, 2×, and 4× MIC. Measurements then resumed, and fluorescence was monitored continuously for up to 100 minutes. Pexiganan at 1× MIC was used as the positive control, and PBS as the negative control. All measurements were performed in triplicate.

#### Confocal fluorescence microscopy

*S. aureus* ATCC 25923, *K. pneumoniae* ATCC 700603, and *E. coli* ATCC 25922 cultures in mid-log phase were harvested and washed twice with 10 mM Tris-HCl buffer (pH 7.5). The bacterial pellets were resuspended and diluted in the same buffer to a final optical density of OD_600_ ≈ 0.5 (>1 × 10^8^ CFU/mL). The bacterial suspension was treated with Arcinin at a final concentration of 1× MIC and incubated for 30 minutes at room temperature. Following treatment, the cells were stained for 15 minutes in the dark with a live/dead staining solution containing SYTO™ 9 and propidium iodide (PI). A 10 µL aliquot of the stained suspension was then mounted on a glass slide. Fluorescent images were acquired using a Zeiss 980 Laser Scanning Microscope with Airyscan 2 (Zeiss, Germany). Image processing and analysis were performed using ImageJ software (NIH, USA).

#### Scanning electron microscopy

Mid-log-phase cultures of *S. aureus* ATCC 25923, *K. pneumoniae* ATCC 700603, and *E. coli* ATCC 25922 were harvested, washed twice with assay buffer (PBS, pH 7.2), and diluted to an OD_600_ of 0.1. Bacteria were deposited onto glass coverslips and incubated for 10 minutes. Peptide samples were then added to the coverslips at final concentrations corresponding to 1× MIC and incubated for 30 minutes at room temperature. For fixation, samples were treated with 4% paraformaldehyde and 1% osmium tetroxide (Biolyst Scientific, Hatfield, PA, USA), washed with ultrapure water, and dried. Dried samples were sputter-coated with gold. Bacterial morphology was imaged using a ThermoFisher Quattro S Environmental Scanning Electron Microscope (Thermo Fisher Scientific, Waltham, MA, USA). Imaging was performed in high-vacuum mode using an Everhart-Thornley detector at an accelerating voltage of 15 kV and recorded at magnifications of 20,000× and 50,000×.

#### Transmission electron microscopy

Mid-log-phase cultures of *S. aureus* ATCC 25923, *K. pneumoniae* ATCC 700603, and *E. coli* ATCC 25922 were harvested, washed twice with assay buffer (PBS, pH 7.2), and diluted to an OD_600_ of 0.1. Bacteria were deposited onto glass coverslips and incubated for 10 minutes. Peptide samples were then added to the coverslips at final concentrations corresponding to 1× MIC and incubated for 30 minutes at room temperature. For fixation, samples were treated with 4% paraformaldehyde and 1% osmium tetroxide (Electron Microscopy Science, Hatfield, PA, USA), then washed with ultrapure water. Following fixation, cell pellets were dehydrated through a graded ethanol and acetone series, embedded in Embed 812 resin 14900 (Electron Microscopy Science, Hatfield, PA, USA), and polymerized at 60 °C for 48 h. Ultra-thin sections (70–90 nm) were cut using an ultramicrotome, mounted on copper grids, and post-stained with uranyl acetate and lead citrate before imaging with a TFS Glacios Cryo-Transmission Electron Microscope (Thermo Fisher Scientific, Waltham, MA, USA). Images were recorded at magnifications of 4300× and 13,500×.

### Reporting summary

Further information on research design is available in the Nature Portfolio Reporting Summary linked to this article.

## Supplementary information

Supplementary Information

Reporting Summary

Transparent Peer Review file

## Source data

Source Data

## Data Availability

The training and testing data of the models in this study were deposited in the corresponding GitHub repository: https://github.com/IBPA/ARCADIAMP. All the processed data shown in the Figures and Tables are available in the source data files. The protein structures used in this study are available in the PDB database under accession codes 2MLT and 7CSS. Source data are provided with this paper.

## Code availability

The code of the pipeline, including the generative model, antibacterial activity classifiers, and sequence novelty evaluation module, was published in the corresponding GitHub repository: https://github.com/IBPA/ARCADIAMP.
