# Supplementary material for: Discovery of potent low-toxicity antimicrobial peptides through diffusion modeling
Source: Nat Commun. 2026 Jul 7;17:8396. doi: 10.1038/s41467-026-75030-8 (PMC13473622; doi:10.1038/s41467-026-75030-8)
Supplement: Supplementary file 1 [file 41467_2026_75030_MOESM1_ESM.pdf]

Discovery of potent low-toxicity antimicrobial peptides through diffusion modeling

Konstantinos Markakis[1,2,3†], Shanghyeon Kim[2,3†], Cheng-En Tan[1,2,3], and Ilias Tagkopoulos[1,2,3*]

[1]*Department of Computer Science, University of California, Davis, Davis, California, 95616 United States*

[2]*Genome Center, University of California, Davis, Davis, California, 95616 United States*

[3]*USDA/NSF AI Institute for Next Generation Food Systems (AIFS), University of California, Davis, Davis, California, 95616 United States*

† These authors contributed equally to this work.

[*]Corresponding author: itagkopoulos@ucdavis.edu

SUPPLEMENTARY INFORMATION

Supplementary Information

1. Dataset

We initially selected five major AMP databases - DBAASP[1], CAMPR4[2], DBAMP[3], DRAMP[4], and APD[5], and downloaded their AMP sequences (accessed October 2023). The metadata in DBAASP is well organized in JSON format, which allowed us to curate experimental conditions, including bacterial strain, medium, and antibacterial activity type (MIC or MBC). Therefore, we used AMP sequences and corresponding antibacterial activity values from DBAASP as the source of training data. The CAMPR4 database, which contained the largest number of sequences at the time of data collection, was selected as the source of testing data. AMP sequences composed of the 20 standard L-form amino acids from all databases were compiled as well-known AMPs for evaluating the sequence novelty of screened candidates (**Supplementary Table 6**).

1.1 Preprocessing

We prepared the training dataset from DBAASP and the testing dataset from CAMPR4. From DBAASP, metadata were semi-automatically extracted from the JSON file[6]. Antibacterial activity entries included target species/strain, activity types, and activity values with units, whereas toxicity entries included target cell type, percentage of cell death or lysis, and peptide concentration (**Supplementary Table 7-10**).

All AMP sequences composed of the 20 standard L-form amino acids from DBAASP and targeting ESKAPE[7] species were used to train the generative model (final dataset size: 13,950 AMPs). For training the antibacterial activity predictors, we extracted MIC and MBC values. Antibacterial activity values reported in inconsistent units, such as μM, were converted to μg/mL using estimated molecular weights (from Pyteomics[8]), irrespective of

terminal modifications. Because only a small subset of AMPs carried terminal modifications, and because most modifications involved small chemical groups, terminal modifications were not expected to substantially alter molecular weight. Among the 1,415 strong AMPs (E. coli MIC < 10 μg/mL), 93% lacked N-terminal modification, whereas 40% and 59% either lacked C-terminal modification or carried an amino (-NH_2) modification, respectively. Thus, sequences that differed only in terminal modifications were treated as identical, and their antibacterial activity values were pooled.

For experimental validation, we applied no N-terminal modification and used an amino group (-NH_2) modification at the C-terminus based on these observations. For MIC or MBC values reported as ranges, the upper bound was used. Values reported as ">" (indicating no inhibition at the tested concentrations) were assigned the maximum MIC observed in the training set (13,112 μg/mL). When multiple values were reported for the same peptide, the average was calculated.

We curated 8,496 AMPs from DBAASP targeting E. coli for training the activity predictors, encompassing 18,866 activity measurements. Of these, 81% were MIC_{100} values (**Supplementary Figure 30a**). Mueller Hinton Broth (MHB)[9] and strain ATCC 25922 were the most frequent assay conditions, accounting for 55% and 40% of measurements, respectively (**Supplementary Figure 30b,c**). Accordingly, these conditions (and cation-adjusted MHB, CaMHB, per CLSI[10]) were chosen as reference standards for *in vitro* validation.

Significant variability was observed across strains, activity types, and media (**Supplementary Table 7-9**; **Supplementary Figure 31**). Thus, we prepared unnormalized dataset and three normalized datasets where activity values under minority

strains, activity types, and media were adjusted relative to majority conditions, if significantly different (**Supplementary Table 7-9**).

For the CAMPR4 dataset (testing set), metadata was less complete. We extracted target species/strain, activity values, and reference information from HTML pages[11], resolving inconsistencies manually. We selected 195 *E. coli* AMPs with MIC_{100} measurements, absent from DBAASP, as independent test data. The MIC_{100} distributions of strong/medium AMPs (< 256 μg/mL) from DBAASP (unnormalized) and CAMPR4 were similar (**Supplementary Figure 30d,e**).

We then summarized the peptide characteristics of the dataset. Most peptides were no longer than 25 residues. The proportions of sequences with lengths of 25 residues or fewer were 74.7%-81.3% across all training sets (**Supplementary Figure 32a-c** and **Supplementary Figure 33a,b**) and about 68% across the two test sets (**Supplementary Figure 32d,e** and **Supplementary Figure 33c,d**). In addition, we predicted the secondary structures of the AMP sequences in both the training and test sets. The 3D structures of the sequences were first predicted using PepFold4[12,13], and the residue-level secondary structures were then evaluated from the predicted 3D structures using DSSP (Dictionary of Protein Secondary Structure)[14,15]. We calculated the residue fractions assigned to different secondary structures for each sequence (**Supplementary Figure 34a** and **35a**) and classified an overall sequence as having a specific secondary structure when more than 50% of its residues were assigned to the same secondary-structure class; otherwise, it was classified as irregular. Most AMP sequences in both the training and test sets had α-helical structures, whereas about 15-20% had irregular

structures (**Supplementary Figure 34b and 35b**). Amphiphilicity and charge were also evaluated using the R package ampir[16] (**Supplementary Figure 34c, d** and **35c, d**).

1.2 Determination of the normalization approach for the training set of activity predictors

To determine whether normalization of activity values improves activity-prediction performance, we selected a published LSTM-based[17] model by Huang et al.[18] as the initial MIC regression model. We extracted the LSTM-seq regression model and dropped the previous XGBoost-based classification and ranking steps from their sequential-model-ensemble pipeline (SMEP) in which trainable 20-D amino-acid embeddings feed an LSTM and dense decoder. The default optimizer (Adam), default learning rate (0.002), and specified epoch number for convergence (150) are used.

We trained the model using 8,496 AMPs from DBAASP with or without normalized activity values based on different activity types, media, and strains. In addition, 93,408 non-AMPs were added to the training set, and 19,143 non-AMPs were added to the testing set. We assigned the highest activity value (13,112 μg/mL) to these sequences and used the augmented dataset for both training and testing. Despite the heterogeneity in experimental conditions (**Supplementary Figure 31a-c**), we found that the Pearson correlation and classifier precision/recall/F1 on the test set (195 AMPs with MICs and 19,143 augmented non-AMPs), using an MIC threshold of 100 μg/mL, were similar with or without normalization when 93,408 non-AMP sequences were included in training (**Supplementary Figure 36**). Therefore, we opted to use the unnormalized training dataset. This result also implies that we can pool all activity values from different activity

types (MIC and MBC) and treat them as MIC values (which correspond to MIC100 in our study) without materially affecting prediction performance.

1.3 Determination of the number of augmented non-AMPs in the training set of activity predictors

The number of augmented non-AMP sequences in the training set can influence the performance of antibacterial activity predictors. To assess this effect, we examined models trained with varying numbers of additional non-AMP sequences (0, ~5K, 10K, 15K, 30K, 60K, or 90K).

We observed that adding more non-AMP sequences consistently improved test performance, as reflected by higher Pearson correlation coefficients and increased precision at an MIC threshold of 100 μg/mL. This trend was consistent across the model by Huang et al.[18] and three additional models (APIN[19], AMPlify[20], and GrAMPA[21]). For APIN and AMPlify, which are binary classifiers, the sigmoid output activation was replaced with a linear function to convert them into regression models. We also evaluated an ensemble predictor that combined these four models by taking the maximum predicted activity values (**Supplementary Figure 37**).

The highest precision was achieved when 93,408 additional non-AMPs (~90K) were included in training. Because precision is more critical than recall for minimizing wasted effort in experimental validation, we selected the dataset containing 93,408 non-AMPs for subsequent model training. Under this condition, the ensemble predictor achieved a test precision of 0.95 (MIC threshold 100 μg/mL) when only sequences predicted as active by all four models were considered (**Supplementary Table 5**). Based on these results, we

incorporated 93,408 non-AMP sequences into the training of the activity predictors within our screening platform.

1.4 Toxicity Data

Toxicity data were used to evaluate the therapeutic index[22,23] and to assign sample weights during the second training iteration of the generative model in ARCADIAMP. Peptide toxicity measurements were curated from the DBAASP database (accessed February 15, 2024). The raw dataset contained 13,776 entries across various cell types.

We focused on hemolysis data, which accounted for 73% of the dataset, and standardized values to LC_{50} value (the peptide concentration causing 50% hemolysis), since this was the most commonly reported endpoint (22% of hemolysis entries). Data from different red blood cell sources were pooled. For hemolysis entries reported at 5% or 10% lysis (~20% of the data), we converted values to estimated LC_{50} using the median ratio of those doses relative to the 50% hemolysis dose (**Supplementary Table 10**). Entries reporting endpoints other than 5%, 10%, or 50% lysis were excluded due to insufficient data for reliable normalization.

After curation, 4,255 hemolysis measurements remained, covering 3,559 unique AMPs. We further excluded 220 AMPs that showed highly variable LC_{50} values, defined as a maximum-to-minimum LC_{50} ratio > 2. For each excluded AMP, we assigned a representative $Log_{10}(LC_{50})$ of 2.40, corresponding to the average of the remaining 3,339 AMPs.

2. Preliminary Models and Results

Before developing ARCADIAMP, we combined a published generative model, published activity predictors, and a conventional sequence novelty filter to screen AMPs. This preliminary framework (**Algorithm 1**) yielded moderate results but provided the foundation for ARCADIAMP by highlighting areas for modification and improvement.

Algorithm 1. Initial Screening Pipeline

Inputs: $\{L_{\text{Seq}}, n, \mathbb{S}_{\text{AMP}}, T\}$

- L_{Seq} : Target AMP candidate length.
- n : Number of AMP candidate to generate.
- $\mathbb{S}_{\text{AMP}}$: Set of well-known AMPs curated from databases
- T: Thresholds $\{T_{\text{MIC}}, T_S, T_{\text{ID}}\}$, where T_{MIC} is MIC cutoff (e.g., 100 μg/mL), T_S is similarity cutoff ($\in (0,1)$) (e.g., 0.45), and T_{ID} is the identity cutoff ($\in (0,1)$) (e.g., 0.65)

Output: $\mathbb{S}_C$: Screened strong, novel AMP candidates

$\mathbb{S}_{\text{GEN}} \leftarrow \mathcal{G}_1(L_{\text{Seq}}, n)$	//Generate candidates with diffusion model $\mathcal{G}_1$ (see section 2.1, 3.1)
$\mathbb{S}_{\text{ACT}} \leftarrow \{\text{seq} \in \mathbb{S}_{\text{GEN}} \mid \mathcal{R}(\text{seq}) < T_{\text{MIC}}\}$	//Select candidates with strong predicted activity by ensemble model $\mathcal{R}$
$(\boldsymbol{V}_S, \boldsymbol{V}_{\text{ID}}) \leftarrow \text{novelty\_eval}(\mathbb{S}_{\text{ACT}}, \mathbb{S}_{\text{AMP}})$	//Evaluate similarity and identity vs. known AMPs (see section 2.3)
$\mathbb{S}_C \leftarrow \{\text{seq} \in \mathbb{S}_{\text{ACT}} \mid V_S^{\text{seq}} < T_S \wedge V_{\text{ID}}^{\text{seq}} < T_{\text{ID}}\}$	//Select candidates that pass novelty thresholds
**return** $\mathbb{S}_C$	

2.1 EvoDiff-D3PM-based Generative Model

We adapted EvoDiff-D3PM[24] — a recently published protein language foundation model based on discrete diffusion (D3PM)[25] (**Supplementary Figure 2a**).

The corruption process in EvoDiff-D3PM gradually perturbs the input training sequence vector ($\boldsymbol{x}_0$) across T timesteps ($T = 500$ in our case). Each vector contains scalar random variables with 26 categories (20 standard L-form amino acids and 6 non-standard residues; **Supplementary Figure 2b**):

$$q(\boldsymbol{x}_t|\boldsymbol{x}_{t-1}) = \text{Categorical Distribution}(\boldsymbol{x}_t; \boldsymbol{p} = \boldsymbol{x}_{t-1}\boldsymbol{Q}_t) \quad (1)$$

Where $\boldsymbol{Q}_t$ is the transition matrix of the Markov process[26], defined as:

$$\boldsymbol{Q}_{t_{ij}} = \begin{cases} (1-\beta_t) + \beta_t/26 & for\ i = j\ (Not\ Mutated) \\ \beta_t/26 & for\ i\ \neq j\ (Mutated) \end{cases} \tag{2}$$

The β_t value varies with each timestep (noise scheduling) according to Sohl-Dickstein et al.[27]:

$$\beta_t = \ (T - t + 1)^{-1} \tag{3}$$

The chained transition matrix and reverse corruption step (one of the required items for evaluating the loss of the generative model later) are then defined as:

$$\overline{\boldsymbol{Q}_t} = \boldsymbol{Q}_1 \boldsymbol{Q}_2 \dots \boldsymbol{Q}_t \tag{4}$$

$$q(\boldsymbol{x}_{t-1}|\boldsymbol{x}_t,\ \boldsymbol{x}_0) = \text{Categorical Distribution}\left(\boldsymbol{x}_{t-1}; \boldsymbol{p} = \frac{\boldsymbol{x}_t \overline{\boldsymbol{Q}_t} \odot \boldsymbol{x}_0 \overline{\boldsymbol{Q}_{t-1}}}{\boldsymbol{x}_0 \overline{\boldsymbol{Q}_t} \boldsymbol{x}_t}\right) \tag{5}$$

The neural network predicts the denoising step $p_\theta(\boldsymbol{x}_{t-1}|\boldsymbol{x}_t)$ and is trained with a hybrid loss function[24]:

$$\mathcal{L}_\lambda = \mathcal{L}_{vb} + \lambda \mathcal{L}_{ce} \tag{6}$$

$$\mathcal{L}_{vb} = \mathbb{E}_{q(\boldsymbol{x}_0)}\left[\underbrace{D_{KL}[q(\boldsymbol{x}_T|\boldsymbol{x}_0)||p(\boldsymbol{x}_T)]}_{\mathcal{L}_T} + \sum_{t=2}^{T} \underbrace{\mathbb{E}_{q(\boldsymbol{x}_t|\boldsymbol{x}_0)}\left[D_{KL}[q(\boldsymbol{x}_{t-1}|\boldsymbol{x}_t, \boldsymbol{x}_0)||p_\theta(\boldsymbol{x}_{t-1}|\boldsymbol{x}_t)]\right]}_{\mathcal{L}_{t-1}} - \underbrace{\mathbb{E}_{q(\boldsymbol{x}_1|\boldsymbol{x}_0)}[\log p_\theta(\boldsymbol{x}_0|\boldsymbol{x}_1)]}_{\mathcal{L}_0}\right] \tag{7}$$

The original D3PM formulation predicted the entire denoising process $\widetilde{p_\theta}(\widetilde{\boldsymbol{x}_0}|\boldsymbol{x}_t)$, rather than a single step $p_\theta(\boldsymbol{x}_{t-1}|\boldsymbol{x}_t)$, following Ho et al.[28] and Hoogeboom et al.[29], with the two being proportional:

$$p_\theta(\boldsymbol{x}_{t-1}|\boldsymbol{x}_t) \propto \sum_{\widetilde{\boldsymbol{x}_0}} q(\boldsymbol{x}_{t-1}|\boldsymbol{x}_t, \widetilde{\boldsymbol{x}_0})\widetilde{p_\theta}(\widetilde{\boldsymbol{x}_0}|\boldsymbol{x}_t) \quad (8)$$

Based on this, inference with *k* steps $p_\theta(\boldsymbol{x}_{t-k}|\boldsymbol{x}_t)$ can be performed simultaneously :

$$p_\theta(\boldsymbol{x}_{t-k}|\boldsymbol{x}_t) = \sum q(\boldsymbol{x}_{t-k}|\boldsymbol{x}_t, \widetilde{\boldsymbol{x}_0})\widetilde{p_\theta}(\widetilde{\boldsymbol{x}_0}|\boldsymbol{x}_t) \quad (9)$$

An auxiliary cross-entropy loss $\mathcal{L}_{ce}$ was also introduced in D3PM and adapted for EvoDiff-D3PM:

$$\mathcal{L}_{ce} = \mathbb{E}_{q(x_0)}\mathbb{E}_{q(x_t|x_0)}[-\log \widetilde{p_\theta}(\boldsymbol{x}_0|\boldsymbol{x}_t)] \quad (10)$$

The neural network $\widetilde{p_\theta}(\widetilde{\boldsymbol{x}_0}|\boldsymbol{x}_t)$ was implemented using stacked ByteNet[30] Blocks with specified dimensions (**Supplementary Figure 2c**). We used a pretrained EvoDiff-D3PM model (38M parameters, uniform perturbation schedule, trained on ~42 million UniRef50[31] sequences). For hyperparameter optimization, we tested multiple diffusion timesteps $T = \{100,200,300,400,500\}$ and auxiliary cross-entropy loss weights $\lambda = \{0, 0.001, 0.002, 0.005, 0.01, 0.02, 0.04, 1\}$. All tests were conducted using the smaller 38M-parameter model for efficiency, as the larger 640M-parameter model would require excessive inference times and its performance was not significantly better (data not shown). Each model was trained for up to 500 epochs, with checkpoints saved at 100, 200, 300, and 400 epochs to monitor convergence and performance.

2.2 Ensemble MIC Regression Model

We selected four published MIC predictors for ensemble modeling: Huang et al.[18], APIN[19], AMPlify[20], and GrAMPA[21] (**Supplementary Figure 37**). From Huang et al. [18] we extracted the LSTM-seq regression model, omitting the original XGBoost-based classification and ranking steps. APIN[19] retains its 128-D residue-embedding layer and 14 1-D CNN and max-pooling layers. AMPlify[20] ingests one-hot vectors (20 × L - length of the sequence)

into a 512-unit Bi-LSTM, with multi-head scaled-dot-product and context attention layers. GRAMPA[21] uses two 64-filter 1-D convolutional layers on one-hot encodings, followed by max-pooling and dense layers. APIN and AMPlify were converted from classification to regression by replacing the final sigmoid layer with a linear neuron. Training used default optimizers (Adam, except RMSProp for APIN), default learning rates (0.002 for Huang et al., 0.0002 for APIN, and 0.001 for Amplify and Grampa), and epoch number for convergence (150 for Huang et al., 300 for Amplify, 1,000 for APIN and Grampa).

When trained with 93,408 augmented non-AMPs, model precisions (MIC < 100 μg/mL) ranged from 0.69 to 0.88. The ensemble, which took the maximum predicted MIC from the four models, achieved a precision of 0.95 (**Supplementary Table 5**). These results confirmed that ensemble regression effectively screens AMPs at an MIC threshold of 100 μg/mL.

2.3 Sequence Novelty Filter

To evaluate sequence novelty, candidate AMPs were aligned against 27,636 known AMPs collected from five databases (DBAASP, CAMPR4, DBAMP, DRAMP, and APD; **Supplementary Figure 4**, **Supplementary Table 6**). Alignments were performed using the Needleman-Wunsch global algorithm[32] with BLOSUM62[33] and gap penalties of −11 (open), −1 (extension), and 0 (terminal).

Candidates were considered non-novel if they contained a subsequence of a known AMP or were themselves subsequences of known AMPs. Both cases can yield artificially low similarity due to terminal gaps. To address this, we used sequence identity (percentage of identical residues in the aligned region) in addition to normalized similarity scores. Because terminal gaps are excluded, subsequence relationships produce high identity

despite low similarity. We defined AMP candidates as novel if their normalized alignment score < 0.45 and its identity < 0.65 versus the closest known AMP.

2.4 Initial Screening Results

Using the generative model, ensemble MIC predictors, and novelty filter, we generated 112,995 candidate sequences. Of these, 13,385 were predicted by all four models to have MIC < 100 μg/mL. After excluding sequences with > 2 cysteine residues (to avoid complications in synthesis), 13,038 remained. Applying the novelty filter reduced this to 741 candidates.

From these, ten peptides were selected for synthesis and validation, prioritized by lowest predicted MIC and highest novelty. Their predicted MICs, experimental MICs (in MHB and CaMHB), and hemolytic activities (LC_{50}) are summarized in **Supplementary Table 11**.

Seven of the ten candidates had measured MICs < 100 μg/mL in MHB, and three retained MICs < 100 μg/mL in CaMHB. These findings demonstrate that a diffusion-based generator combined with ensemble MIC regression model can systematically identify novel AMPs with therapeutic indices comparable to prior studies. However, all candidates exhibited MIC values above 10 μg/mL, indicating lower potency than the strongest known AMPs or the FDA-approved peptide colistin. This result motivated the development of an improved pipeline to specifically target highly potent AMPs (MIC < 10 μg/mL).

3. ARCADIAMP Platform

To identify strong AMPs with low MICs, we modified the preliminary framework (**Algorithm 2**). The EvoDiff-D3PM-based generative model retained the same architecture but was retrained with augmented data and sample-dependent cross-entropy weights (λ). For activity prediction, we implemented a two-stage ESM2-based[34] antibacterial activity classifier to predict the activity of the AMP candidates: the first stage classified AMPs versus non-AMPs, and the second classified strong AMPs (MIC < 8 μg/mL). The conventional sequence novelty filter was retained without modification (**Supplementary Figure 4**).

Algorithm 2. ARCADIAMP Screening Pipeline

Inputs: $\{L_{\text{Seq}}, n, \mathbb{S}_{\text{AMP}}, T\}$

- L_{Seq} : Target AMP candidate length
- n : Number of AMP candidate to generate
- $\mathbb{S}_{\text{AMP}}$: Set of well-known AMPs curated from databases
- T: Thresholds $\{T_S, T_{\text{ID}}\}$, where T_S is similarity cutoff ($\in (0,1)$) (e.g., 0.45) and T_{ID} is identity cutoff ($\in (0,1)$) (e.g., 0.65)

Output: $\mathbb{S}_C$: Screened strong, novel AMP candidates.

1: $\mathbb{S}_{\text{GEN}} \leftarrow \mathcal{G}_2(L_{\text{Seq}}, n)$ //Generate candidates with 2nd round trained $\mathcal{G}_2$ (see section 3.1)

2: $\mathbb{S}_{\text{ACT}} \leftarrow \{\text{seq} \in \mathbb{S}_{\text{GEN}} \mid \mathcal{C}_{\text{Coarse}}(\text{seq}) = \textbf{AMP}\}$ //Coarse-grained screen : AMP vs non-AMP (see section 3.2)

3: $\mathbb{S}_{\text{STRONG}} \leftarrow \{\text{seq} \in \mathbb{S}_{\text{ACT}} \mid \mathcal{C}_{\text{Fine}}(\text{seq}) = \textbf{Strong}\}$ //Fine-grained screen: AMP with MIC < 8 μg/mL (see section 3.2)

4: $(\boldsymbol{V_S}, \boldsymbol{V_{\text{ID}}}) \leftarrow \text{novelty\_eval}(\mathbb{S}_{\text{STRONG}}, \mathbb{S}_{\text{AMP}})$ //Evaluate similarity and identity vs. known AMPs (see section 2.3)

5: $\mathbb{S}_C \leftarrow \{\text{seq} \in \mathbb{S}_{\text{STRONG}} \mid V_{S,\text{Seq}} < T_S \wedge V_{\text{ID,Seq}} < T_{\text{ID}}\}$ //Select candidates that pass novelty thresholds

6: **return** $\mathbb{S}_C$

3.1 Iterative-Learning EvoDiff-D3PM-based Generative Model in ARCADIAMP

The EvoDiff-D3PM-based generative model in ARCADIAMP can be trained iteratively in conjunction with an MIC predictor. In each iteration, an augmented training set is created by selecting generated sequences with low predicted MIC values. This augmented set is

then combined with the existing training set to form the data for the next iteration. Additionally, sample-dependent cross-entropy weighting can be applied during training (**Algorithm 3**). In ARCADIAMP, the EvoDiff-D3PM-based generative model was trained for two iterations. After the first iteration, 16,334 sequences generated by the preliminary model with predicted MIC < 32 μg/mL (using the model by Huang et al.[18]) were selected as augmented training data (**Supplementary Figure 2d,e**). In the second iteration, sample-dependent cross-entropy weighting was also applied during training (**Supplementary Figure 7**).

Algorithm 3. Iterative Learning of the EvoDiff-D3PM-based Generative Model

---

Inputs: $\{\mathbb{D}_T, \mathcal{G}_0, k, \mathrm{Op}_\lambda, \mathrm{Par}_{\mathrm{Gen}}\}$

- $\mathbb{D}_T = \{\mathbb{S}_T, \boldsymbol{M}_A, \boldsymbol{V}_T\}$: Training dataset. $\mathbb{S}_T$: Curated AMP sequences, $\boldsymbol{M}_A$: the activity matrix (LogMIC values), and $\boldsymbol{V}_T$: the toxicity vector ($LogLC_{50}$ values)
- $\mathcal{G}_0$: The initial EvoDiff-D3PM model (38M-parameter, Uniform)
- k: Number of training iterations
- Op_λ**:** Option: sample-wise cross-entropy weighting (Algorithm 4). If False, used fixed $\lambda = 0.02$ for all samples.
- $\mathrm{Par}_{\mathrm{Gen}}$: Parameters for generation (sequence length, number)

Output: $\mathcal{G}_k$, the generative model after k training iterations

$\mathbb{D}_T^{(1)} \leftarrow \mathbb{D}_T$	//Initial training set
**for** i in 1 : k **do**	
$\mathcal{G}_i \leftarrow Train(\mathcal{G}_0, \mathbb{D}_T^{(i)}, \mathrm{Op}_\lambda^{(i)})$	//Training (We always train $\mathcal{G}_0$ in our case)
**if** i < k **then**	
$\mathbb{S}_{\mathrm{Aug}} \leftarrow \mathcal{G}_k(\mathrm{Par}_{\mathrm{Gen}})$	//Generate the augmented sequences
$\mathbb{S}_{\mathrm{Aug}} \leftarrow \{Seq \in \mathbb{S}_{\mathrm{Aug}} \mid \mathcal{R}(\mathrm{Seq}) < 32\}$	//Select sequences with predicted MIC < 32 μg/mL by regression model $\mathcal{R}$
$\mathbb{D}_T^{(i)} \leftarrow$ extend_training_set($\mathbb{D}_T^{(i)}, \mathbb{S}_{\mathrm{Aug}}$)	//Extend the training set.
**end if**	
**end for**	
**return** $\mathcal{G}_k$	

To compute λ, we collected Log_{10} (MIC) values for six ESKAPE species and Log_{10} (LC_{50}) values for toxicity. Missing Log_{10} (MIC) and Log_{10} (LC_{50}) were imputed using average values of 2.00 and 2.40, respectively. The therapeutic index (LC_{50}/MIC) was then calculated, and a piecewise function mapped the therapeutic index to raw cross-entropy weights (λ_{Raw}). Because each sequence had six λ_{Raw} values (one per species), we aggregated them by evaluating the product of them with normalization and applying the lower bound of 0.001 to obtain the final λ (**Algorithm 4**).

Algorithm 4. Samplewise cross-entropy weight (λ) evaluation

Inputs: $\{\boldsymbol{V_A}, v_t\}$

- $\boldsymbol{V_A}$:Vector of Log_{10}MIC values across six ESKAPE species
- v_t : $Log_{10}LC_{50}$ value targeting human red blood cells

Output: λ_{Seq}: samplewise cross-entropy weight for training

```
(V_A, v_t) ← fill_NA(V_A, v_t, 2.0, 2.4)        //Fill missing Log10MIC and Log10LC50 with empirical averages = (2.0, 2.4)
V_TI ← v_t · 1 − V_A                            //Calculate the Log therapeutic index (TI) for each species
v_Base ← Log10(256/100)                         //Define the base Log therapeutic index (≈ 0.41)
for i in 1 : 6 do                               //Assign the raw weight per species
    if V_TI^i ≥ 1 then
        λ_Raw^i ← min(V_TI^i + 1, 3)            //High Log TI
    elseif V_TI^i ∈ [v_Base, 1) then
        λ_Raw^i ← 1 + ((V_TI^i − v_Base)/(1 − v_Base))   //Medium Log TI
    else
        λ_Raw^i ← max((0.9 V_TI^i)/v_Base, 0.1)  //Low Log TI
    end if
end for
λ_Aggr ← ∏_i λ_Raw^i ∀ i                        //Aggregate across species
λ_Seq ← λ_Aggr/8.63                             //Normalize by constant (= 8.63, the average value of all sequences)
λ_Seq ← max(0.001, λ_Seq)                       //Applying the lower bound of 0.001
return λ_Seq
```

The EvoDiff-D3PM model was retrained using these sample-dependent weights with the same hyperparameters applied in the first training iteration. We found that the algorithm for determining the sample training weights may prefer specific secondary structure of the peptide – by comparing the sample weights of the sequences with different predicted secondary structure, we found that the sequences with β-strand have the highest sample weights, and the sequences with irregular structure have significantly lower sample weights compared to other structures (**Supplementary Figure 38a**). However, we found no significant correlation between the sample weights and other properties such as amphiphilicity and charge (**Supplementary Figure 38b, c**).

3.2 Two-Stage ESM2-based Activity Classifier

To improve the identification of very strong AMPs (MIC < 8 μg/mL), we implemented a two-stage ESM2-based antibacterial activity classifier. The first classifier (coarse-grained) distinguished AMPs from non-AMPs, while the second (fine-grained) identified strong AMPs (MIC < 8 μg/mL). Both were based on pretrained ESM-2 models[34] (MetaAI) which were obtained via the official GitHub repository, fine-tuned on different training sets (**Fig. 1b**). Final predictions were generated by averaging probabilities from 5-fold cross-validation models.

We evaluated ESM-2 models of varying sizes (8M, 35M, 150M, 650M parameters) under different learning rates, dropout rates, and weight decay values. Larger models did not yield significant performance gains, while the 8M model converged quickly, tolerated dropout up to 0.7, and demonstrated strong generalization. Consequently, we selected the 8M model for subsequent experiments as it provided the best balance of performance and computational efficiency (**Supplementary Figure 3**).

Models were implemented in Python with PyTorch and trained on the Expanse supercomputing cluster (SDSC) using NVIDIA V100 GPUs (32 GB memory). Sample weights for both classifiers were tuned via parameter search to prioritize strong AMPs and penalize weak AMPs (**Supplementary Figure 11**). We also found that the sample weights for training the coarse-grained classifier and fine-grained classifier of the sequences with different secondary structure were significantly different. For the sample weights for training the coarse-grained model, the sequences with predicted β-strand structure have the highest weights, and the sequences with predicted α-helix structure have the lowest weights; for the sample weights for training the fine-grained model, the sequences with predicted β-strand structure have significantly higher ratio (15.5%) of the strong AMP (MIC much less than 8 such that the sample weight is higher than 12). However, we found no significant correlation between the sample weights for training the classifiers and other properties such as amphiphilicity and charge (**Supplementary Figure 39, 40**).

3.3 Evaluation of the Generative Model

We compared predicted MICs (based on the model by Huang et al.[18]) and sequence novelty (identity and similarity) for sequences generated by different models. For most comparisons, 30,000 sequences were generated, and the peptide length distribution followed that of the training set. For several test cases, larger numbers of sequences were generated, and we found no significant differences in the average and median predicted MICs across different generation sizes (122, 520, 1019, and 3021). In addition, the average similarity and identity converged to 0.50 and 0.57, respectively, as the number of generated sequences increased (**Supplementary Figure 41**). Based on these results,

we expected that variance in generated sequence number would not affect the claims or analysis when the number of generated sequences was substantially greater than 3,000. First, to justify the selection of the D3PM-based model, we compared our model - trained from randomly initialized weights and without applying cross-entropy loss - against the OADM-based model, fine-tuned models based on pretrained models, and models trained without cross-entropy loss. Second, to demonstrate the advantages of iterative training, the model trained with augmented sequences was compared with the model trained only on the initial training sequences. Two negative-control models (the pretrained D3PM model without AMP fine-tuning and a random-sequence generator) were also included. Third, we compared our model with other published peptide generative models, including BroadAMP-GPT[35] (GPT-based) and Pep-Diffusion[36] (Diffusion-based). Finally, we evaluated the performance of models trained with augmented training sequences generated across different numbers of training iterations.

3.4 Evaluation of the Activity Classifiers

We also benchmarked our coarse-grained activity classifier against three published AMP classification methods: AMPlify[20], PepNet[38], and Diff-AMP[39]. To ensure a fair comparison, AMPs present in the training sets of these models were removed from our test set. AMPlify is based on a multi-head attention architecture, Diff-AMP uses a multilayer perceptron, and PepNet employs a transformer-based model. We downloaded the available pretrained models and datasets for these methods. For AMPlify, we evaluated two versions of the model: one trained on a balanced dataset (3,338 AMPs and 3,338 non-AMPs) and one trained on an imbalanced dataset (3,338 AMPs and 102,756 non-AMPs). For Diff-AMP, we used the published model trained on 3,307 AMPs and 3,307

non-AMPs. For PepNet, we used the model trained on 8,865 AMPs and 7,863 non-AMPs; we tested both the “simple” version of this model and the “complete” version that incorporates additional features from ProtTrans[40]. In total, five comparison models (two versions of AMPlify, one Diff-AMP, and two versions of PepNet) were evaluated against our ESM-2 coarse-grained classifier. To assess the significance of the improvement of the ESM-2 coarse-grained classifier, we applied the bootstrap approach[41] by randomly sampling 20% of the test dataset and evaluated recall, precision, F1, and balanced accuracy at the F1-optimized operating point across 100 iterations. A one-tailed *t*-test was then applied to evaluate whether the coarse-grained classifier achieved significantly better performance.

In addition to the coarse-grained model, the binary-classification benchmarks (recall, precision, F1, and balanced accuracy) of the fine-grained model were compared against those of the coarse-grained classifier and the ensemble MIC regression model (the best-performing model for distinguishing AMPs from non-AMPs) to assess their ability to screen strong AMPs (MIC < 8 μg/mL). The same bootstrap approach and statistical test were applied to determine whether the fine-grained classifier achieved significantly better performance.

Supplementary Tables

Supplementary Table 1. Performance comparison of generative models trained with different numbers of iterations. Bold values indicate the best performance.

#Iterations	#Training Sequences	Performance (Median with IQR)		
		Predicted MIC (μg/mL)	Similarity	Identity
1	13,950	53 (11 – 2877)	0.67 (0.51 – 0.85)	0.72 (0.58 – 0.87)
2	26,244	37 (10 – 352)	0.51 (0.40 – 0.65)	0.55 (0.46 – 0.69)
3	50,832	**27 (7 – 172)**	0.47 (0.37 – 0.58)	0.53 (0.44 – 0.61)
4	96,029	27 (7 – 196)	0.45 (0.36 – 0.55)	0.50 (0.44 - 0.60)
5	190,402	28 (7 – 192)	**0.43 (0.35 - 0.53)**	**0.5 (0.43 – 0.57)**

Supplementary Table 2. Performance comparison of classifiers distinguishing AMPs from non-AMPs in the test set (N_{AMP} = 195, $N_{Non\text{-}AMP}$ = 19,143). For fair comparison, thresholds were adjusted to maximize each model's F1-score (MIC cutoff for ensemble models; probability cutoff for classifiers). Model names indicate the number of negative samples used in training and whether instance weighting was applied.

Model (Training Data or Method)		Classification Performance (N_{AMP} = 195, $N_{Non\text{-}AMP}$ = 19,143)				
		F1-Score	Precision	Recall (Sensitivity)	Balanced Accuracy	Specificity
Coarse-grained Classifier	(Weighted)	0.8587	0.9337	0.7949	0.8971	0.9994
	(Equal Weights)	0.8635	0.9451	0.7949	0.8972	0.9995
Amplify[20]	(Balanced Training set)	0.5382	0.6616	0.4536	0.7256	0.9976
	(Imbalanced Training set)	0.2924	0.2094	0.4845	0.7330	0.9815
PepNet[38]	(Without Structural Features)	0.4908	0.5054	0.4769	0.7361	0.9952
	(With Structural Features)	0.5077	0.4427	0.5949	0.7936	0.9924
DiffAMP[39]		0.6979	0.8150	0.6103	0.8044	0.9986
The Ensemble Model		0.8131	0.8757	0.7590	0.8789	0.9989
Model by Huang et al.[18]	(in the Ensemble Model)	0.6969	0.7785	0.6308	0.8145	0.9982
Amplify[20]	(in the Ensemble Model)	0.7525	0.7273	0.7795	0.8883	0.9970
APIN[19]	(in the Ensemble Model)	0.7500	0.8408	0.6770	0.8378	0.9987
Grampa[21]	(in the Ensemble Model)	0.7278	0.7939	0.6718	0.8350	0.9982

Supplementary Table 3. Antibacterial activity of AMP candidates, expressed as minimum inhibitory concentrations (MICs), measured in Mueller-Hinton broth (MHB) and cation-adjusted MHB (CaMHB).

Peptide	Activity (MIC) (µg/mL)											
	E. faecalis ATCC 29212		*S. aureus* ATCC 25923		*K. pneumonia* ATCC 700603		*A. baumannii* ATCC 19606		*P. aeruginosa* ATCC 27853		*E. coli* ATCC 25922	
	MHB	CaMHB	MHB	CaMHB	MHB	CaMHB	MHB	CaMHB	MHB	CaMHB	MHB	CaMHB
Peptide-1	128	>128	32	>128	32	>128	32	>128	16	>128	64	>128
Peptide-2	>128	>128	16	64	8	8	8	8	8	32	8	8
Peptide-3	64	>128	32	128	32	128	32	128	32	128	32	64
Peptide-4	64	128	32	128	32	128	32	64	32	128	32	32
Peptide-5	128	>128	32	128	32	128	32	128	16	>128	32	64
Peptide-6	64	>128	16	128	32	128	16	32	16	>128	16	32
Peptide-7 (Arcinin)	64	>128	16	32	8	16	8	16	8	32	8	16
Peptide-8	64	>128	32	128	32	128	32	>128	32	>128	32	64
Peptide-9	>128	>128	64	>128	>128	>128	>128	>128	>128	>128	64	>128
Peptide-10	>128	>128	32	128	128	>128	16	64	8	128	32	64
Pexiganan	32	128	16	32	8	16	8	8	8	16	8	16
Hydraganan-1	32	>128	16	16	16	>128	4	8	16	>128	4	8
Melittin	8	4	8	4	32	128	8	4	32	128	8	16
Colistin	>128	>128	>128	>128	2	1	1	0.5	2	1	1	2
Daptomycin	16	2	32	2	>128	>128	>128	>128	>128	>128	>128	>128

Supplementary Table 4. *In vitro* serum stability assessment of AMP candidates, reported as minimum inhibitory concentrations (MICs) measured in 50% bovine serum.

Peptide	*In vitro* serum stability (MIC) (μg/mL)					
	E. faecalis ATCC 29212	*S. aureus* ATCC 25923	*K. pneumonia* ATCC 700603	*A. baumannii* ATCC 19606	*P. aeruginosa* ATCC 27853	*E. coli* ATCC 25922
Peptide-1	>128	>128	>128	>128	>128	>128
Peptide-2	>128	64	32	64	>128	128
Peptide-3	>128	64	128	32	>128	128
Peptide-4	>128	128	>128	64	>128	128
Peptide-5	>128	32	>128	>128	>128	>128
Peptide-6	>128	32	>128	32	>128	128
Peptide-7 (Arcinin)	>128	32	32	32	>128	32
Peptide-8	>128	128	>128	>128	>128	>128
Peptide-9	>128	>128	>128	>128	>128	>128
Peptide-10	>128	>128	>128	>128	>128	>128
Pexiganan	128	8	4	8	64	64
Hydraganan-1	>128	32	>128	>128	>128	>128
Melittin	32	32	>128	64	>128	>128
Colistin	>128	>128	1	0.5	1	2
Daptomycin	2	0.5	>128	>128	>128	>128

Supplementary Table 5. Binary classification performance (MIC threshold < 100 μg/mL) of four models and the ensemble predictor (max MIC from four models) trained with varying numbers of additional non-AMPs. Bold values indicate the best performance.

Models	#Non-AMPs added (Label)	TP	TN	FP	FN	Recall	Precision	F1
Huang et al.	93,408 (90K)	86	19,159	39	54	0.61	0.69	0.65
	62,276 (60K)	84	19,147	51	56	0.6	0.62	0.61
	31,137 (30K)	91	19,084	114	49	0.65	0.44	0.53
	15,570 (15K)	93	19,006	192	47	0.66	0.33	0.44
	10,380 (10K)	85	18,976	222	55	0.61	0.28	0.38
	5,190 (5K)	87	18,861	337	53	0.62	0.21	0.31
	0 (0K)	93	15,375	3,823	47	0.66	0.02	0.05
Amplify	93,408 (90K)	61	19,186	12	79	0.44	0.84	0.57
	62,276 (60K)	79	19,171	27	61	0.56	0.75	0.64
	31,137 (30K)	74	19,180	18	66	0.53	0.8	0.64
	15,570 (15K)	75	19,177	21	65	0.54	0.78	0.64
	10,380 (10K)	82	19,140	58	58	0.59	0.59	0.59
	5,190 (5K)	97	19,096	102	43	0.69	0.49	0.57
	0 (0K)	93	14,303	4,895	47	0.66	0.02	0.04
APIN	93,408 (90K)	64	19,189	9	76	0.46	0.88	0.6
	62,276 (60K)	60	19,187	11	80	0.43	0.85	0.57
	31,137 (30K)	73	19,182	16	67	0.52	0.82	0.64
	15,570 (15K)	70	19,185	13	70	0.5	0.84	0.63
	10,380 (10K)	79	19,175	23	61	0.56	0.77	0.65
	5,190 (5K)	64	19,190	8	76	0.46	0.89	0.6
	0 (0K)	106	16,988	2,210	34	0.76	0.05	0.09
Grampa	93,408 (90K)	89	19,161	37	51	0.64	0.71	0.67
	62,276 (60K)	88	19,158	40	52	0.63	0.69	0.66
	31,137 (30K)	93	19,156	42	47	0.66	0.69	0.68
	15,570 (15K)	92	19,116	82	48	0.66	0.53	0.59
	10,380 (10K)	91	19,101	97	49	0.65	0.48	0.55
	5,190 (5K)	94	18,999	199	46	0.67	0.32	0.43
	0 (0K)	107	15,795	3,403	33	0.76	0.03	0.06
Max of Four Models	93,408 (90K)	38	19196	**2**	102	0.27	**0.95**	0.42

Supplementary Table 6. Number of AMP sequences composed of the 20 standard amino acids across five AMP databases, along with additional extracted metadata fields. The union of these datasets was considered the well-known AMP set and used to evaluate the sequence novelty of screened AMP candidates.

Database	# AMP Sequences	Extracted metadata fields in addition to the sequence
DBAASP	13,563	• DBAASPID<br>• Number of disulfide bonds<br>• Antibacterial Activity Entries<br>- Target Species and Strain<br>- Activity values with type (MIC or MBC), values, and units<br>- Medium<br>- CFU<br>- Note<br>- Reference (PMID)<br>• Toxicity Entries<br>- Target cells<br>- Percentage of the cell death or lysis<br>- The concentration applied (with units)
CAMPR4	19,933	• CAMPR4ID<br>• Antibacterial Activity Entries<br>- Target Species and Strain<br>- Activity values with type (MIC or MBC), values, and units<br>- Medium (Incomplete)<br>- Reference (PMID)
DBAMP<br>DRAMP<br>APD	11,674<br>7,451<br>3,179	None (the sequences are only used for AMP candidates sequence novelty evaluation)
(Union)	27,636	

Supplementary Table 7. Minority *E. coli* strains with average activity value ratios significantly greater than or less than 1 ($p < 0.05$) from the majority strain (ATCC 25922). Ratios greater than 1 indicate higher activity values, and ratios less than 1 indicate lower activity values relative to the reference strain.

Strain	Number of activity values	Compared with the majority strain (ATCC 25922) (Number of activity values = 7,592)		
		Number of values for comparison	Ratio of Average Activity Values	p-value (Two-sided t-test)
BW25113 del-sbmA	75	45	9.37	$3.85*10^{-4}$
CVCC 1515	45	19	4.68	$4.04*10^{-3}$
O157:H7	91	15	3.80	$2.76*10^{-2}$
RKI 6A-6	9	7	3.11	$8.32*10^{-3}$
(Clinical Isolate)	1,498	293	3.11	$1.60*10^{-6}$
UB1005	709	412	1.75	$5.30*10^{-4}$
O18K1H7	11	11	1.73	$2.36*10^{-2}$
K-12	281	26	1.68	$4.65*10^{-2}$
DH5alpha	422	40	1.47	$6.12*10^{-3}$
ATCC 11775	131	33	1.45	$2.18*10^{-2}$
1057	23	19	1.42	$5.56*10^{-3}$
BW25113	184	62	1.37	$1.36*10^{-2}$
ATCC 43827	25	19	0.72	$2.94*10^{-3}$
HB101	49	17	0.72	$2.24*10^{-3}$
B2	34	17	0.56	$7.45*10^{-4}$
CCT 1457	14	9	0.47	$4.25*10^{-3}$
EC183	10	8	0.33	$4.12*10^{-6}$
CGSC 5167	9	9	0.27	$2.26*10^{-6}$

Supplementary Table 8. Minority media with average activity value ratios significantly greater than or less than 1 ($p < 0.05$) from the majority medium (Mueller-Hinton broth, MHB) in the *E. coli* dataset. Ratios greater than 1 indicate higher activity values, and ratios less than 1 indicate lower activity values relative to MHB.

Strain	Number of activity values	Compared with the majority medium (MHB) (Number of activity values = 10,336)		
		Number of values for comparison	Ratio of Average Activity Values	p-value (Two-sided t-test)
M9	66	7	12.76	$2.25*10^{-2}$
CAMHB	1,006	37	3.48	$4.01*10^{-2}$
NB	313	14	0.60	$2.16*10^{-3}$
LYM	60	6	0.44	$2.56*10^{-2}$

Supplementary Table 9. Minority activity types with average activity value ratios significantly greater than or less than 1 ($p < 0.05$) from the majority activity type (MIC_{100}) in the *E. coli* dataset. Ratios greater than 1 indicate higher activity values, and ratios less than 1 indicate lower activity values relative to MIC_{100}.

Strain	Number of activity values	Compared with the majority activity type (MIC_{100}) (Number of activity values = 15,316)		
		Number of values for comparison	Ratio of Average Activity Values	p-value (Two-sided t-test)
MBC	2,173	106	1.89	1.08×10^{-5}
MIC_{50}	180	12	0.45	2.72×10^{-4}

Supplementary Table 10. Minority hemolysis ratios with median toxic dose ratios significantly greater than or less than 1 ($p < 0.05$) from the majority hemolysis ratio (50%). Ratios greater than 1 indicate higher toxic doses relative to the 50% reference.

Minority Hemolysis Ratio	Number of measurements	Compared with the majority hemolysis ratio (50%) (Number of measurements = 2,327)		
		Number of toxic measurements for comparison	Ratio of Median Activity Values	p-value (Two-sided t-test)
5%	827	11	4.00	$4.36*10^{-2}$
10%	1,098	72	3.01	$1.06*10^{-5}$
15%	102	11	4.00	$5.72*10^{-5}$
18%	76	9	4.00	$3.35*10^{-3}$

Supplementary Table 11. Measured MIC and LC_{50} values of the ten AMP candidates from initial screening, along with reference AMPs.

AMP Candidates and Published AMP		Predicted Median MIC (ug/ml)	Measured MIC (ug/ml) (*E. coli* ATCC 25922)		Toxicity (LC_{50}, µg/mL) (Human Red Blood Cell)
			MHB	CaMHB	
InitScreen-1	WIWWWKKVIEWIRKIRRIIR	9.77	64	> 128	64.4
InitScreen-2	WRIIRRWKKKIKPWIIRIRI	10.23	64	> 128	> 512
InitScreen-3	KRRLRPIIGRVIRKVKFWLR	5.01	16	64	111.84
InitScreen-4	RFARAIYRIIRRLLRRGYCR	6.31	64	128	> 512
InitScreen-5	KKAVFKKLFKGLLKIFAFLG	4.37	> 128	> 128	74.78
InitScreen-6	LFRVWKWIAKIGGKVLKLFL	7.59	32	64	67.42
InitScreen-7	GLWSKIARFAKKLASWLIKS	6.03	> 128	> 128	27.55
InitScreen-8	WKKFWAGLAGHVGKAILSGI	6.92	128	> 128	> 512
InitScreen-9	GWWGKVVKSIIKAVNKGLSK	4.9	64	128	> 512
InitScreen-10	GWLSRFGRLLKRIIRIRPWR	10	32	64	66.43
Pexiganan (Published)		Not Applicable	8	16	> 512
Hydraganan-1 (Published)		Not Applicable	4	8	> 512
Colistin (FDA-Approved)		Not Applicable	4	1	> 512

Supplementary Figures

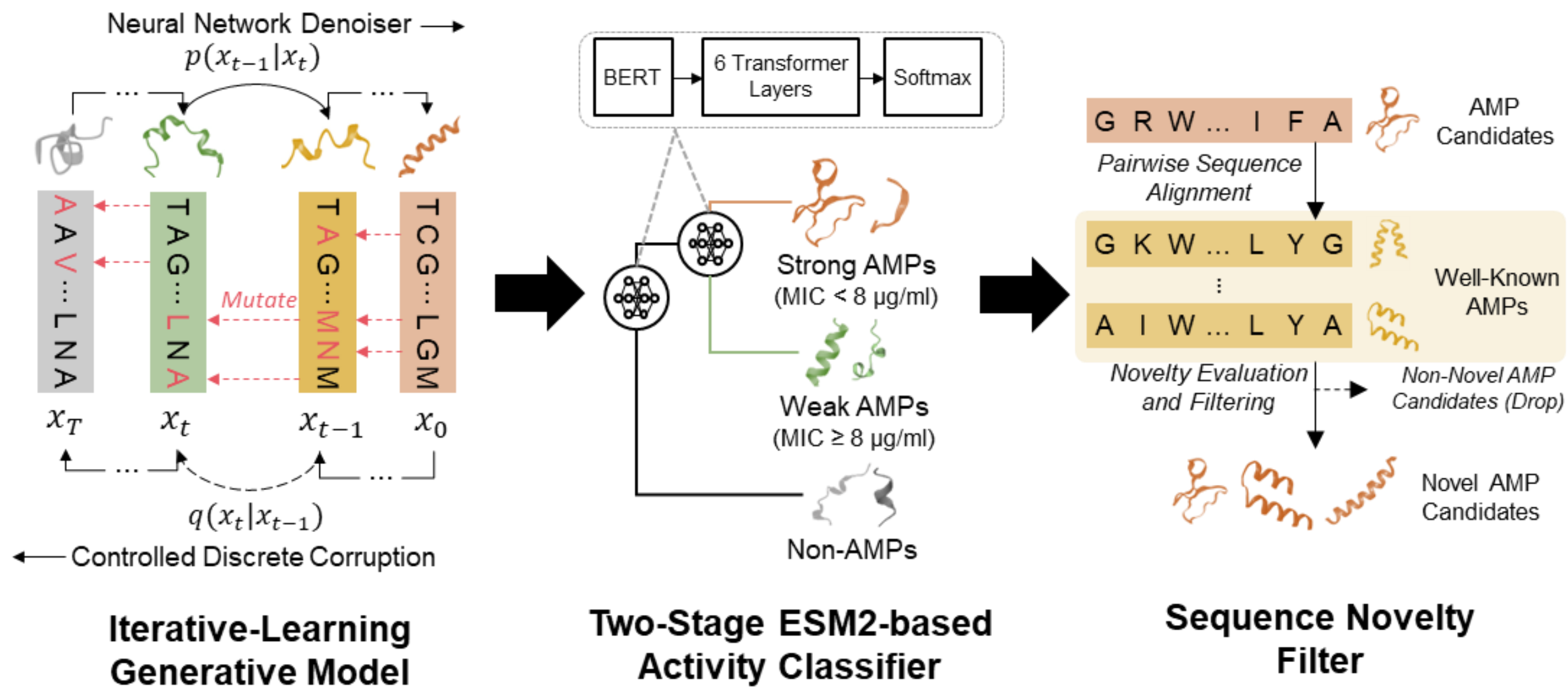

Supplementary Figure 1. The ARCADIAMP platform, consisting of an iterative-learning EvoDiff-D3PM-based generative model, a two-stage ESM2-based activity classifier, and a sequence novelty filter.

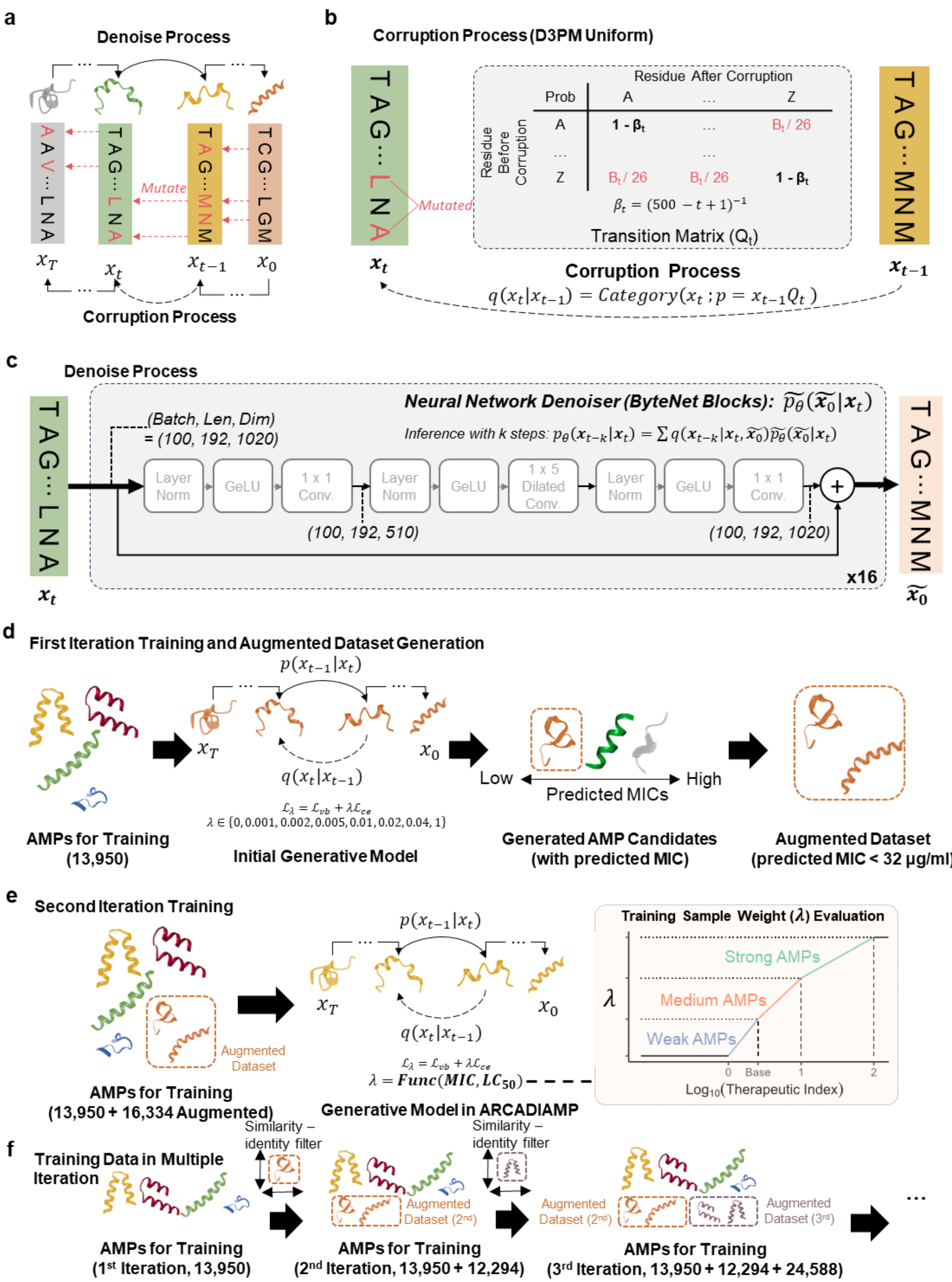

Supplementary Figure 2. Overview of the EvoDiff-D3PM-based generative model. **a.** Schematic of the discrete diffusion model, including the corruption and denoising

processes. **b.** Corruption process with the transition matrix. Noise scheduling is applied such that the corruption rate (β_t) changes across timesteps. **c.** Neural network denoiser used to generate AMP candidates from corrupted sequences. **d.** First iteration of model training and preparation of the augmented dataset. **e.** Second iteration of training using the augmented dataset with sample-specific cross-entropy weights (λ) applied to each sequence. f. Extension of the augmented dataset for additional training iterations.

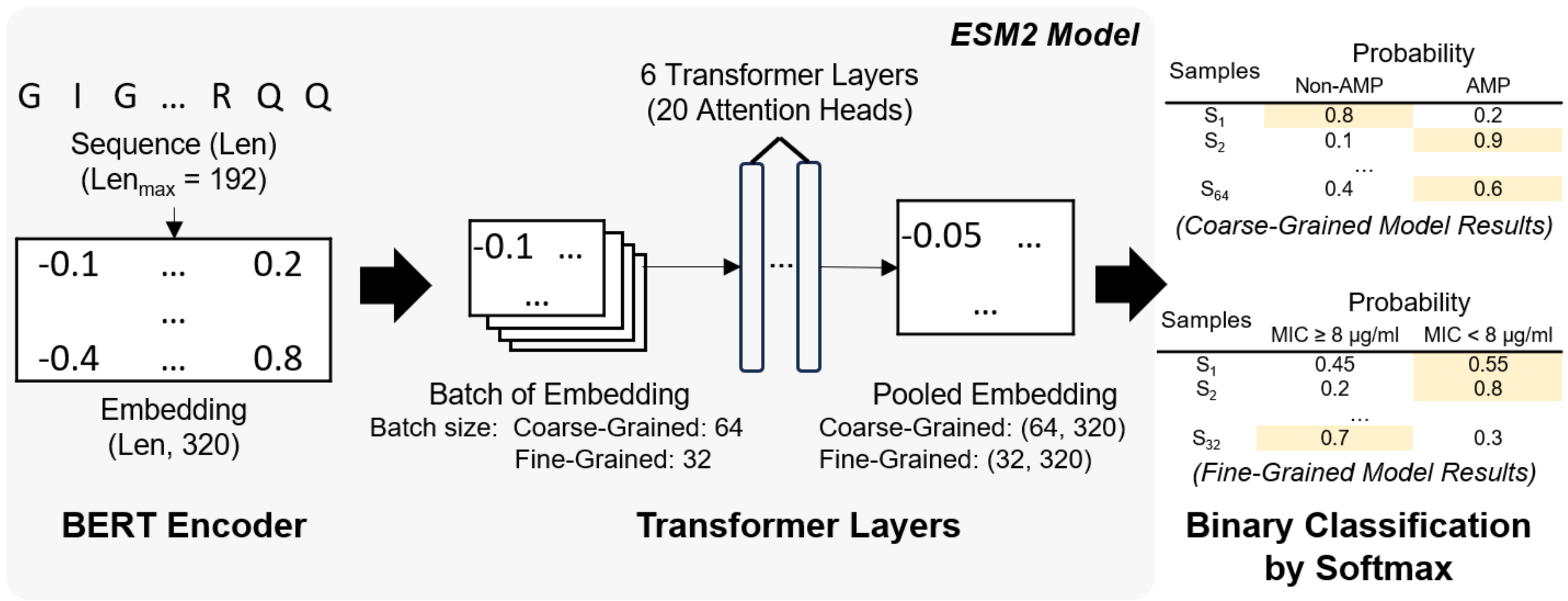

Supplementary Figure 3. Architecture of the ESM2-based antibacterial activity classifier. Both coarse-grained and fine-grained models used the same architecture with different batch sizes. The 8M-parameter version contains six transformer layers with 20 attention heads and processes embeddings of length 320.

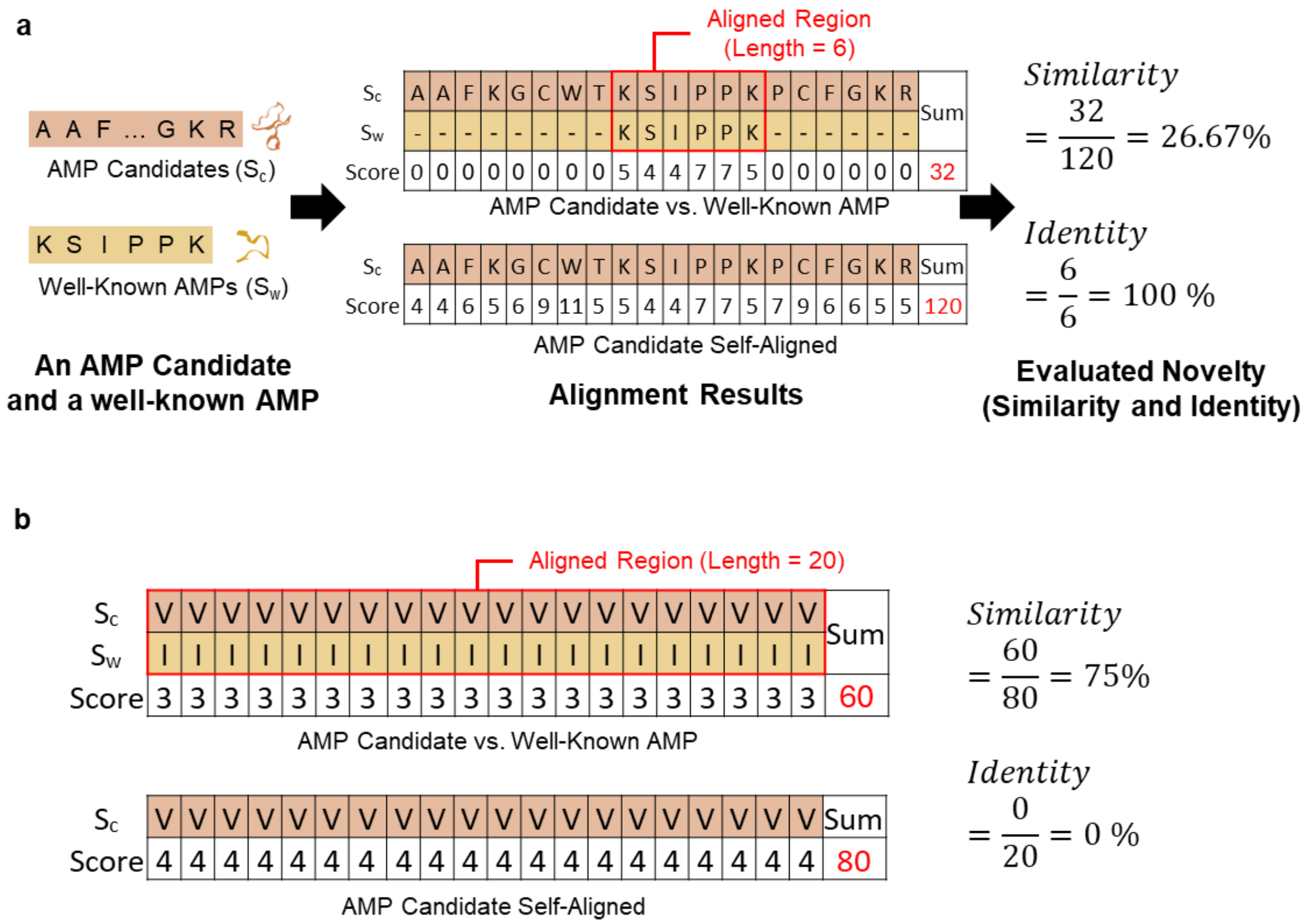

Supplementary Figure 4. Sequence novelty filter. **a.** Evaluation of similarity and identity for an AMP candidate. In this example, the candidate shows high identity to a known AMP when it contains a subsequence similar to that AMP. **b.** Example where the candidate has low identity but high similarity because the candidate and the known AMP share residues with similar properties, though not identical, as determined by the BLOSUM62 substitution matrix.

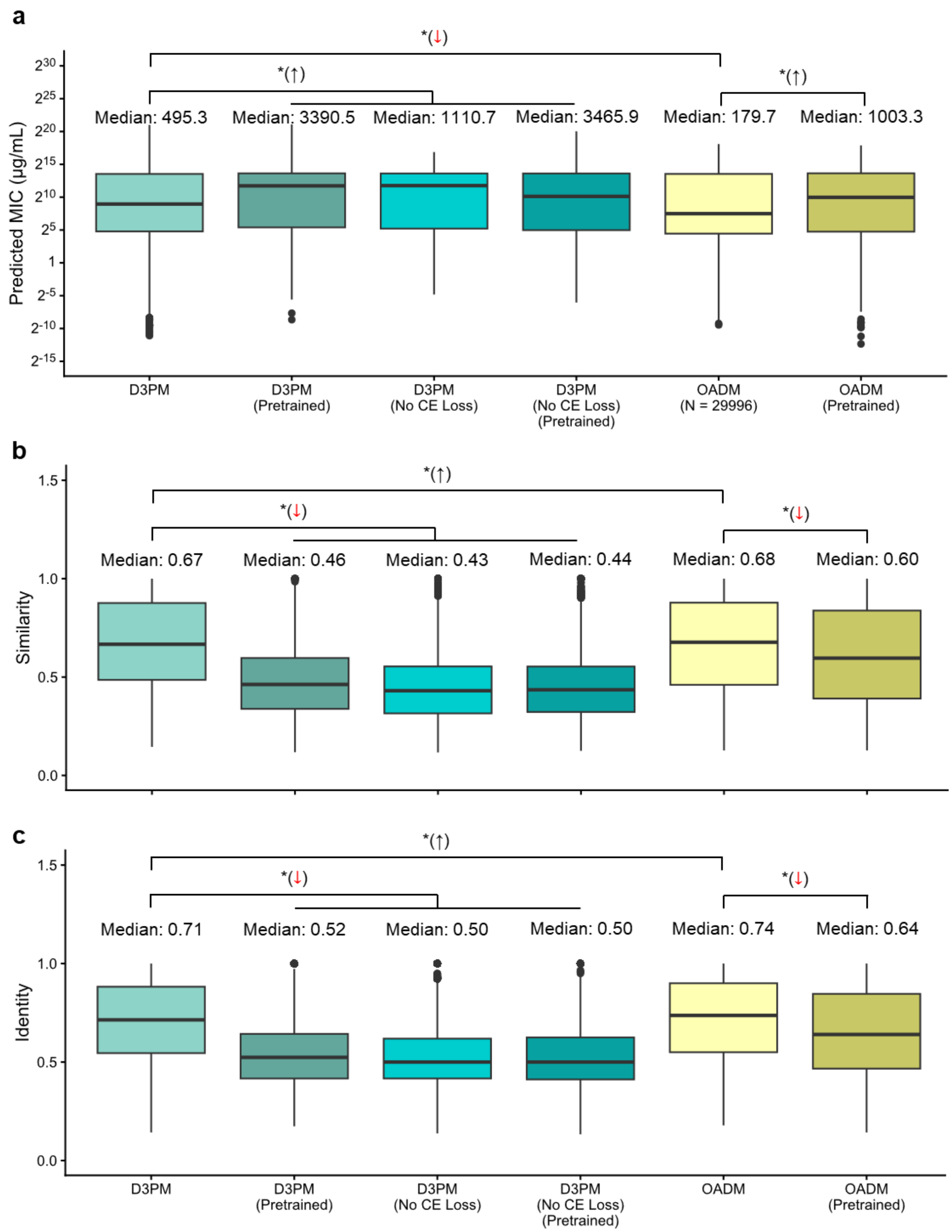

Supplementary Figure 5. Performance comparison of D3PM-based and OADM-based models. a. predicted MICs, **b.** similarity, and **c.** identity of 30,000 generated sequences from D3PM and OADM models were compared. Fine-tuned models based on

pretrained models, as well as the D3PM model trained without cross-entropy loss, were also included. The box boundaries and center correspond to Q1, Q2, and Q3, respectively, where Q1, Q2, and Q3 represent the 25th, 50th, and 75th percentiles. Whiskers extend to 1.5 × (Q3 − Q1) (Log conversion is applied before calculating the length of whiskers if applicable).

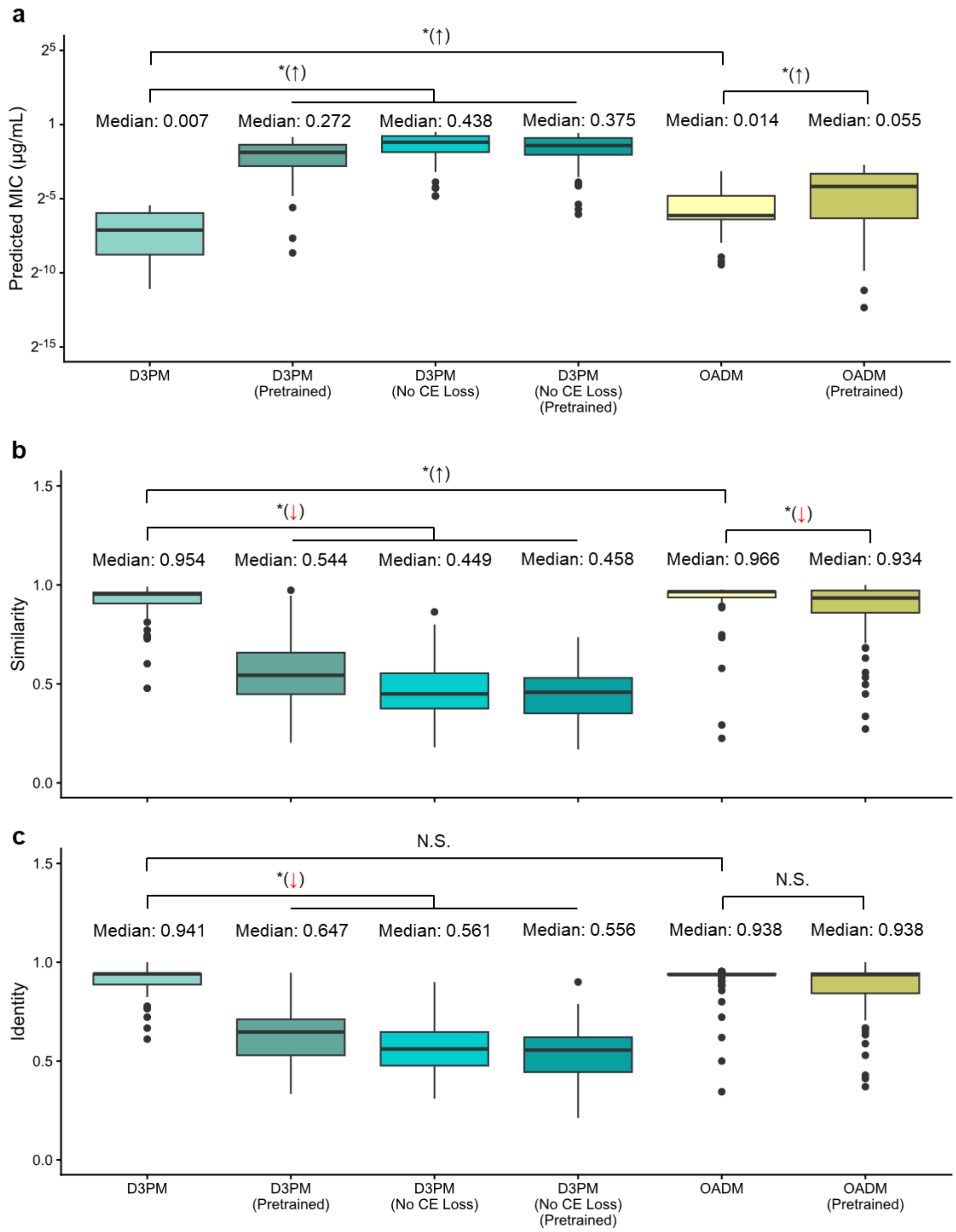

Supplementary Figure 6. Performance comparison of D3PM-based and OADM-based models for the top 100 sequences with the strongest predicted antibacterial activity. a. Predicted MICs, **b.** similarity, and **c.** identity of top 100 sequences, selected from 30,000 generated sequences from D3PM and OADM models, were compared. Fine-

tuned models based on pretrained models, as well as the D3PM model trained without cross-entropy loss, were also included. The box boundaries and center correspond to Q1, Q2, and Q3, respectively, where Q1, Q2, and Q3 represent the 25th, 50th, and 75th percentiles. Whiskers extend to 1.5 × (Q3 − Q1) (Log conversion is applied before calculating the length of whiskers if applicable).

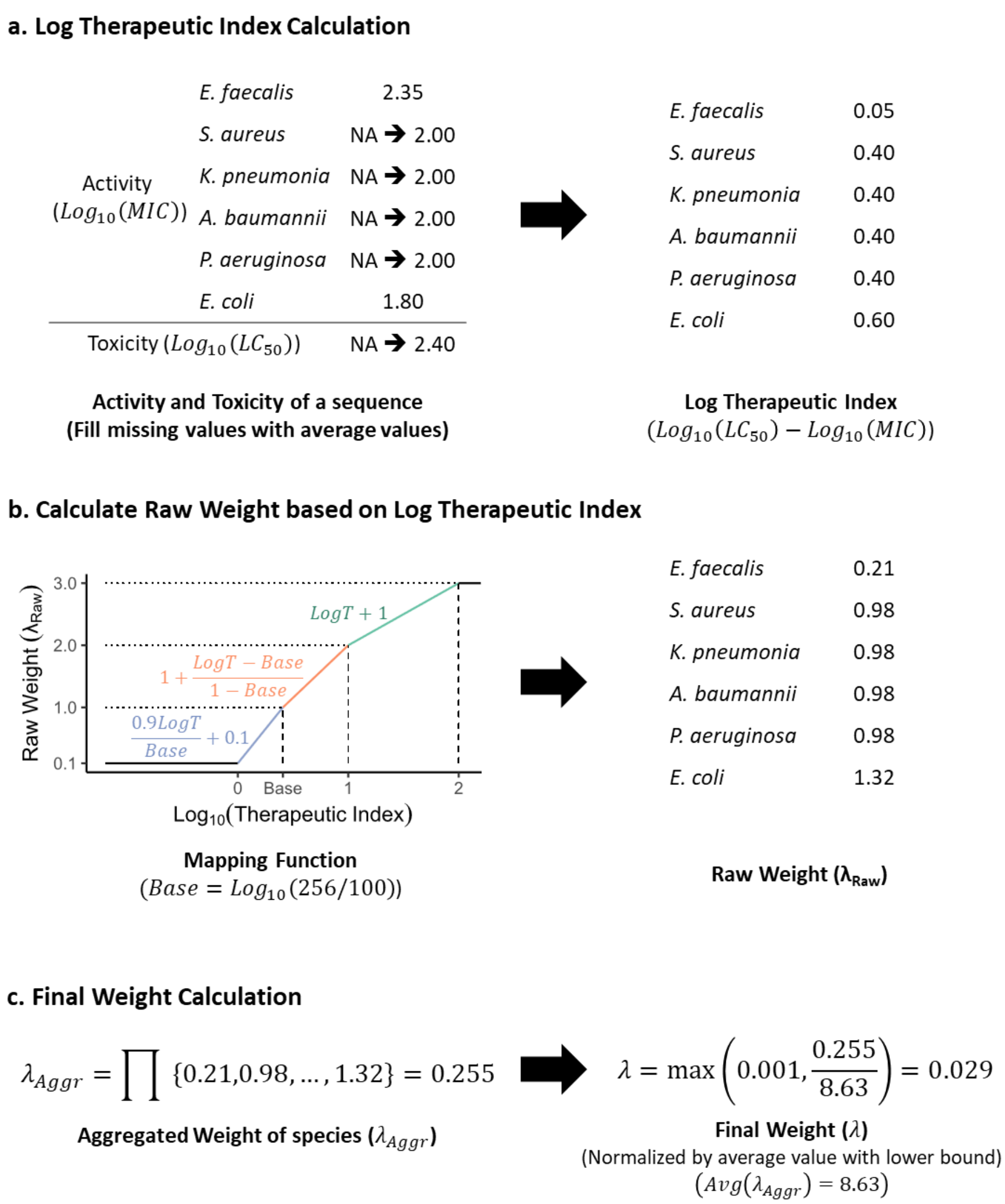

Supplementary Figure 7. Calculation of sample-specific cross-entropy weighting (λ) for generative model training. **a.** The Log therapeutic index of each sequence was calculated based on antibacterial activity (MICs against ESKAPE species) and toxicity (LC_{50} against human red blood cells). Missing values were imputed with average values. **b.** Raw weights were assigned using a predefined mapping function. **c.** Final weights were obtained by taking the product of the raw weights across species, normalizing by the average aggregated weight of all sequences, and applying a lower bound of 0.001.

Peptides were weighted according to therapeutic index [$Log_{10}(LC_{50})$ - Log_{10}(MIC)] via a piecewise mapping function, and the resulting values were normalized across species.

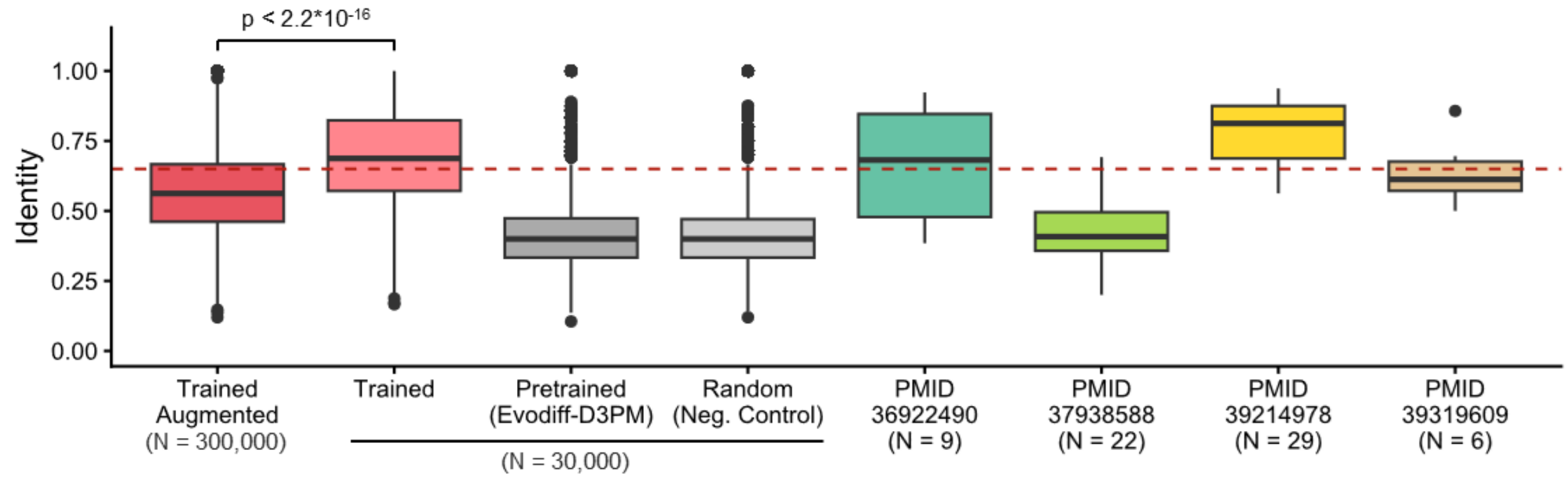

Supplementary Figure 8. Sequence novelty analysis showing the sequence identity of peptides generated by the models relative to published AMPs. Statistical significance was assessed using a two-sided Wilcoxon rank-sum test. The box boundaries and center correspond to Q1, Q2, and Q3, respectively, where Q1, Q2, and Q3 represent the 25th, 50th, and 75th percentiles. Whiskers extend to 1.5 × (Q3 − Q1).

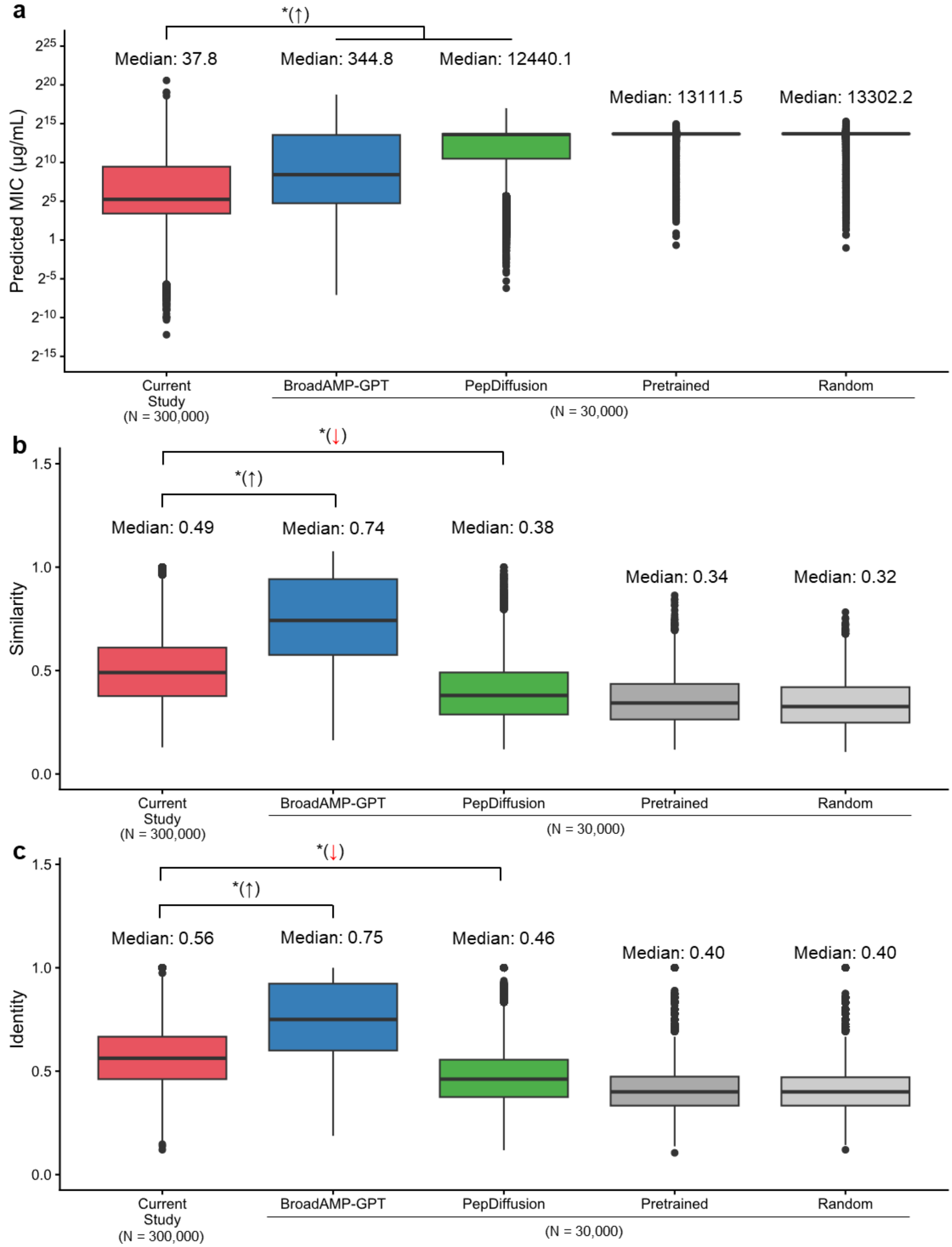
a
*(↑)
Median: 37.8
Median: 344.8
Median: 12440.1
Median: 13111.5
Median: 13302.2
Predicted MIC (μg/mL)
Current Study (N = 300,000)
BroadAMP-GPT
PepDiffusion
Pretrained
Random
(N = 30,000)
b
*(↓)
*(↑)
Median: 0.49
Median: 0.74
Median: 0.38
Median: 0.34
Median: 0.32
Similarity
c
*(↓)
*(↑)
Median: 0.56
Median: 0.75
Median: 0.46
Median: 0.40
Median: 0.40
Identity

Supplementary Figure 9. Performance comparison of different published models. **a.** Predicted MICs, **b.** similarity, and **c.** identity of generated sequences from the model in the current study (trained with augmented training sequences) and two other published models were compared. Two negative-control models - the pretrained D3PM model (without AMP training) and a random sequence generator - were also included. The box boundaries and center correspond to Q1, Q2, and Q3, respectively, where Q1, Q2, and Q3 represent the 25th, 50th, and 75th percentiles. Whiskers extend to 1.5 × (Q3 − Q1) (Log conversion is applied before calculating the length of whiskers if applicable).

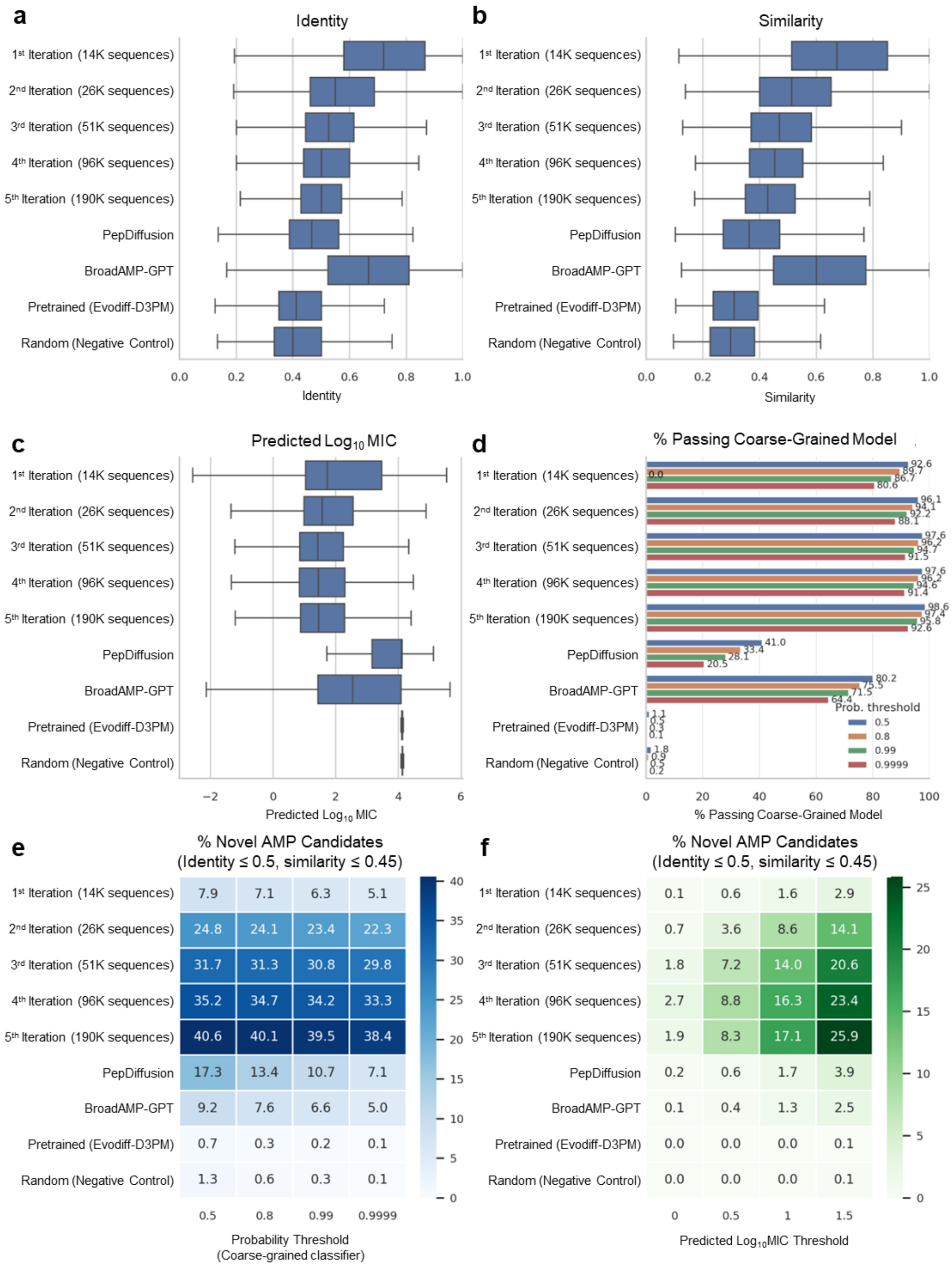

Supplementary Figure 10. Generative model performance across successive augmented training iterations. a. Sequence identity and **b.** sequence similarity of generated sequences relative to the closest known AMP, and **c.** predicted MIC values

(Log_{10} scale) estimated by the Huang et al. regression model, for models trained across one to five augmented training iterations. Models trained without sample-wise weighting, a pretrained D3PM baseline, a random-sequence generator, and two published generative models (BroadAMP-GPT and Pep-Diffusion) are included for comparison. **d.** Percentage of generated sequences exceeding the AMP activity probability threshold of the coarse-grained classifier. **e.** Percentage of novel generated sequences — defined as those with identity ≤ 0.50 and similarity ≤ 0.45 relative to known AMPs — exceeding the same AMP activity probability threshold. **f.** Percentage of novel generated sequences with predicted MICs (Log_{10} scale) below specified activity thresholds, as estimated by the Huang et al. regression model. The box boundaries and center correspond to Q1, Q2, and Q3, respectively, where Q1, Q2, and Q3 represent the 25th, 50th, and 75th percentiles. Whiskers extend to 1.5 × (Q3 − Q1) (Log conversion is applied before calculating the length of whiskers if applicable).

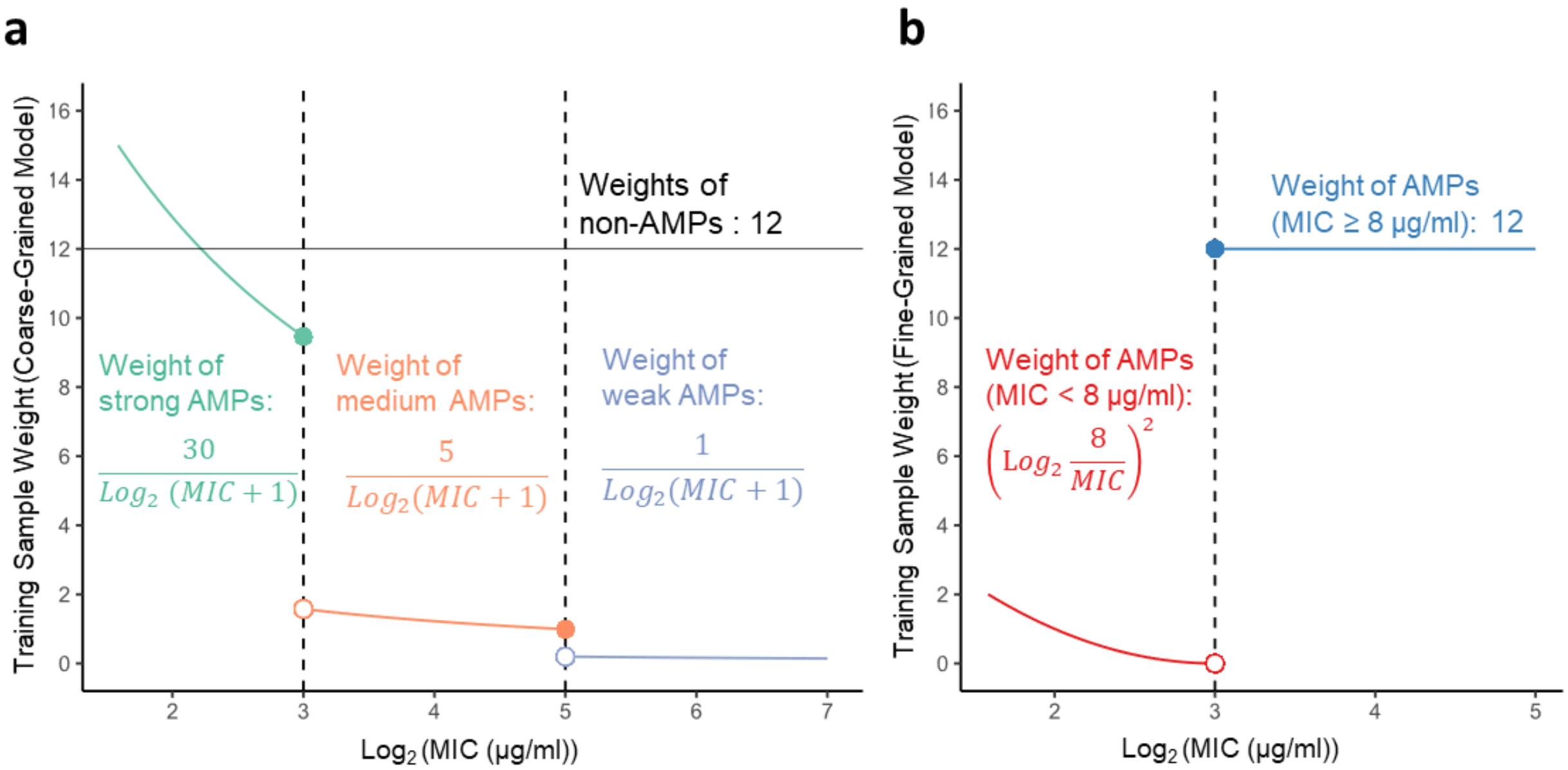

Supplementary Figure 11. Sample weight mapping functions for training the activity classifiers. **a.** Coarse-grained classifier (AMP vs non-AMP). **b.** Fine-grained classifier (strong AMP vs weak AMP).

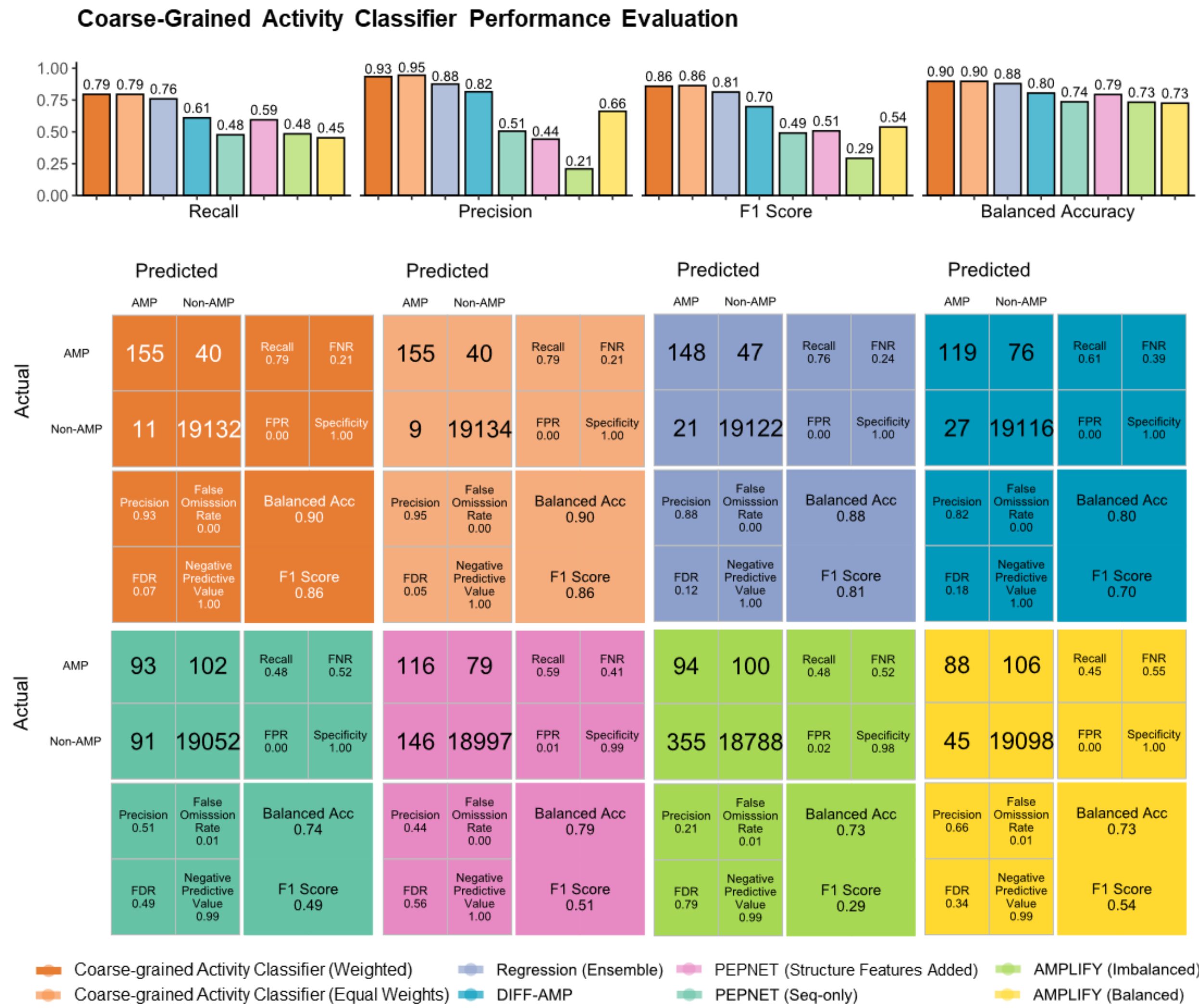

Supplementary Figure 12. Binary classification benchmarks (recall, precision, F1, and balanced accuracy, using the threshold yielding the highest F1-score) of the coarse-grained activity classifier and other published classifiers for classifying AMPs. Corresponding confusion matrices are shown.

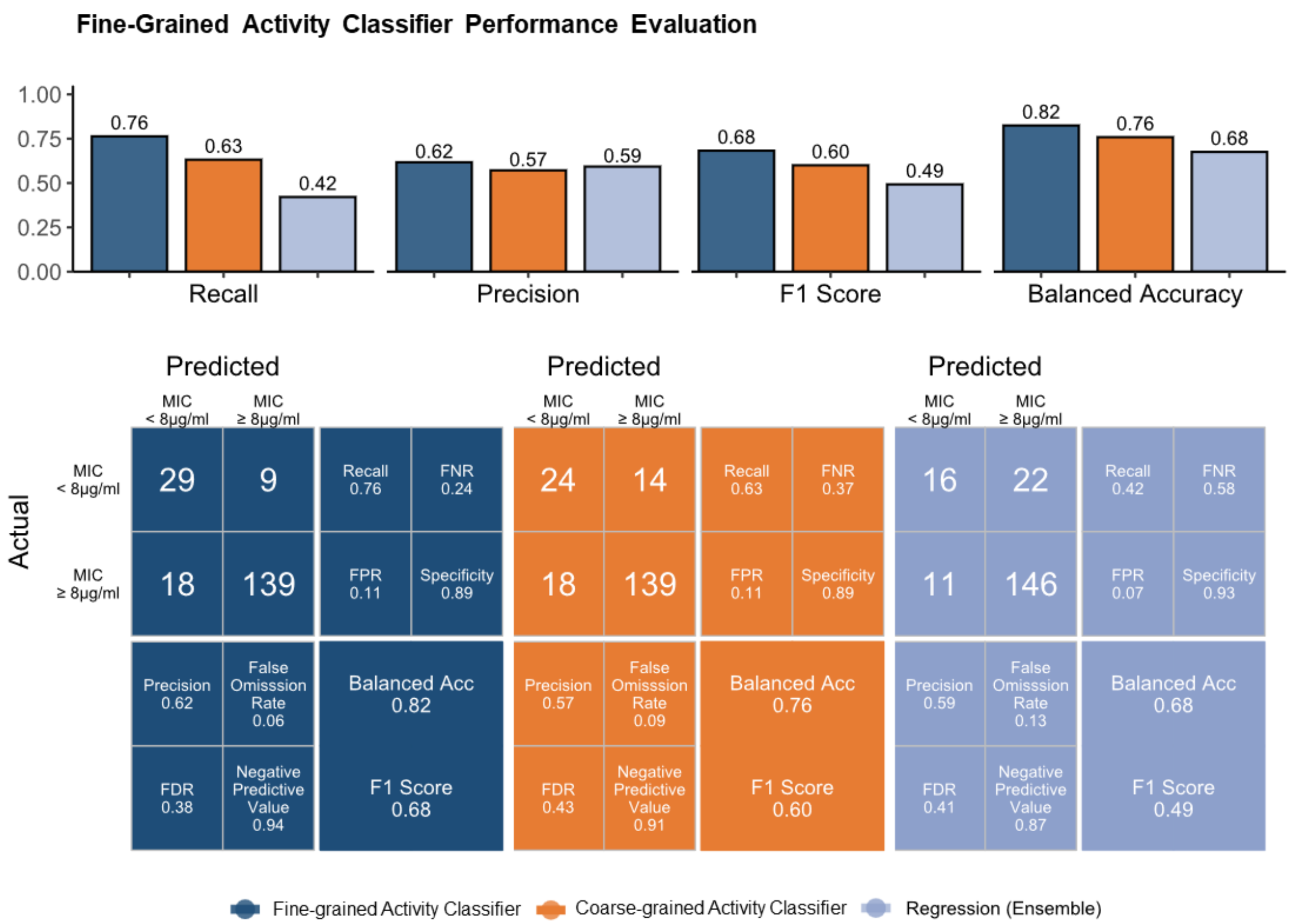

Supplementary Figure 13. Binary classification benchmarks (recall, precision, F1, and balanced accuracy, using the threshold yielding the highest F1-score) and the corresponding confusion matrix of the fine-grained activity classifier for classifying strong AMPs (MIC < 8 µg/mL). The coarse-grained activity classifier and ensemble MIC regression models were also applied for the performance comparison.

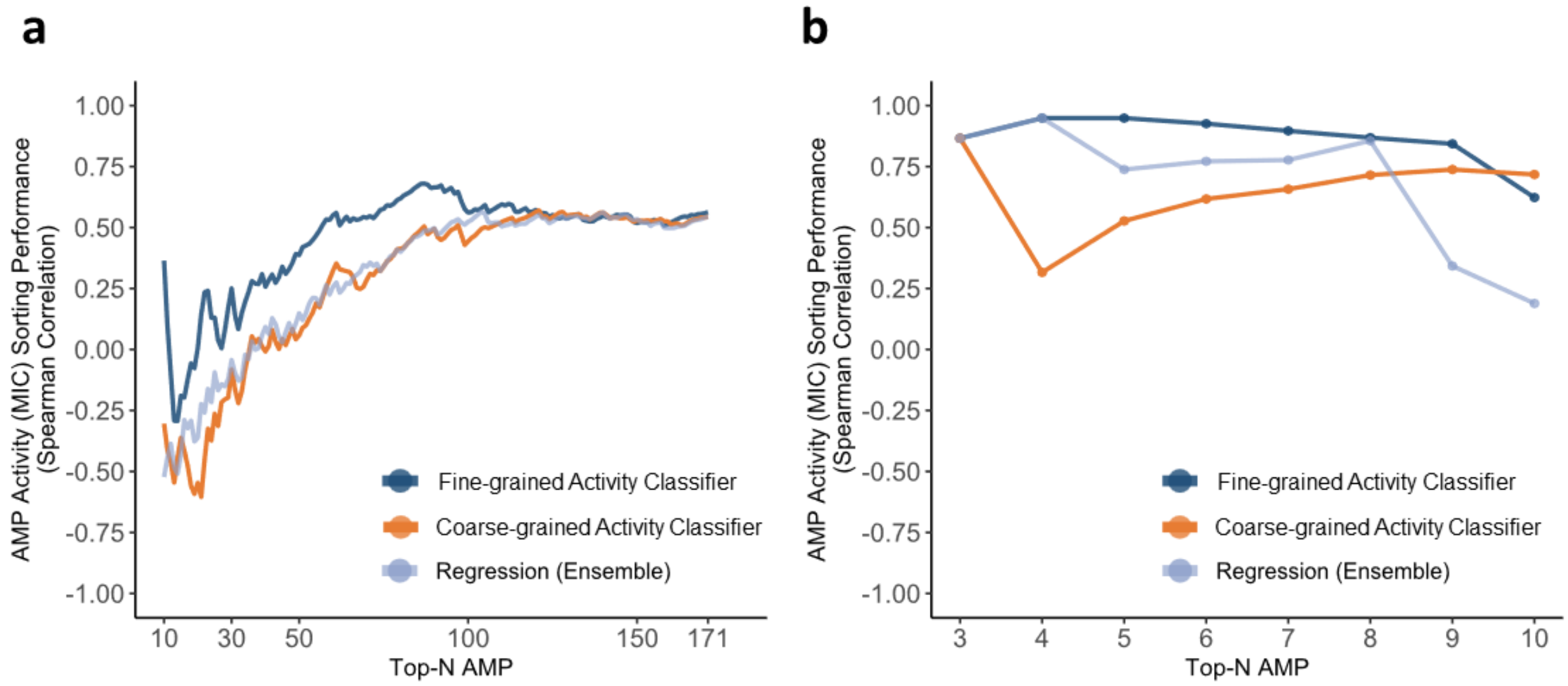

Supplementary Figure 14. Spearman correlation between predicted rank (based on probability or MIC) and actual MIC. **a.** Test set comparison among fine-grained, coarse-grained, and ensemble models. **b.** Ten candidates (by initial screening) ranked by the fine-grained model compared with coarse-grained and ensemble models.

Peptide	*E. faecalis* ATCC 29212			*S. aureus* ATCC 25923			*K. pneumonia* ATCC 700603			*A. baumannii* ATCC 19606			*P. aeruginosa* ATCC 27853			*E. coli* ATCC 25922			HEK293	Human red blood cell
	MH	CaMH	Serum	MH	CaMH	Serum	MH	CaMH	Serum	MH	CaMH	Serum	MH	CaMH	Serum	MH	CaMH	Serum		
Peptide 1	128	>128	>128	32	>128	>128	32	>128	>128	32	>128	>128	16	>128	>128	64	>128	>128	160.79	>512
Peptide 2	>128	>128	>128	16	64	64	8	8	32	8	8	64	8	32	>128	8	8	128	114.66	>512
Peptide 3	64	>128	>128	32	128	64	32	128	128	32	128	32	32	128	>128	32	64	128	45.94	>512
Peptide 4	64	128	>128	32	128	128	32	128	>128	32	64	64	32	128	>128	32	32	128	70.05	>512
Peptide 5	128	>128	>128	32	128	32	32	128	>128	32	128	>128	16	>128	>128	32	64	>128	81.64	>512
Peptide 6	64	>128	>128	16	128	32	32	128	>128	16	32	32	16	>128	>128	16	32	128	60	>512
Peptide 7	64	>128	>128	16	32	32	8	16	32	8	16	32	8	32	>128	8	16	32	50.84	>512
Peptide 8	64	>128	>128	32	128	128	32	128	>128	32	>128	>128	32	>128	>128	32	64	>128	113.1	>512
Peptide 9	>128	>128	>128	64	>128	>128	>128	>128	>128	>128	>128	>128	>128	>128	>128	64	>128	>128	81.21	>512
Peptide 10	>128	>128	>128	32	128	>128	128	>128	>128	16	64	>128	8	128	>128	32	64	>128	79.62	>512
Pexiganan	32	128	64	16	32	8	8	16	4	8	8	8	8	16	64	8	16	64	37.75	>512
Hydraganan-1	32	>128	128	16	16	32	16	>128	>128	4	8	>128	16	>128	>128	4	8	>128	40.53	>512
Colistin	>128	>128	>128	>128	>128	>128	2	1	1	1	0.5	0.5	2	1	1	1	2	2	5.32	NA
Daptomycin	16	2	2	32	2	0.5	>128	>128	>128	>128	>128	>128	>128	>128	>128	>128	>128	>128	NA	NA
Melittin	8	4	32	8	4	32	32	128	>128	8	4	64	32	128	>128	8	16	>128	NA	NA

Peptide	*S. aureus* ATCC 25923			*E. coli* ATCC 25922			HEK293	Human red blood cell
	MH	CaMH	Serum	MH	CaMH	Serum		
Peptide 1	32	>128	>128	64	>128	>128	160.79	>512
Peptide 2	16	64	64	8	8	128	114.66	>512
Peptide 3	32	128	64	32	64	128	45.94	>512
Peptide 4	32	128	128	32	32	128	70.05	>512
Peptide 5	32	128	32	32	64	>128	81.64	>512
Peptide 6	16	128	32	16	32	128	60	>512
Peptide 7	16	32	32	8	16	32	50.84	>512
Peptide 8	32	128	128	32	64	>128	113.1	>512
Peptide 9	64	>128	>128	64	>128	>128	81.21	>512
Peptide 10	32	128	>128	32	64	>128	79.62	>512
Pexiganan	16	32	8	8	16	64	37.75	>512
Hydraganan-1	16	16	32	4	8	>128	40.53	>512
Melittin	8	4	32	8	16	>128	5.32	NA
Colistin	>128	>128	>128	1	2	2	NA	NA
Daptomycin	32	2	0.5	>128	>128	>128	NA	NA

Comprehensive Safety Score*	
S. aureus	*E. coli*
NA	NA
1.79	0.9
0.72	0.36
0.55	0.55
2.55	NA
1.88	0.47
1.59	1.59
0.88	NA
NA	NA
NA	NA

Comprehensive Safety Score*
: A modified Therapeutic Index defined as $LC50_{HEK293}$ / MIC_{Serum}. This index was designed to prioritize candidates that maintain a high safety margin while retaining potent antimicrobial activity in physiologically relevant environments, and superior stability and potential for in vivo efficacy.

Supplementary Figure 15. This visualized table shows that Peptide-7 (Arcinin) retains potent antimicrobial activity (32 μg/mL) in serum against the primary in vivo targets, *S. aureus* and *E. coli*, while exhibiting a favorable safety profile with low HEK293 toxicity (50.84 μg/mL). Consequently, Arcinin achieves the highest Comprehensive Safety Score (1.59) - a modified therapeutic index defined as the ratio of HEK293 LC_{50} to serum MIC and designed to prioritize candidates with high physiological stability - confirming it as the optimal lead candidate for balancing safety and efficacy in complex *in vivo* environments.

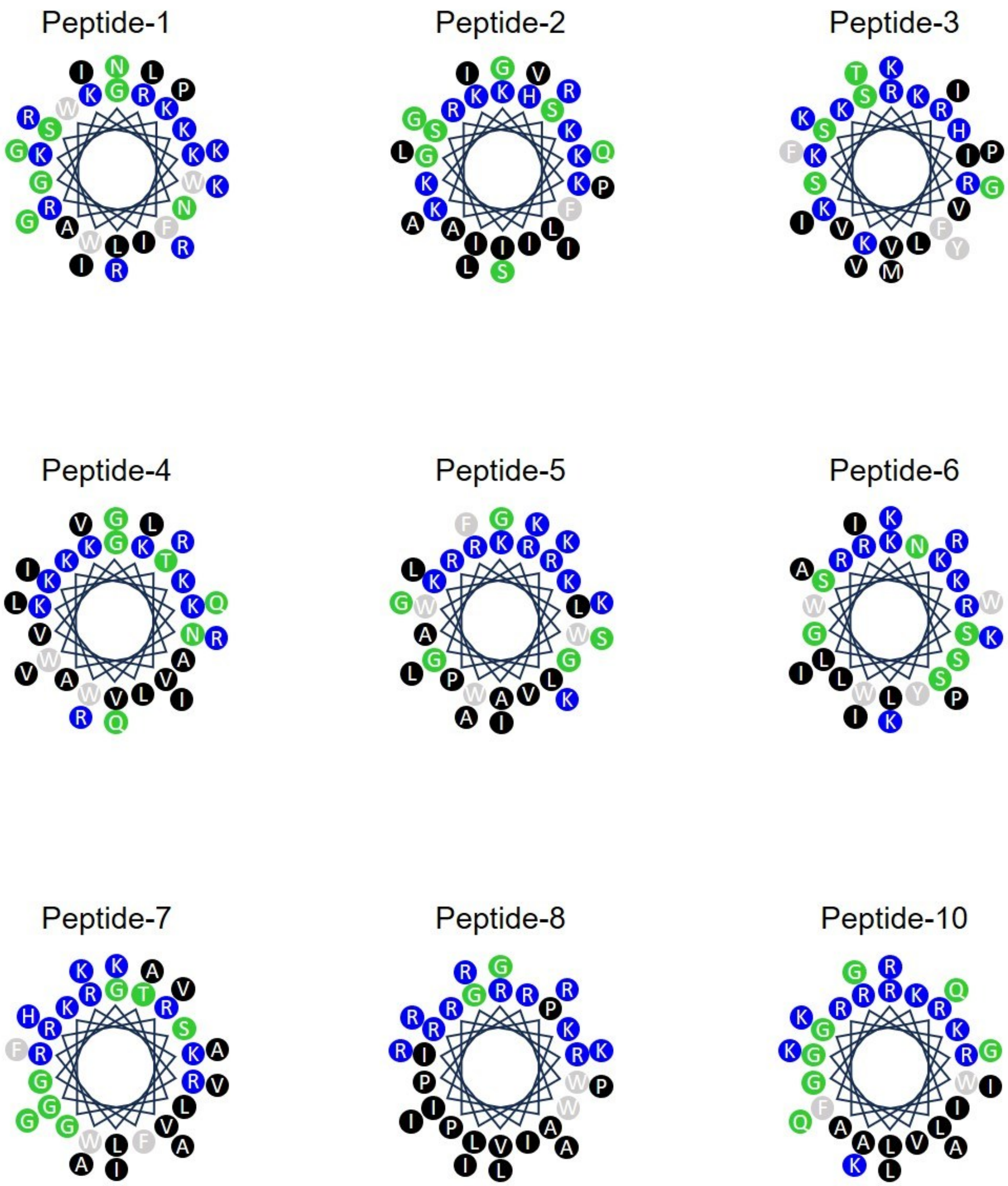

Supplementary Figure 16. Helical-wheel diagrams of active peptide candidates. Of the 10 generated peptides, 9 candidates with favorable antimicrobial activity are shown. In the diagrams, amino-acid residues are color-coded according to their physicochemical properties to visualize facial segregation: blue indicates cationic residues, green indicates neutral hydrophilic residues, black indicates hydrophobic residues, and grey indicates

interfacial residues. These diagrams show that our candidates possess robust amphipathic α-helical structures.

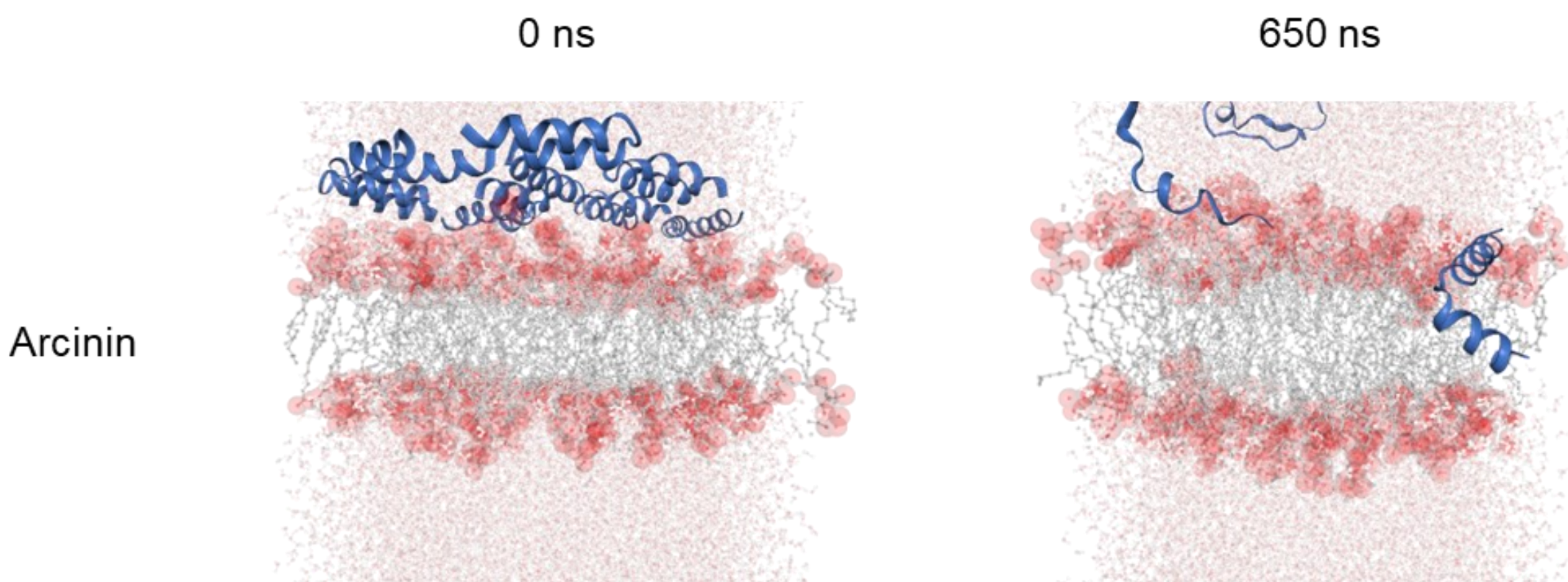

Supplementary Figure 17. Molecular dynamics simulations of a system in which Arcinin did not initially attach to the membrane. Snapshots at 0 ns and 650 ns are shown.

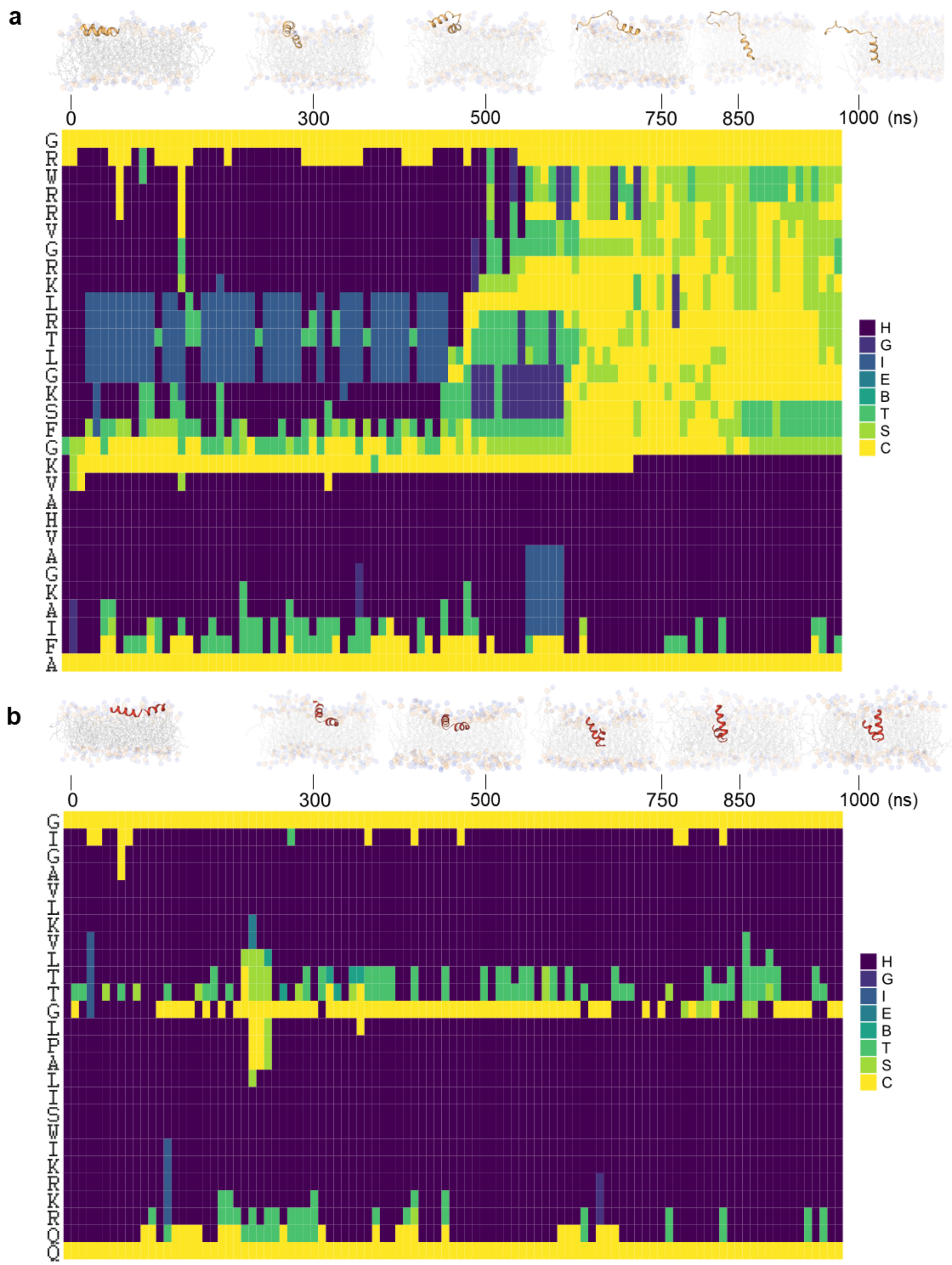

Supplementary Figure 18. Predicted secondary structures of peptides that penetrate the membrane during molecular dynamics simulation. One membrane-penetrating peptide was selected for analysis in each case: **a.** Arcinin, and **b.** Melittin.

The peptide structure was extracted, and its secondary structure was evaluated every 10 ns using the DSSP tool. The outcomes were grouped as helix (H: α-helix, G: 3_{10}-helix, and I: π-helix), strand (B: β-bridge and E: extended strand), or irregular structure (T: hydrogen-bonded turn, S: bend, and C: irregular coil).

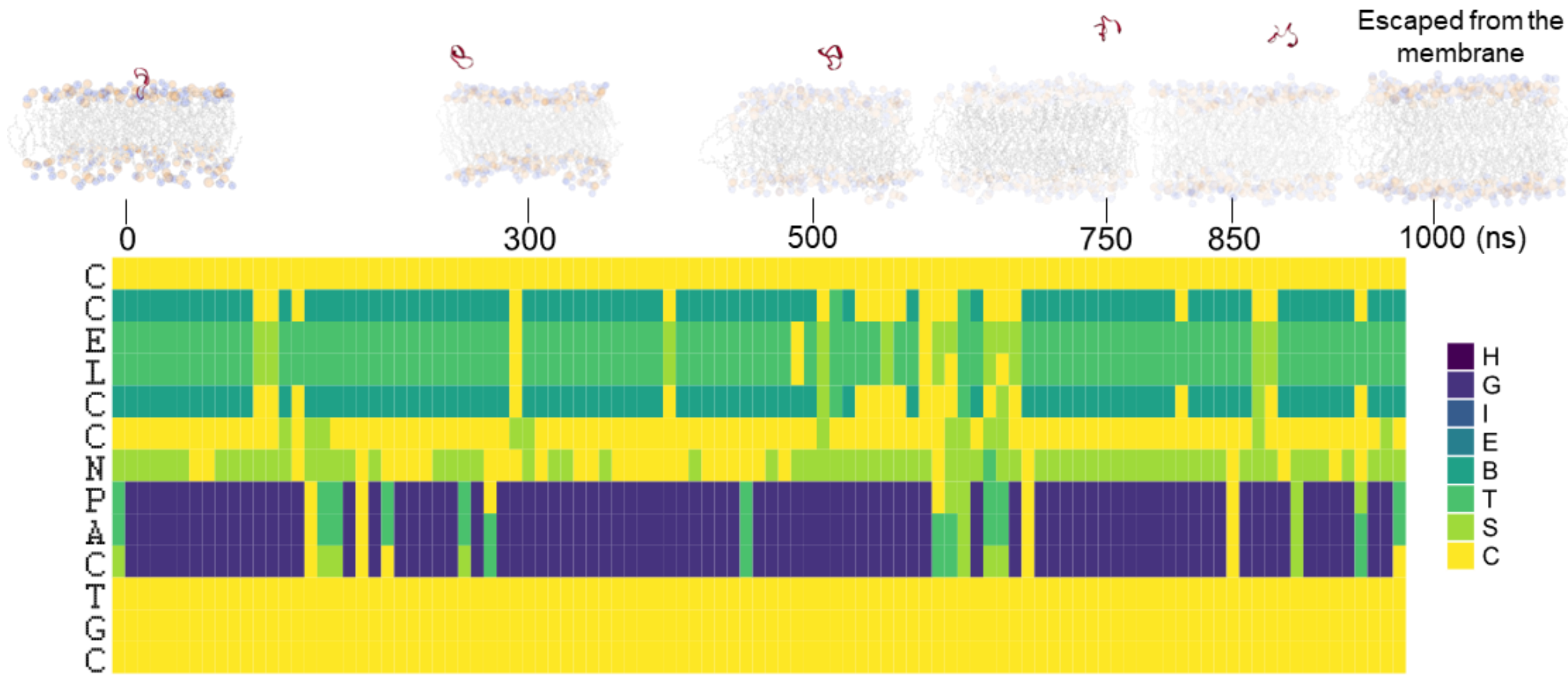

Supplementary Figure 19. Predicted secondary structure of a non-AMP peptide (enterotoxin) during molecular dynamics simulation. One peptide was selected for analysis. The peptide structure was extracted, and its secondary structure was evaluated every 10 ns using the DSSP tool. The outcomes were grouped as helix (H: α-helix, G: 3_{10}-helix, and I: π-helix), strand (B: β-bridge and E: extended strand), or irregular structure (T: hydrogen-bonded turn, S: bend, and C: irregular coil).

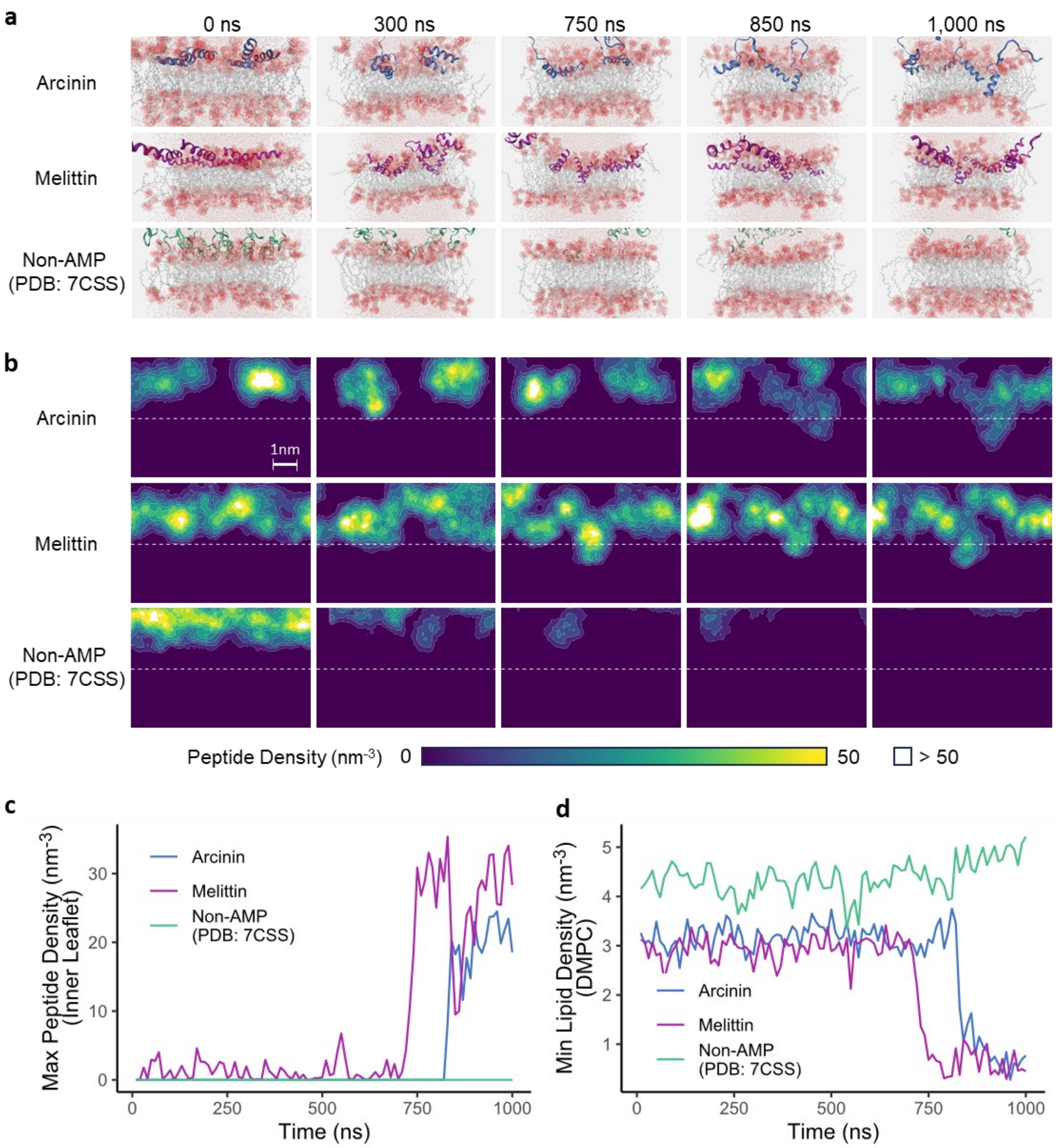

Supplementary Figure 20. Molecular dynamics simulations of peptide-membrane interactions. **a.** Snapshots of peptide-membrane systems (Arcinin, Melittin, non-AMP, PDB ID: 7CSS) at 0, 300, 750, 850, and 1,000 ns. **b.** The density of the peptide inserted in the lipid bilayer across five time points. The white dashed line indicates the bilayer midplane. **c.** Maximum peptide density in the inner leaflet over time. **d.** Minimum lipid density across the membrane over time.

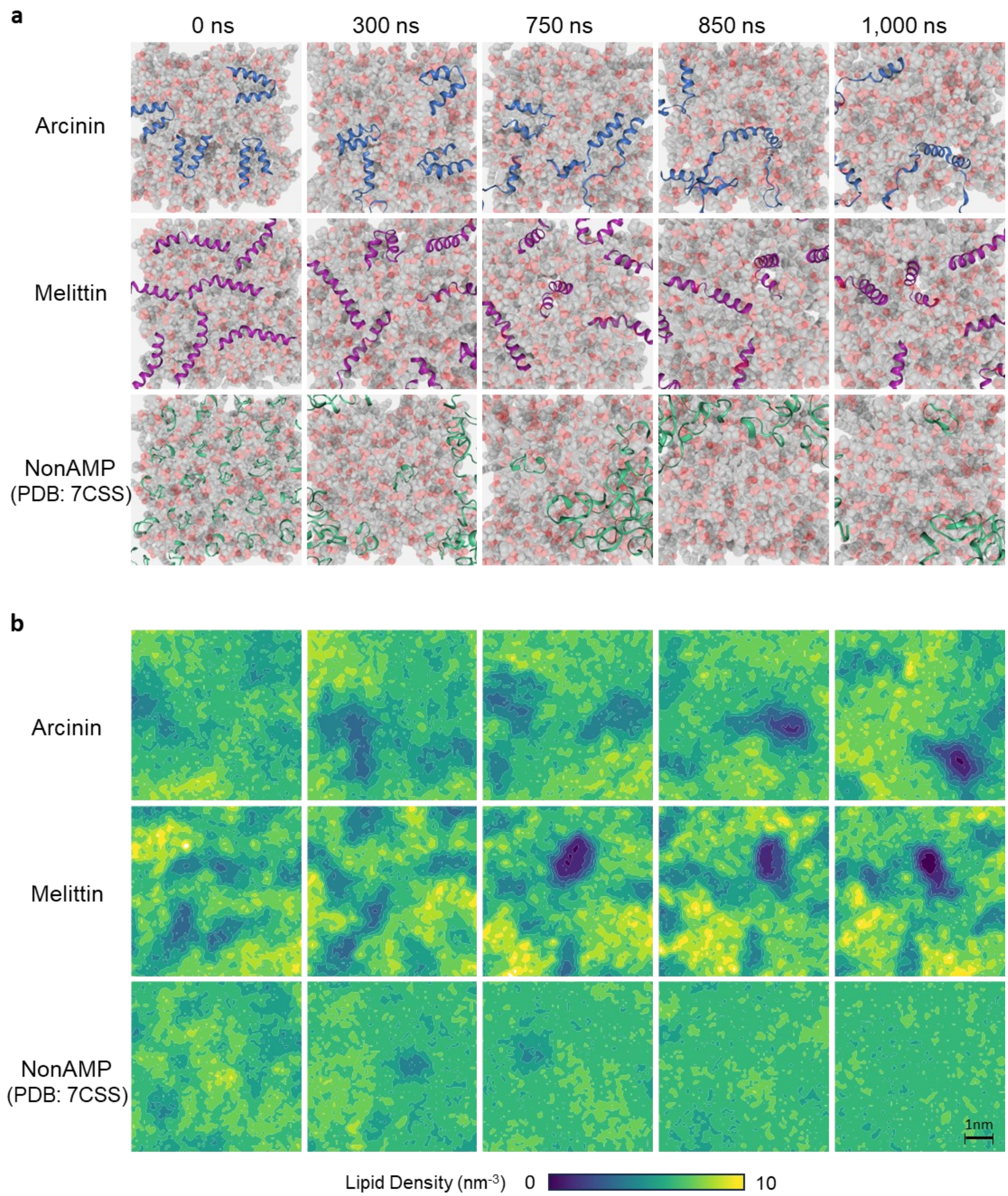

Supplementary Figure 21. a. Top-to-bottom views of the peptide-membrane system at 0 ns, 300 ns, 750 ns, 850 ns, and 1,000 ns. **b.** Corresponding lipid density plots at the same time points.

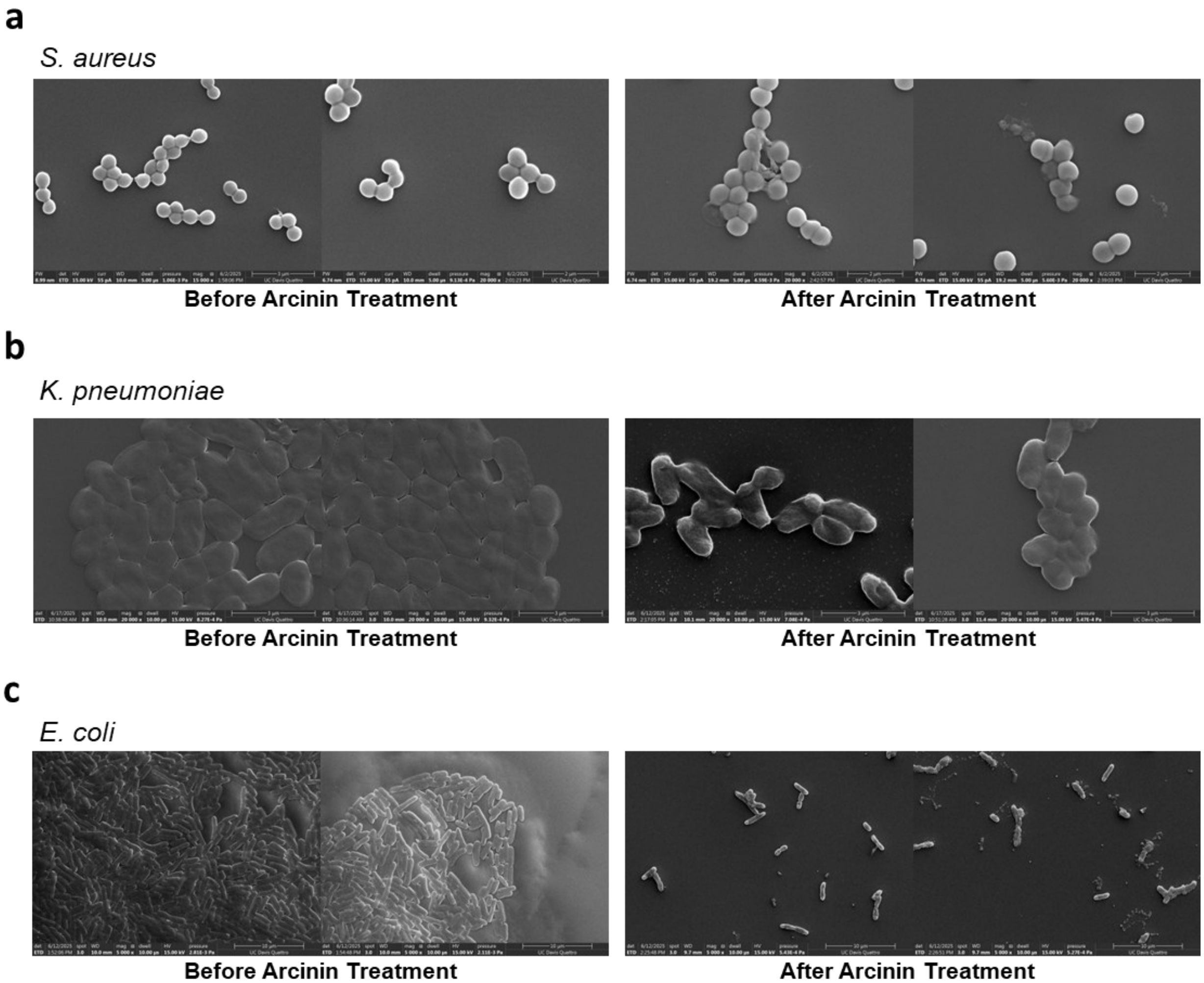

Supplementary Figure 22. Scanning electron microscopy (SEM) images of bacterial populations before (intact) and after treatment with Arcinin. **a.** *S. aureus*: intact cells show characteristic grape-like clusters with smooth surfaces, while treated cells exhibit lysis, debris, and disruption of cluster formations. **b.** *K. pneumoniae*: intact cells form a dense, confluent layer of aggregates, while treated cells display severe surface wrinkling and dispersal from the dense community. **c.** *E. coli*: intact cells form a tightly packed layer, while treated cells show widespread lysis and scattering of individual cells.

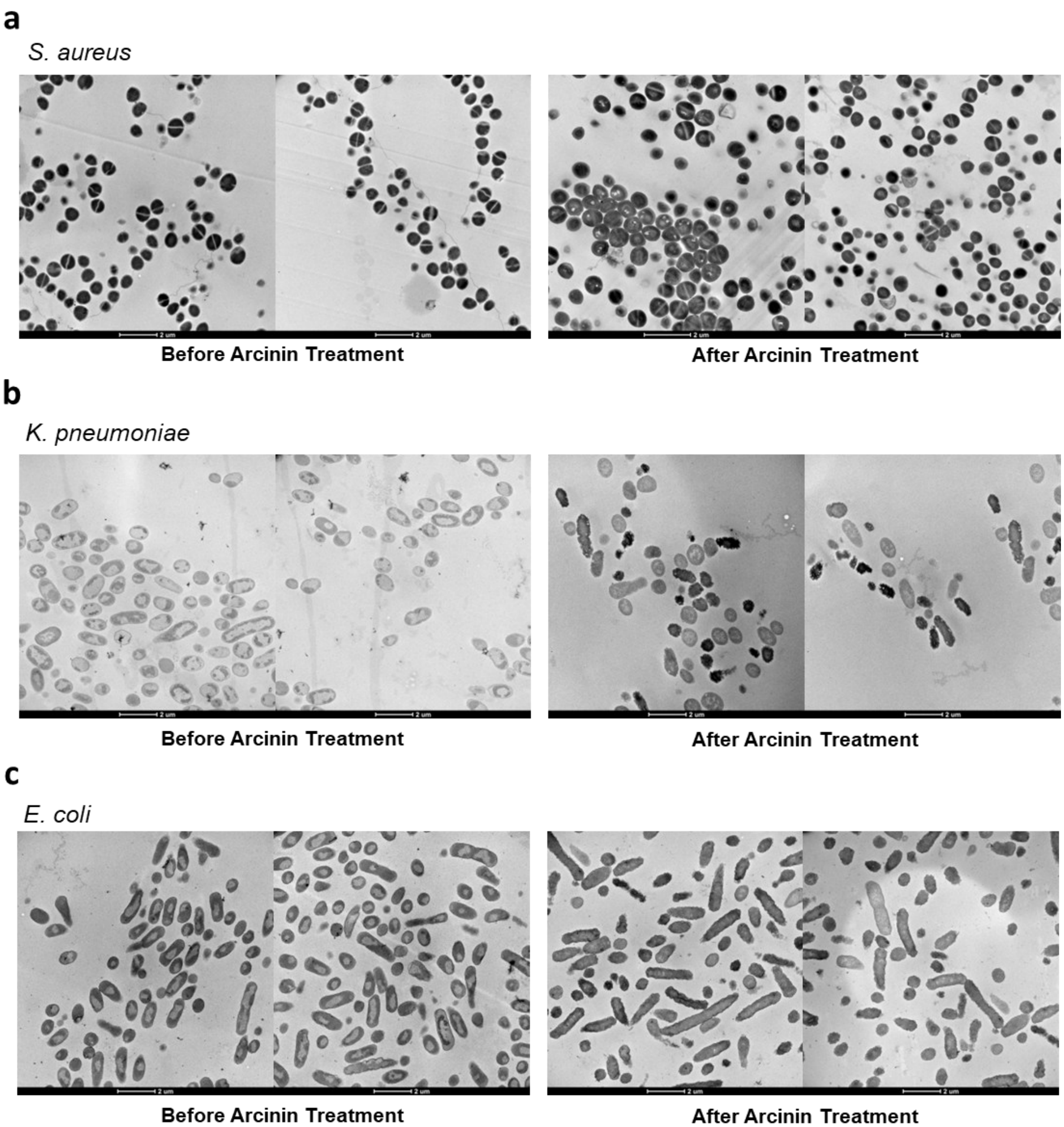

Supplementary Figure 23. Transmission electron microscopy (TEM) images of bacterial populations before (intact) and after treatment with Arcinin. **a.** *S. aureus*: Intact *S. aureus* shows a healthy population with characteristically electron-dense cytoplasm in centrally-sectioned cells, whereas treated cells exhibit widespread cytoplasmic loss and appear electron-lucent, consistent with cell lysis. **b.** *K. pneumoniae*: intact cells have well-

organized, heterogeneous cytoplasm, while treated cells show homogenized internal contents and wrinkled cell surfaces. **c.** *E. coli*: intact cells display a well-structured cytoplasm, while treated cells exhibit homogenized internal contents and a filamentous phenotype with elongated cells due to division inhibition.

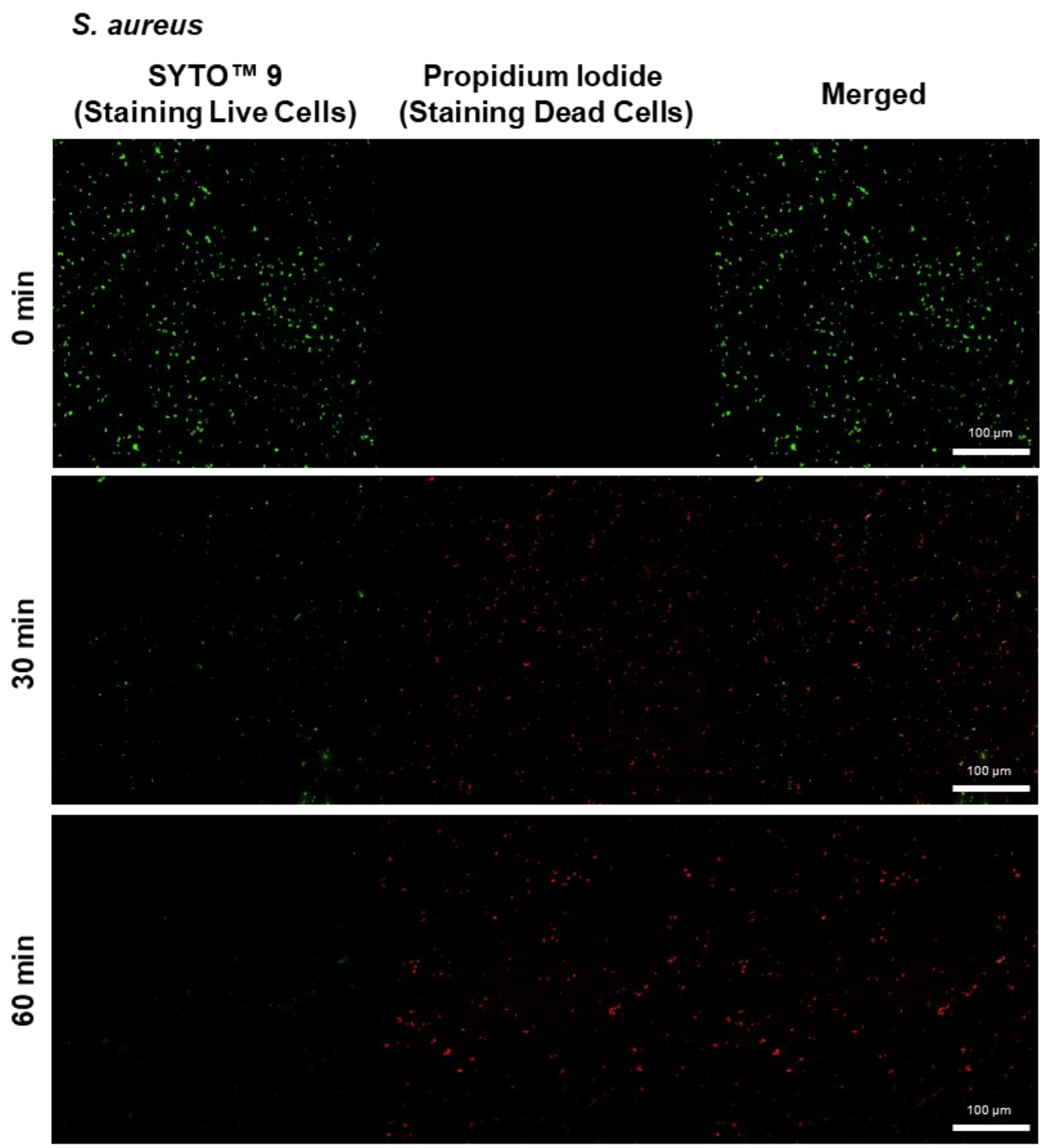

Supplementary Figure 24. Confocal fluorescence microscopy of *S. aureus* stained with a LIVE/DEAD viability dye. Images were captured at 0, 30, and 60 minutes after Arcinin treatment. Scale bars = 100 µm.

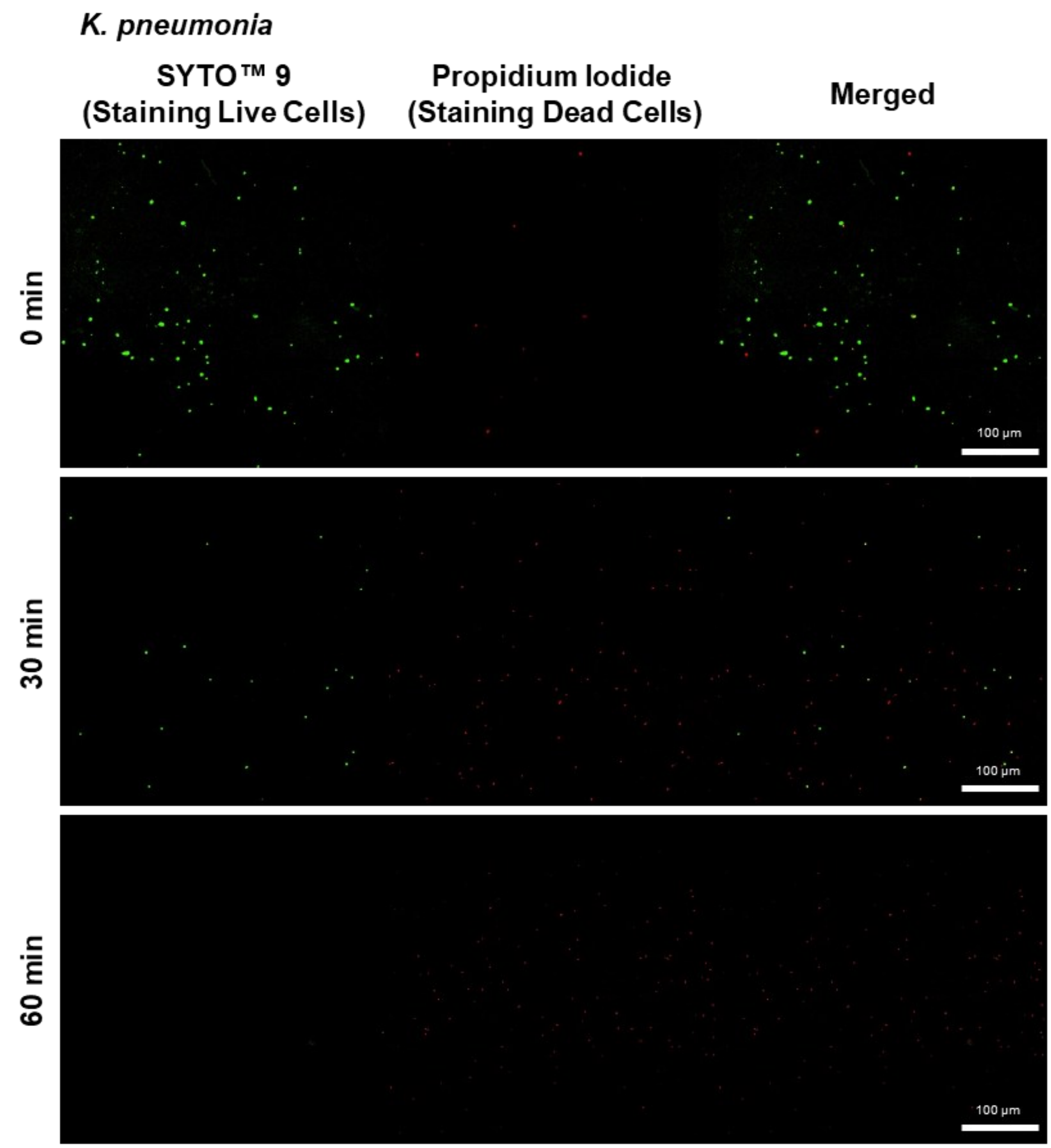

Supplementary Figure 25. Confocal fluorescence microscopy of *K. pneumoniae* stained with a LIVE/DEAD viability dye. Images were captured at 0, 30, and 60 minutes after Arcinin treatment. Scale bars = 100 µm.

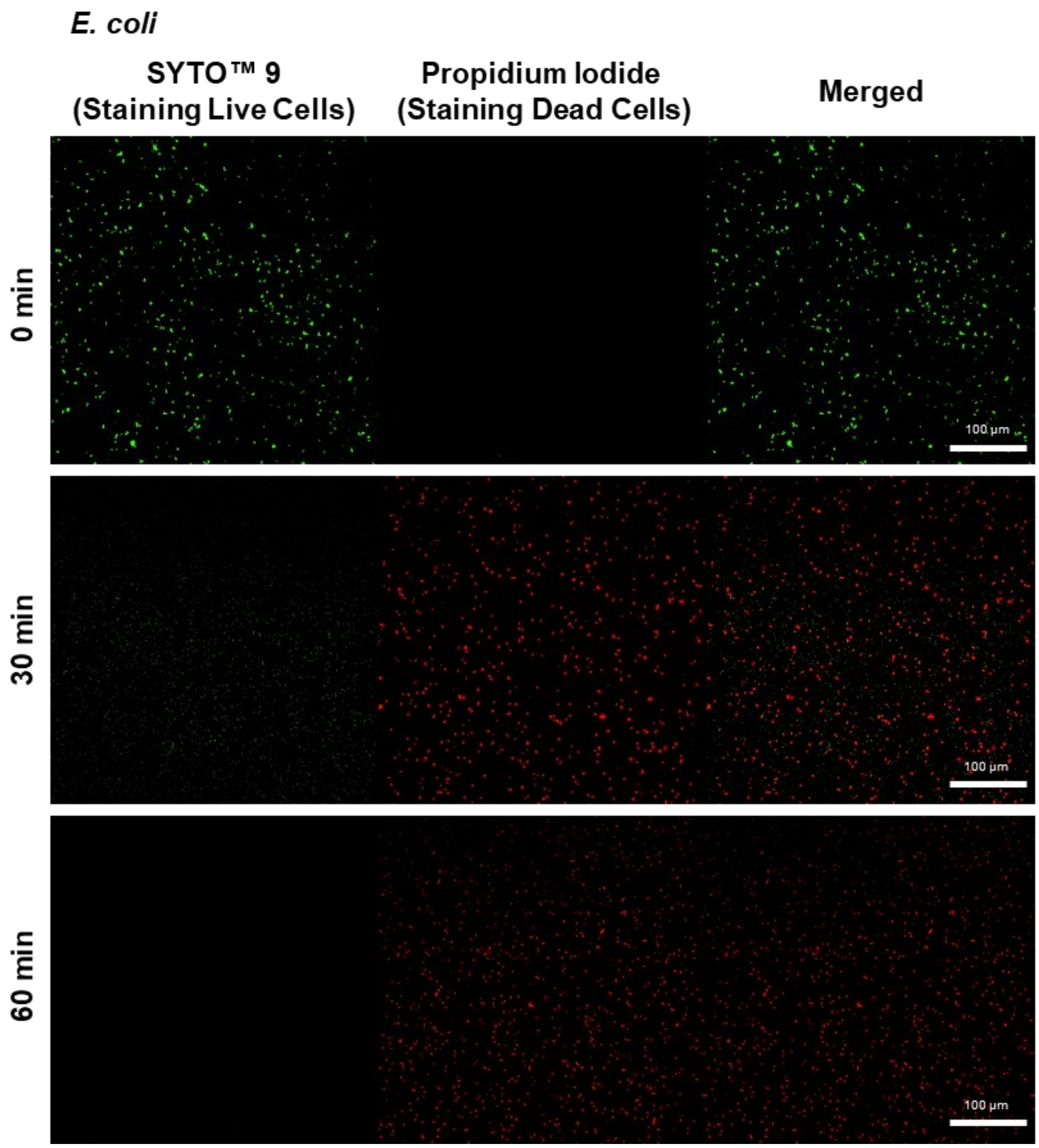

Supplementary Figure 26. Confocal fluorescence microscopy of *E. coli* stained with a LIVE/DEAD viability dye. Images were captured at 0, 30, and 60 minutes after Arcinin treatment. Scale bars = 100 µm.

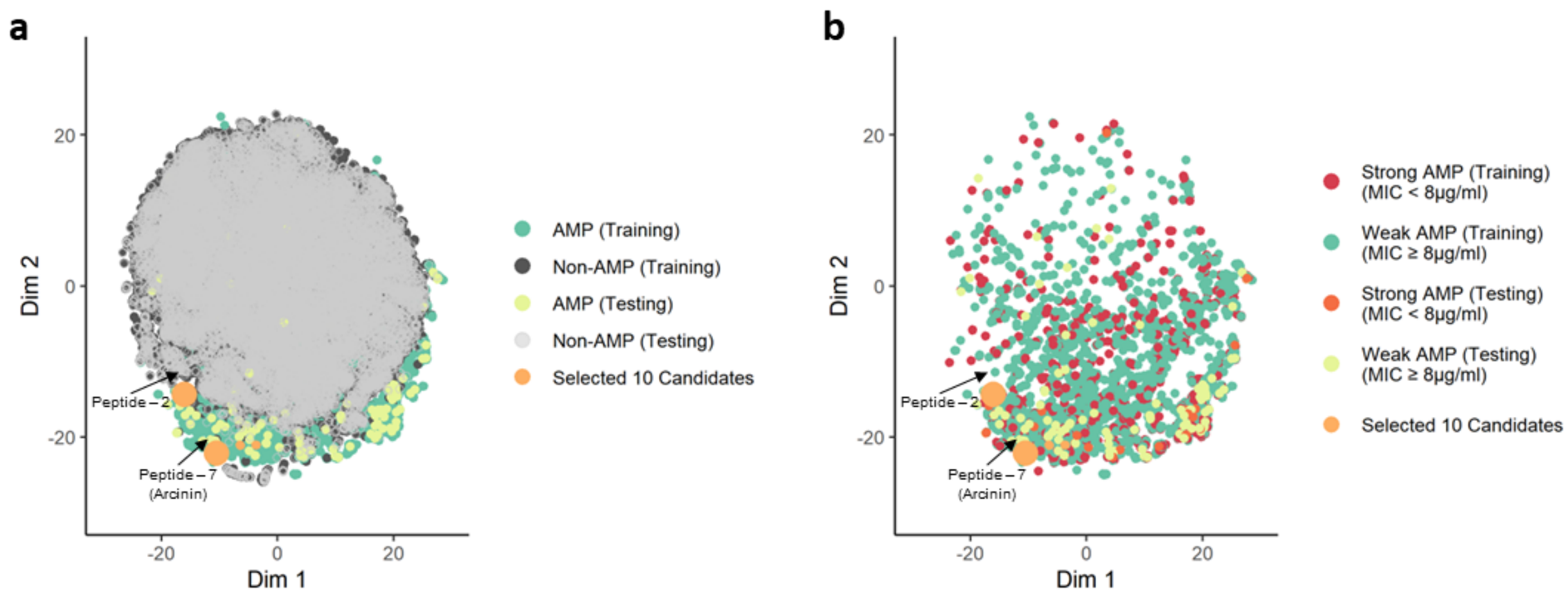

Supplementary Figure 27. t-SNE visualization of peptide sequences, including the ten screened candidates. **a.** AMPs and non-AMPs. **b.** AMPs only.

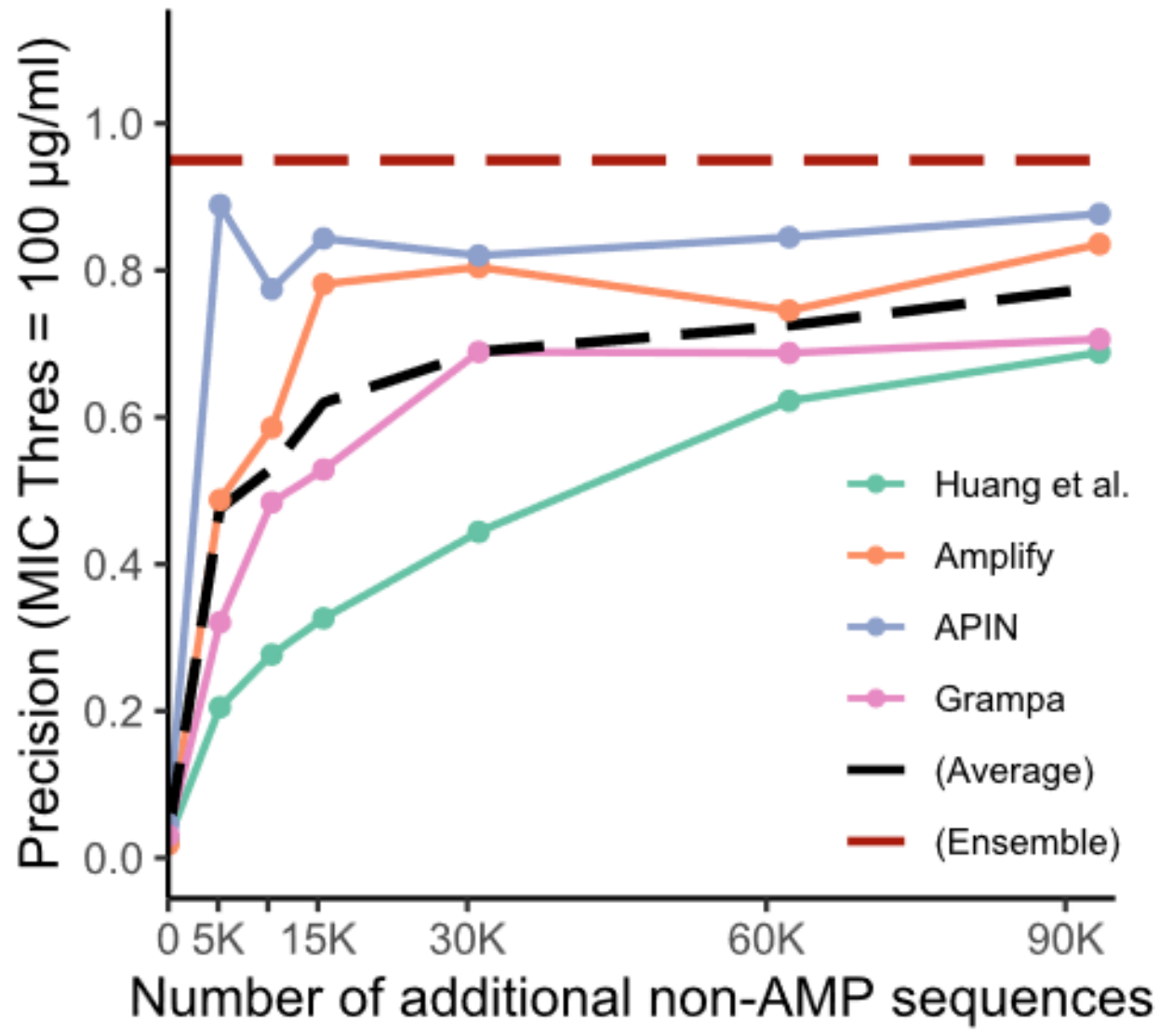

Supplementary Figure 28. Precision of four antibacterial activity prediction models trained with AMPs and varying amounts of additional non-AMPs. Precision was calculated at MIC < 100 μg/mL on the test set, and performance improved consistently as up to 90,000 non-AMPs were added.

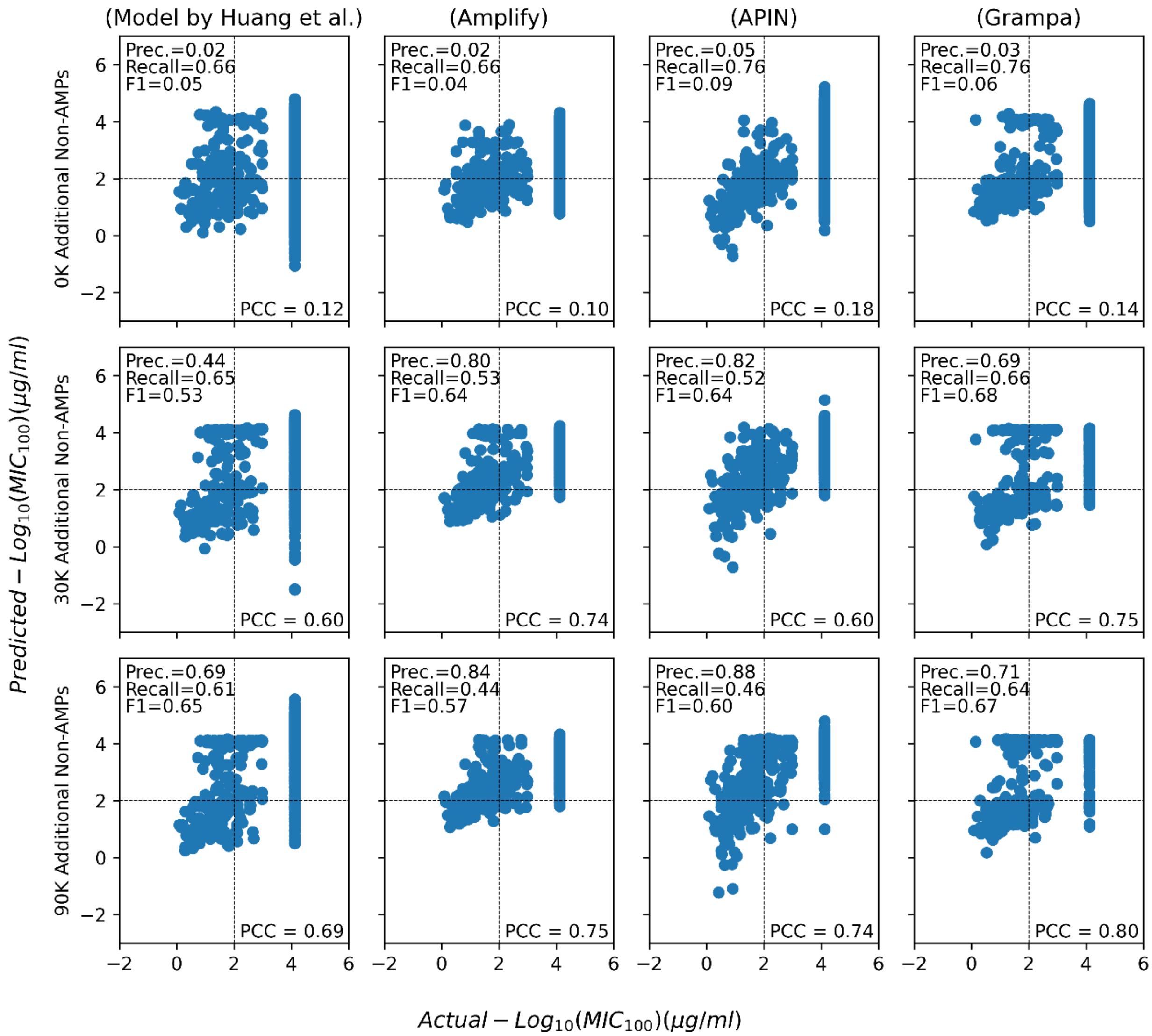

Supplementary Figure 29. Performance of four published MIC regression models (Huang, GrAMPA, APIN, AMPlify) trained with different amounts of non-AMP data (0K, 30K, 90K). Scatter plots show predicted versus actual $Log_{10}MIC$ values. Metrics include Pearson correlation coefficient (PCC), precision, recall, and F1, evaluated at MIC < 100 μg/mL.

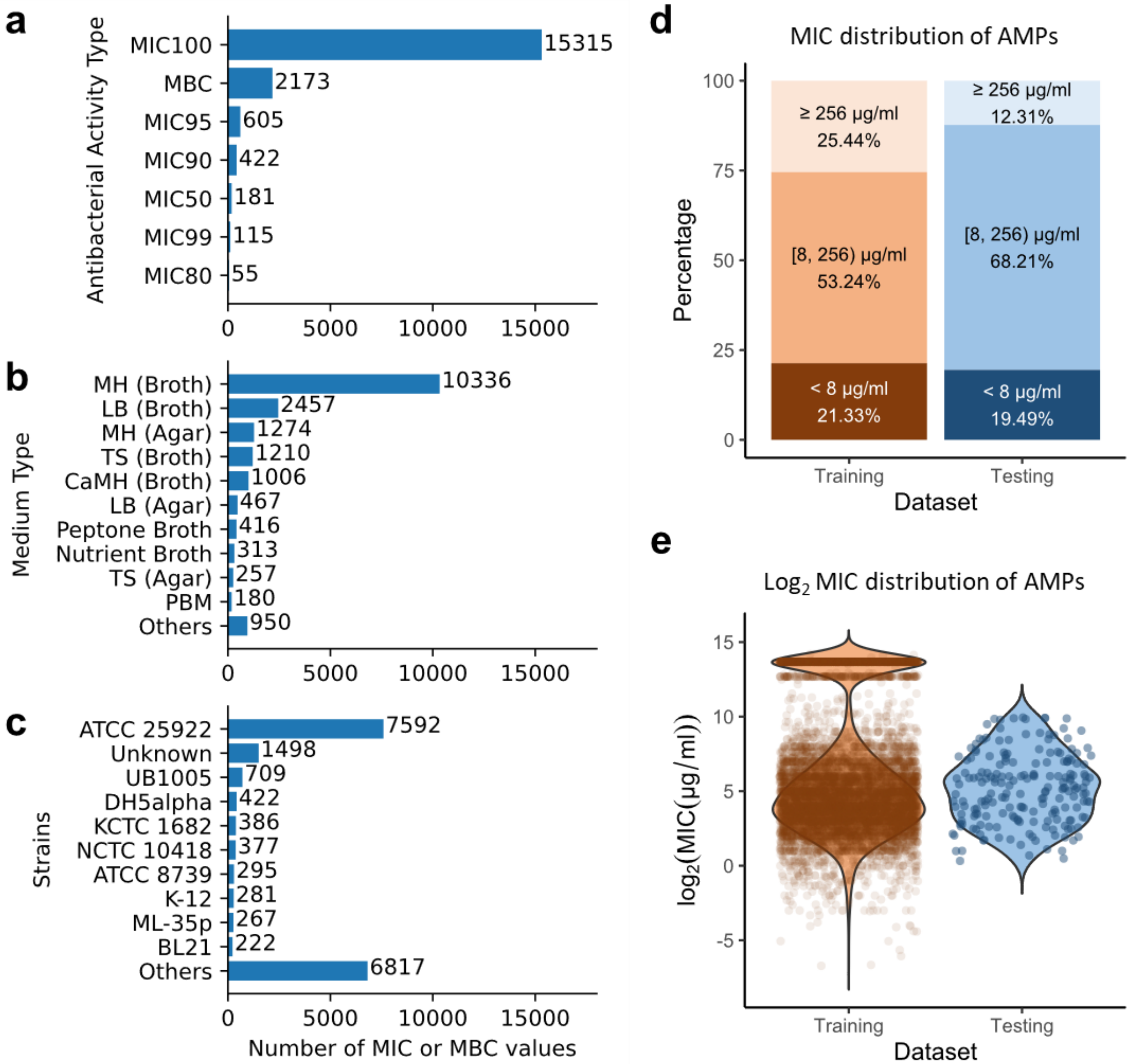

Supplementary Figure 30. Distribution of experimental conditions and activity types in the training set and the MIC distribution of AMPs in training and testing datasets. **a.** Activity measurement types. **b.** Growth media distribution. **c.** Target bacterial strains. **d.** Percentage of AMPs across three MIC ranges in both training and test datasets. **e.** The Log_2MIC values distribution of AMPs in training and testing datasets.

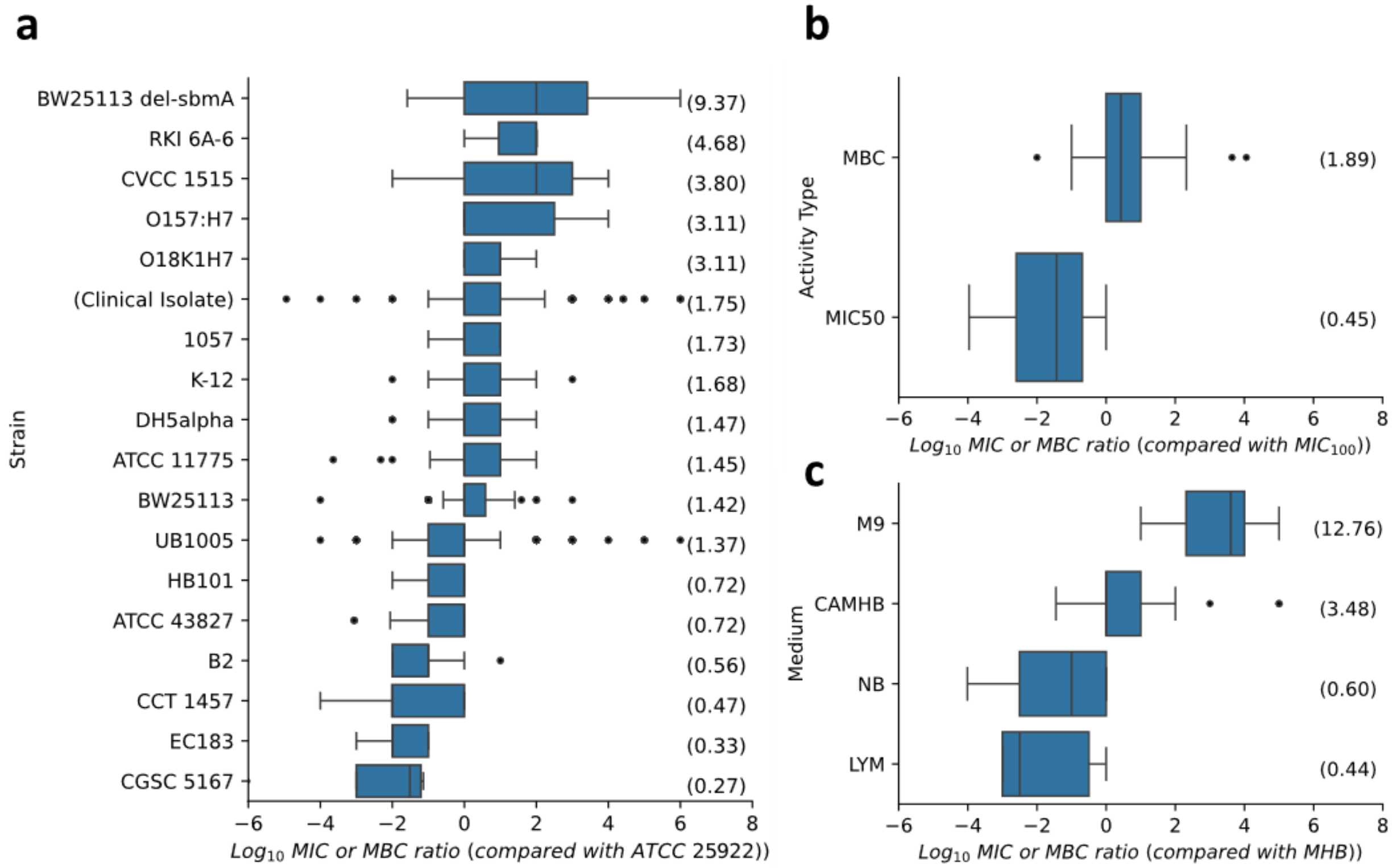

Supplementary Figure 31. Comparison of activity values under minority vs majority experimental conditions in the *E. coli* dataset. **a.** Minority strain vs. majority strain (ATCC 25922). **b.** Minority activity type vs. majority type (MIC_{100}). **c.** Minority medium vs. majority medium (MHB). The box boundaries and center correspond to Q1, Q2, and Q3, respectively, where Q1, Q2, and Q3 represent the 25th, 50th, and 75th percentiles. Whiskers extend to 1.5 × (Q3 − Q1) (Log conversion is applied before calculating the length of whiskers if applicable).

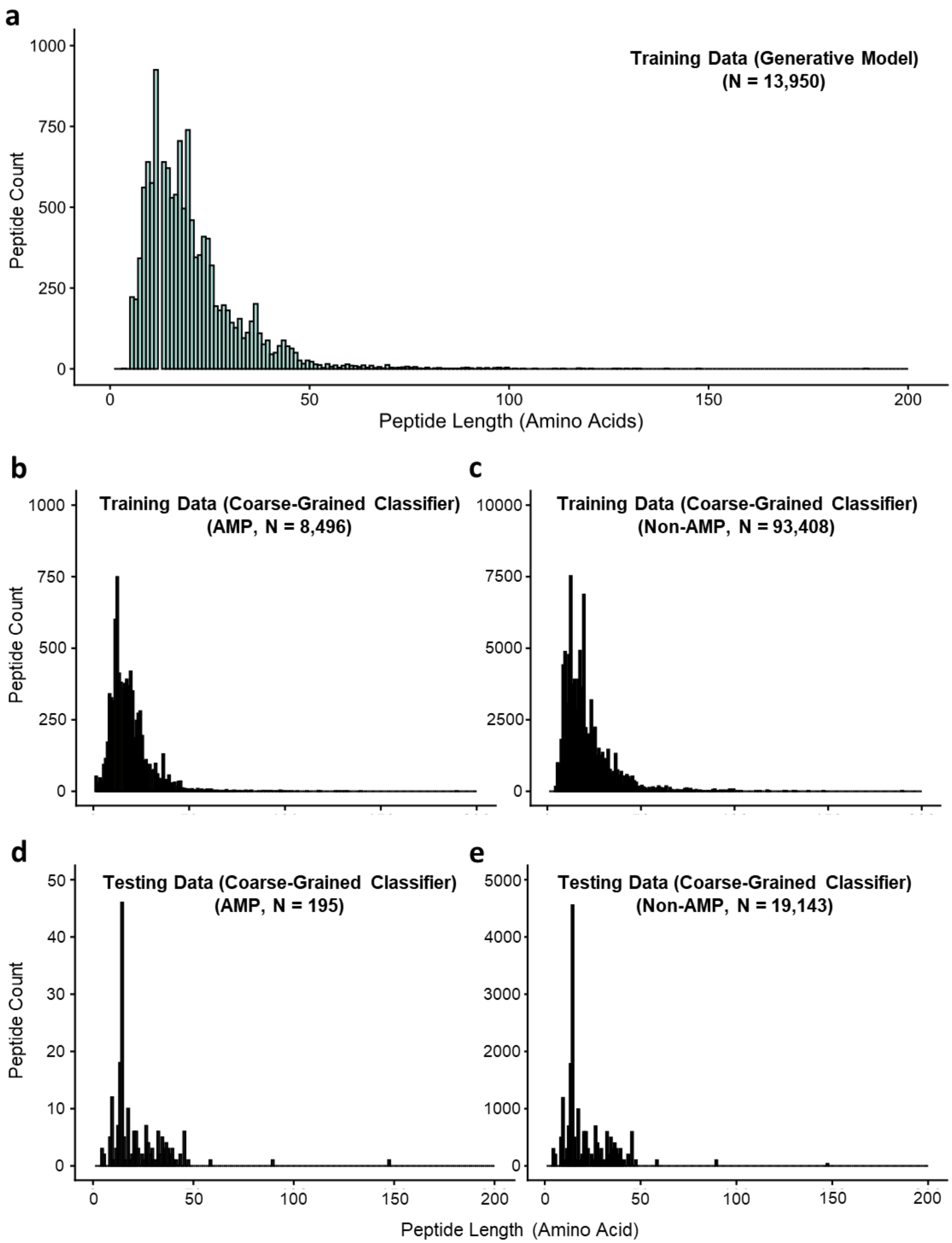

Supplementary Figure 32. Peptide-length distributions of the datasets. a. Training set of the generative model. **b.** AMP sequences in the training set of the coarse-grained

model. **c.** Non-AMP sequences in the training set of the coarse-grained model. **d.** AMP sequences in the test set of the coarse-grained model. **e.** Non-AMP sequences in the test set of the coarse-grained model.

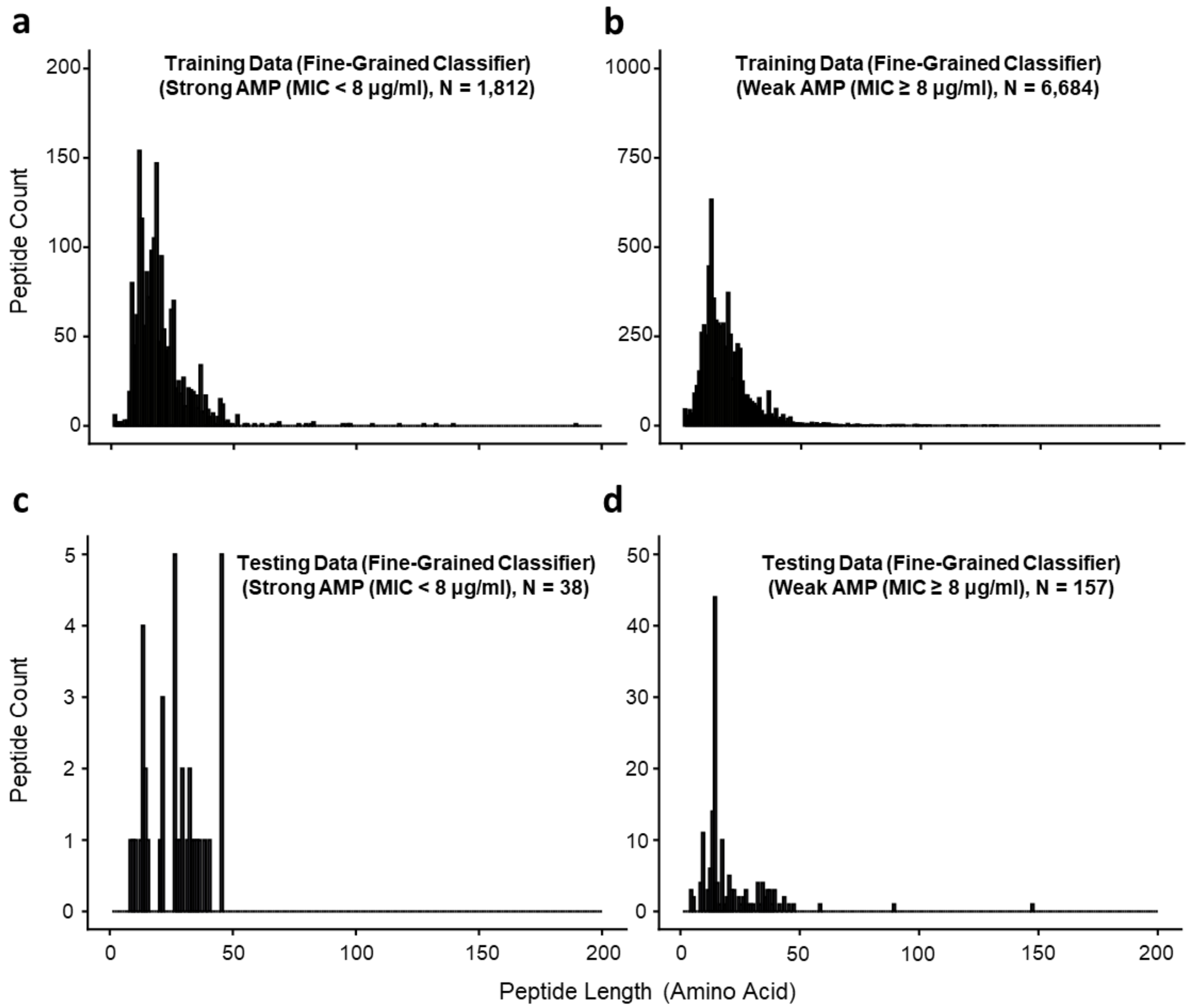

Supplementary Figure 33. Peptide-length distributions of the training and test sets for the fine-grained model. a. Strong AMP sequences (MIC < 8 µg/mL) in the training set of the fine-grained model. **b.** Weak AMP sequences (MIC ≥ 8 µg/mL) in the training set of the fine-grained model. **c.** Strong AMP sequences in the test set of the fine-grained model. **d.** Weak AMP sequences in the test set of the fine-grained model.

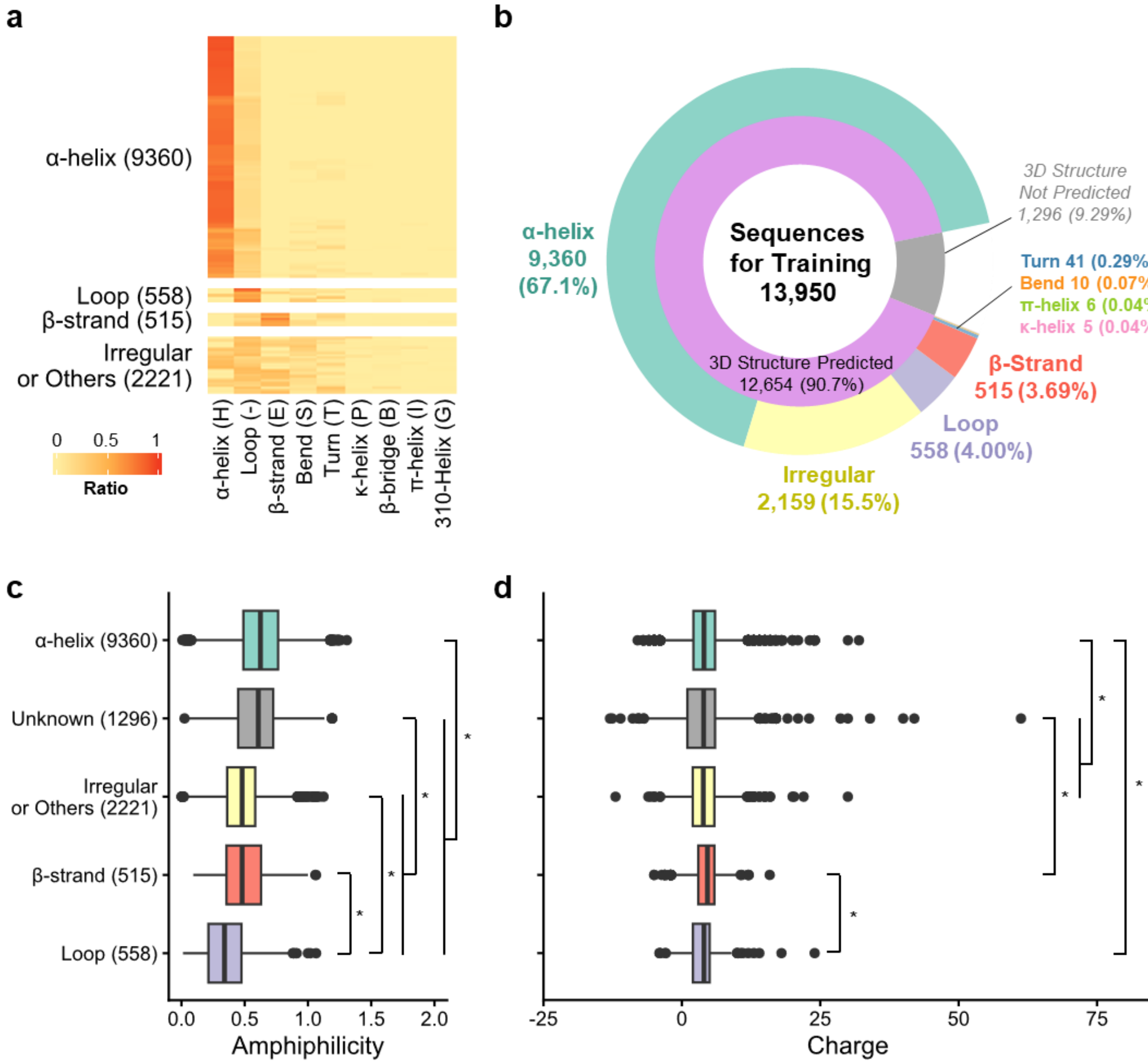

Supplementary Figure 34. Characteristics of the training data used for the generative model. a. Ratios of predicted secondary-structure classes in the sequences. **b.** Distribution of secondary-structure classes across sequences. **c.** Amphiphilicity of sequences with different predicted secondary structures. **d.** Charge of sequences with different predicted secondary structures.

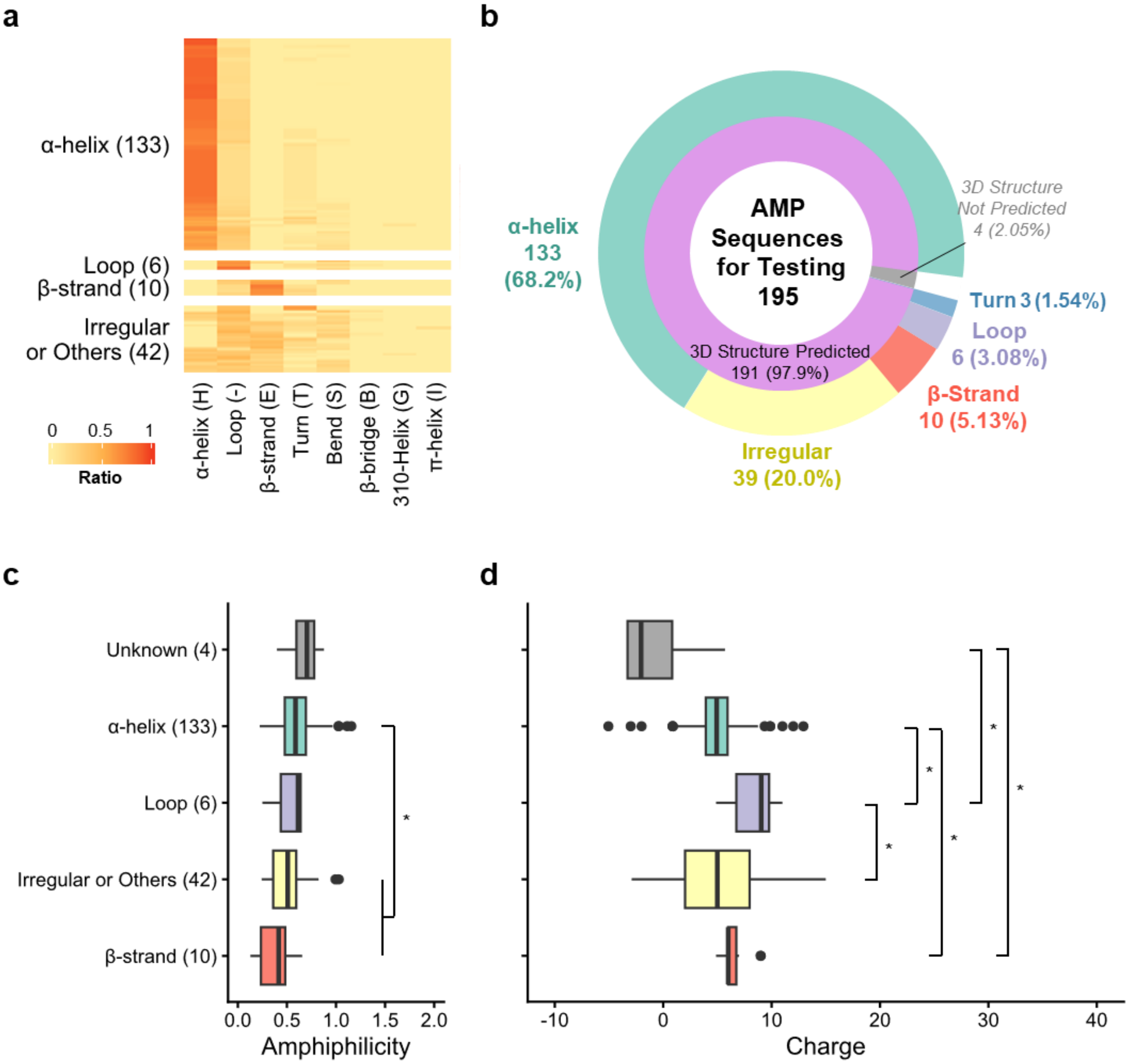

Supplementary Figure 35. Characteristics of the test-set data used for the classifiers (AMP sequences only). a. Ratios of predicted secondary-structure classes in the sequences. **b.** Distribution of secondary-structure classes across sequences. **c.** Amphiphilicity of sequences with different predicted secondary structures. **d.** Charge of sequences with different predicted secondary structures.

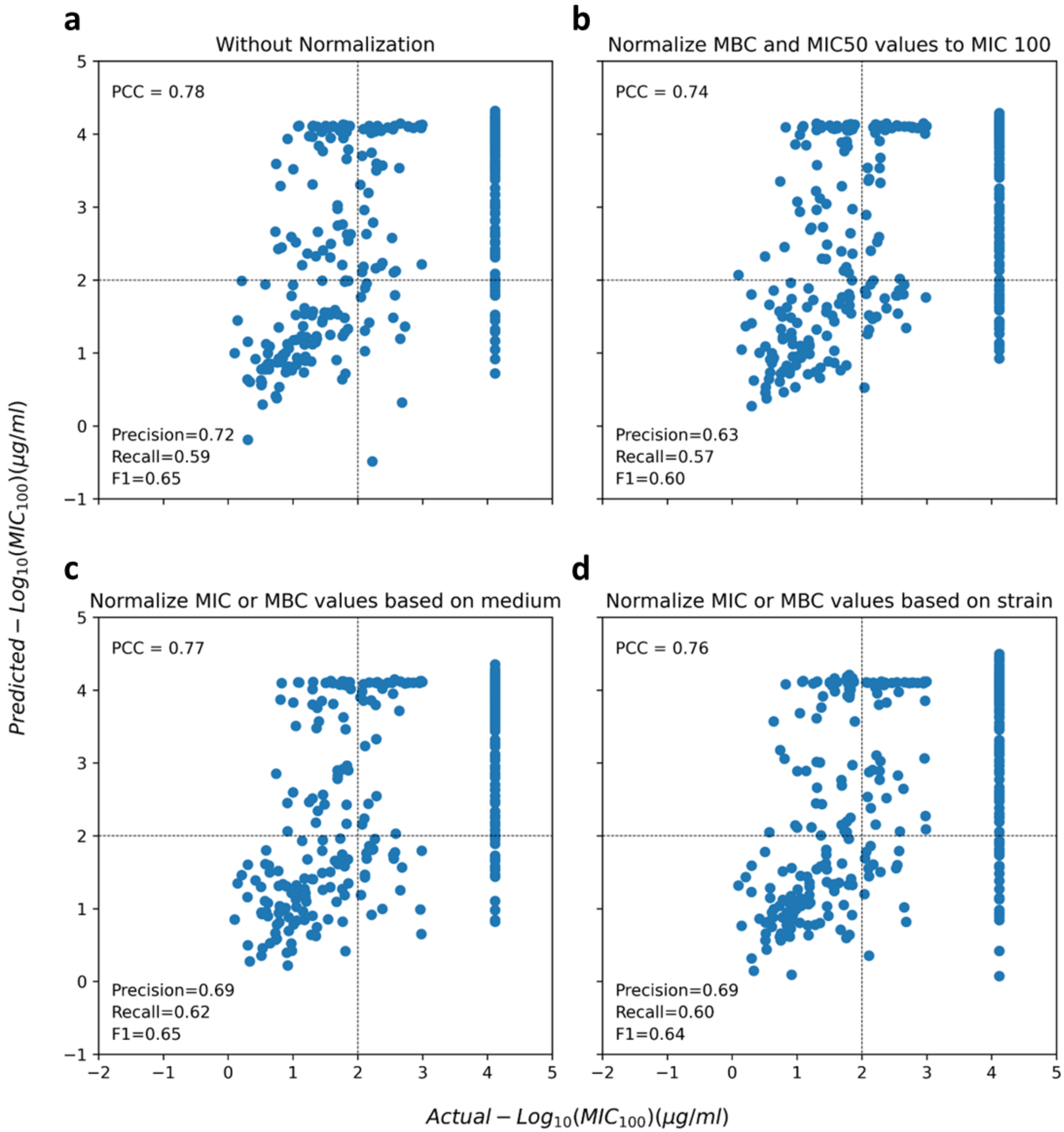

Supplementary Figure 36. Effect of data normalization on MIC regression performance using the Huang model (trained with 93,408 augmented non-AMPs). Scatter plots show predicted versus actual Log_{10}MIC and associated metrics under: **a.** no normalization; **b.** MIC50 and MBC normalized to MIC100; **c.** MIC and MBC normalized by medium; **d.** MIC and MBC normalized by *E. coli* strain.

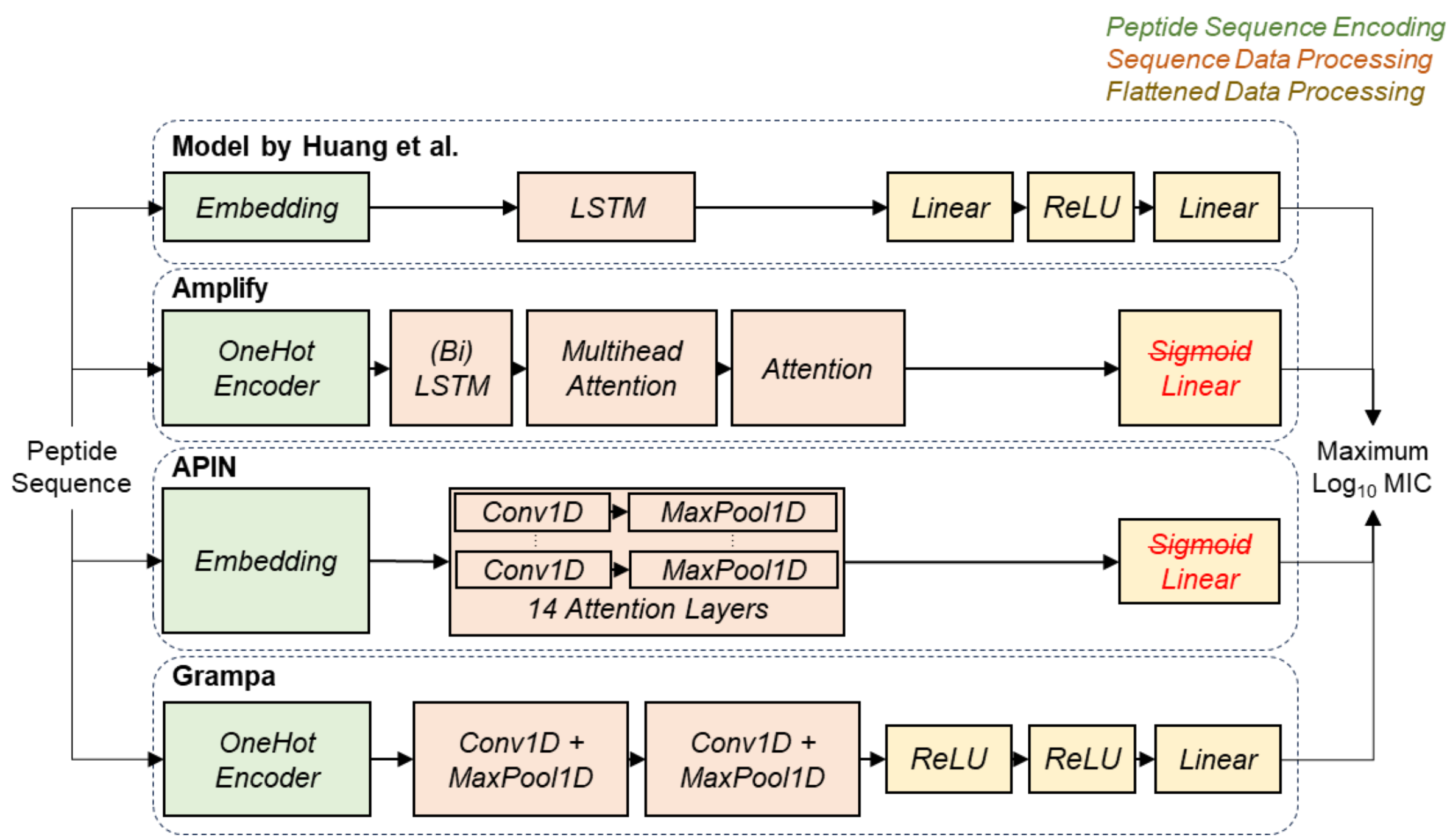

Supplementary Figure 37. Architecture of the ensemble MIC regression model. The ensemble comprises four published models, with APIN and AMPlify converted from binary classifiers to regression models. The ensemble output is defined as the maximum predicted Log_{10} MIC among the four models.

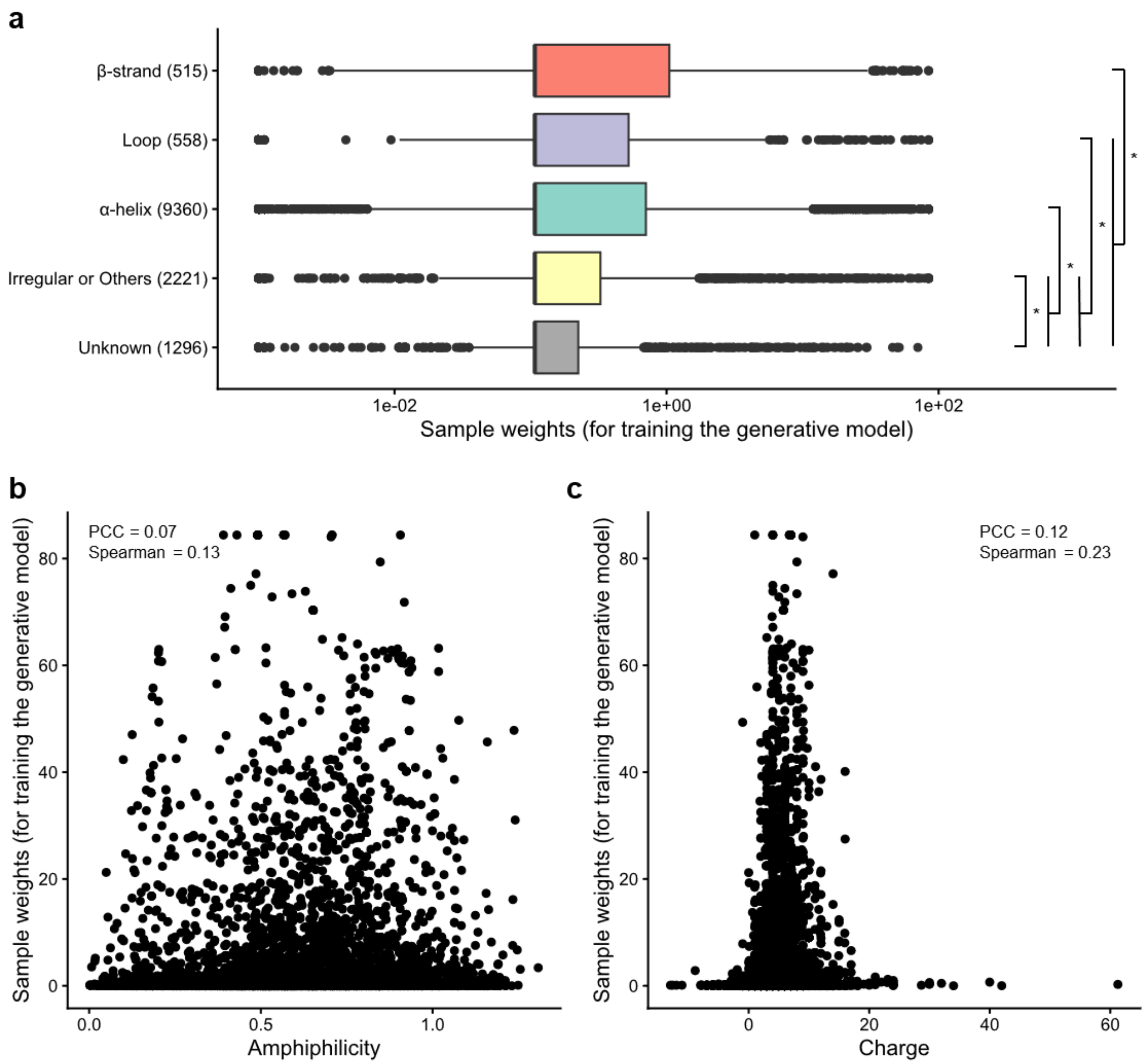

Supplementary Figure 38. Sample weights for training the generative model across sequences with different properties. a. Sample weights of sequences with different predicted secondary structures. The box boundaries and center correspond to Q1, Q2, and Q3, respectively, where Q1, Q2, and Q3 represent the 25th, 50th, and 75th percentiles. Whiskers extend to 1.5 × (Q3 − Q1). Log conversion is applied before calculating the length of whiskers. **b.** Sample weights of sequences plotted against amphiphilicity. **c.** Sample weights of sequences plotted against charge.

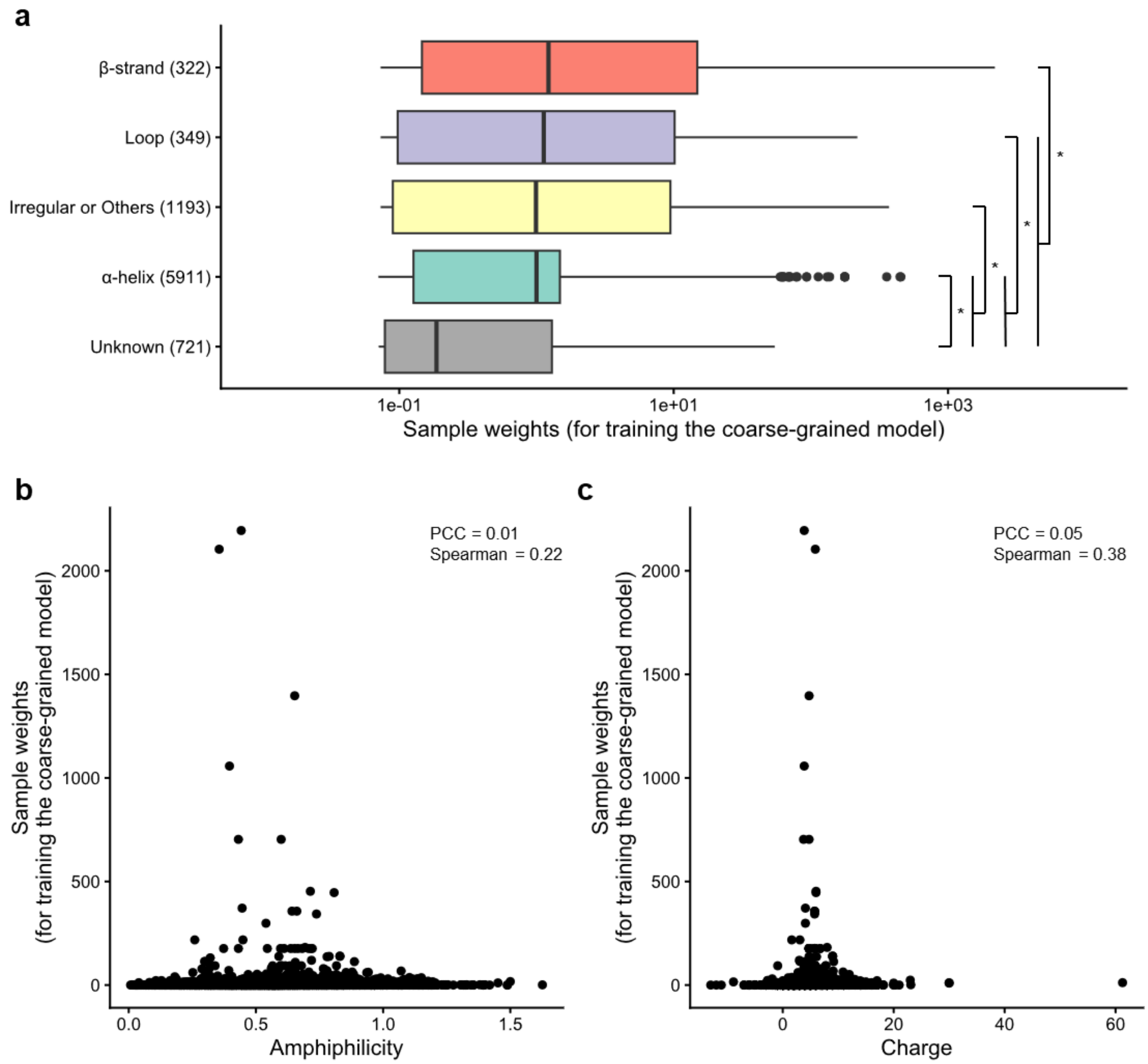

Supplementary Figure 39. Sample weights for training the coarse-grained classifier across sequences with different properties. a. Sample weights of sequences with different predicted secondary structures. The box boundaries and center correspond to Q1, Q2, and Q3, respectively, where Q1, Q2, and Q3 represent the 25th, 50th, and 75th percentiles. Whiskers extend to 1.5 × (Q3 − Q1). Log conversion is applied before calculating the length of whiskers. **b.** Sample weights of

sequences plotted against amphiphilicity. **c.** Sample weights of sequences plotted against charge.

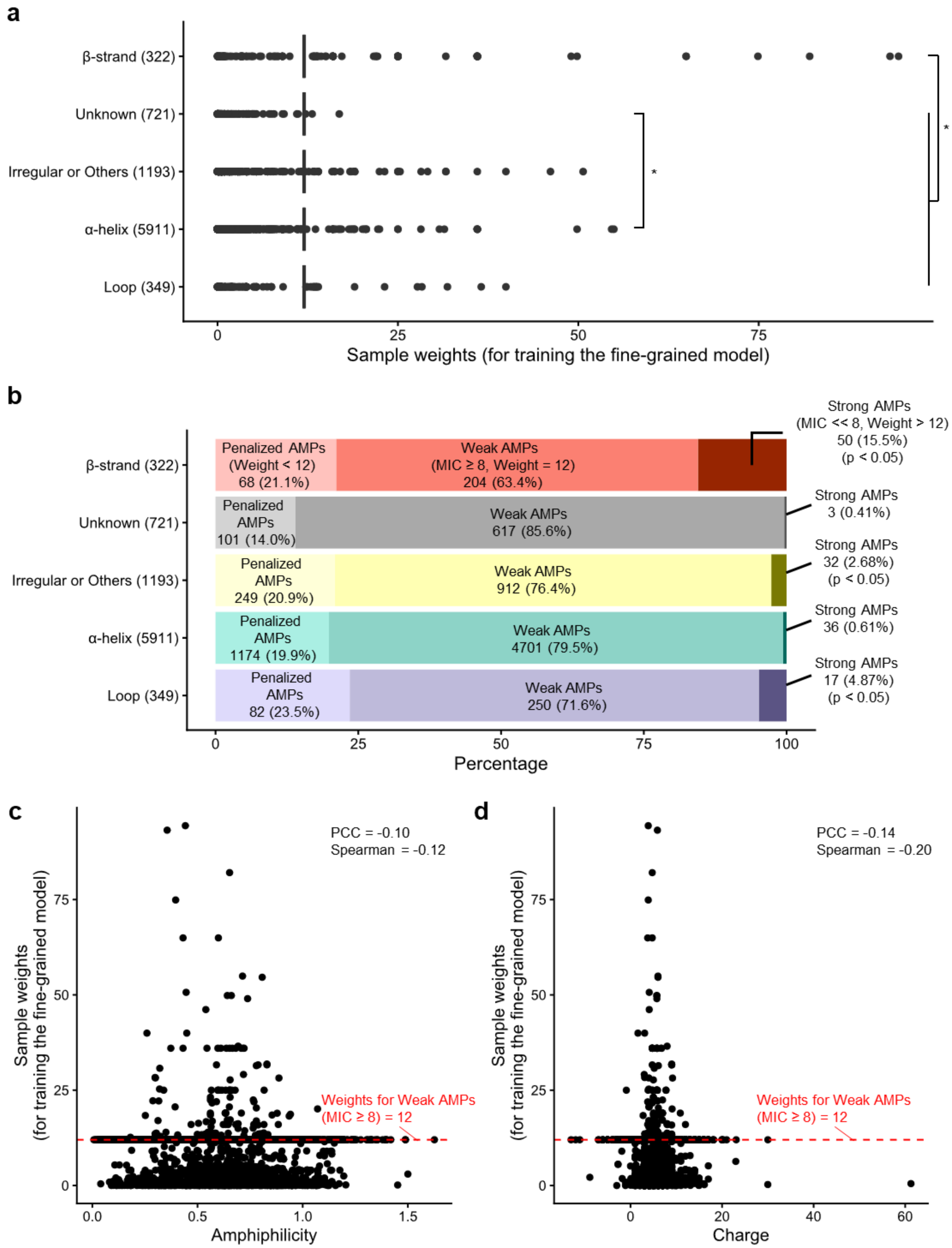
a
β-strand (322)
Unknown (721)
Irregular or Others (1193)
α-helix (5911)
Loop (349)
*
*
0
25
50
75
Sample weights (for training the fine-grained model)
b
Strong AMPs
(MIC << 8, Weight > 12)
50 (15.5%)
(p < 0.05)
Penalized AMPs
(Weight < 12)
68 (21.1%)
Weak AMPs
(MIC ≥ 8, Weight = 12)
204 (63.4%)
Penalized
AMPs
101 (14.0%)
Weak AMPs
617 (85.6%)
Strong AMPs
3 (0.41%)
Penalized
AMPs
249 (20.9%)
Weak AMPs
912 (76.4%)
Strong AMPs
32 (2.68%)
(p < 0.05)
Penalized
AMPs
1174 (19.9%)
Weak AMPs
4701 (79.5%)
Strong AMPs
36 (0.61%)
Penalized
AMPs
82 (23.5%)
Weak AMPs
250 (71.6%)
Strong AMPs
17 (4.87%)
(p < 0.05)
0
25
50
75
100
Percentage
c
Sample weights
(for training the fine-grained model)
PCC = -0.10
Spearman = -0.12
Weights for Weak AMPs
(MIC ≥ 8) = 12
0
25
50
75
0.0
0.5
1.0
1.5
Amphiphilicity
d
Sample weights
(for training the fine-grained model)
PCC = -0.14
Spearman = -0.20
Weights for Weak AMPs
(MIC ≥ 8) = 12
0
25
50
75
0
20
40
60
Charge

Supplementary Figure 40. Sample weights for training the fine-grained classifier across sequences with different properties. a. Sample weights of sequences with different predicted secondary structures. The box boundaries and center correspond to Q1, Q2, and Q3, respectively, where Q1, Q2, and Q3 represent the 25th, 50th, and 75th percentiles. Whiskers extend to $1.5 \times (Q3 - Q1)$. Log conversion is applied before calculating the length of whiskers. **b.** Sample weights binned by the specific weight value (= 12) for sequences with different predicted secondary structures. **c.** Sample weights of sequences plotted against amphiphilicity. **d.** Sample weights of sequences plotted against charge.

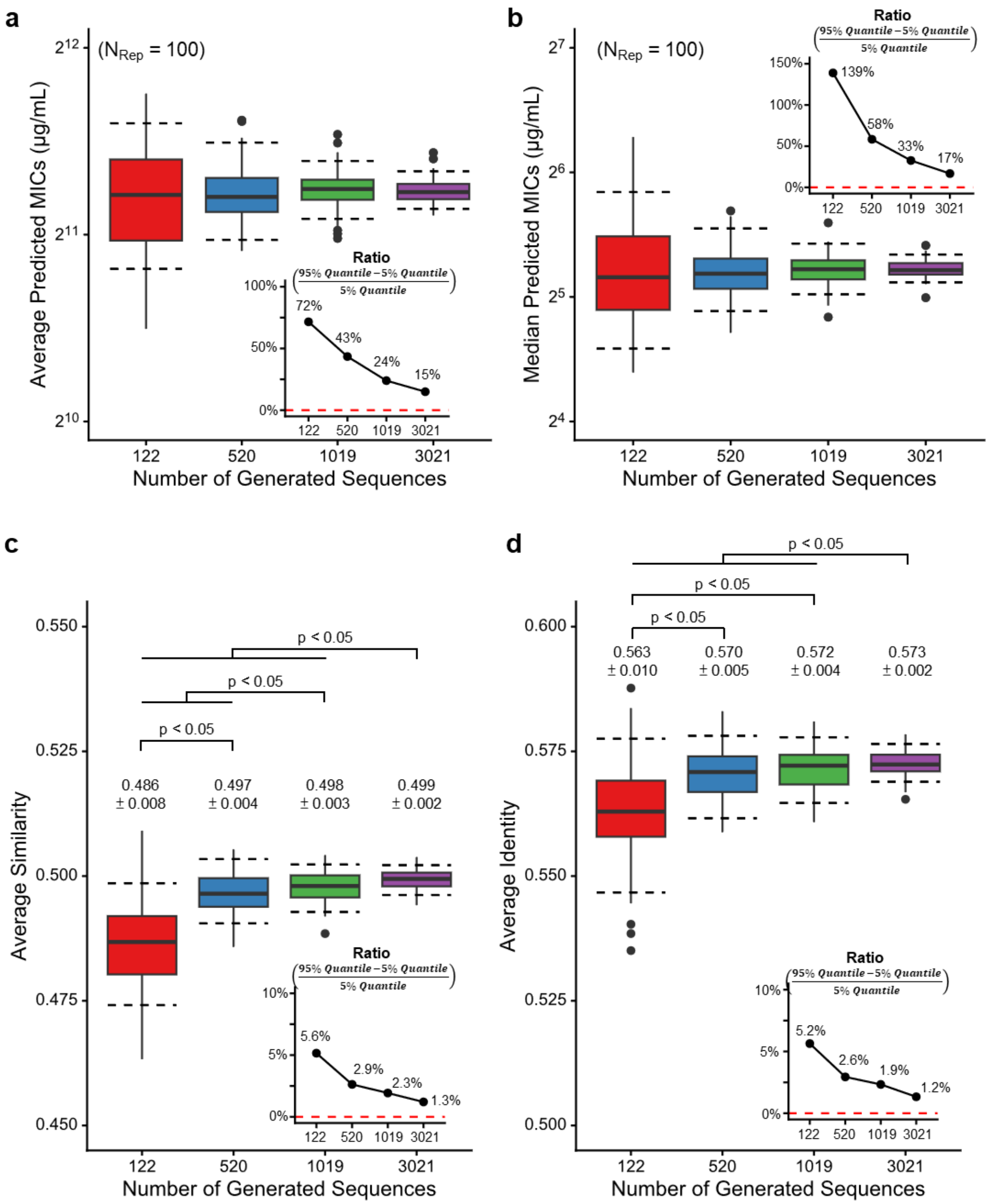

Supplementary Figure 41. Distribution of average generative-model performance (predicted MIC, similarity, and identity) across different numbers of generated sequences over 100 replicates. Median predicted MIC values are also included. The box boundaries and center correspond to Q1, Q2, and Q3, respectively, where Q1, Q2,

and Q3 represent the 25th, 50th, and 75th percentiles. Whiskers extend to 1.5 × (Q3 − Q1) (Log conversion is applied before calculating the length of whiskers if applicable). **a.** Average predicted MICs. **b.** Median predicted MICs. **c.** Average similarity. **d.** Average identity.

References

1. Pirtskhalava, M. *et al.* DBAASP v3: database of antimicrobial/cytotoxic activity and structure of peptides as a resource for development of new therapeutics. *Nucleic Acids Research* **49**, D288–D297 (2021).
2. Gawde, U. *et al.* CAMPR4: a database of natural and synthetic antimicrobial peptides. *Nucleic Acids Research* **51**, D377–D383 (2023).
3. Jhong, J.-H. *et al.* dbAMP 2.0: updated resource for antimicrobial peptides with an enhanced scanning method for genomic and proteomic data. *Nucleic Acids Research* **50**, D460–D470 (2022).
4. Shi, G. *et al.* DRAMP 3.0: an enhanced comprehensive data repository of antimicrobial peptides. *Nucleic Acids Research* **50**, D488–D496 (2022).
5. Wang, G., Li, X. & Wang, Z. APD3: the antimicrobial peptide database as a tool for research and education. *Nucleic Acids Research* **44**, D1087–D1093 (2016).
6. Bray, T. *The Javascript Object Notation (Json) Data Interchange Format*. (2014).
7. Santajit, S. & Indrawattana, N. Mechanisms of Antimicrobial Resistance in ESKAPE Pathogens. *Biomed Res Int* **2016**, 2475067 (2016).
8. Levitsky, L. I., Klein, J. A., Ivanov, M. V. & Gorshkov, M. V. Pyteomics 4.0: Five Years of Development of a Python Proteomics Framework. *J. Proteome Res.* **18**, 709–714 (2019).
9. Mueller, J. H. & Hinton, J. A Protein-Free Medium for Primary Isolation of the Gonococcus and Meningococcus. *Proceedings of the Society for Experimental Biology and Medicine* **48**, 330–333 (1941).
10. Wikler, M. A. Methods for dilution antimicrobial susceptibility tests for bacteria that grow aerobically: approved standard. *Clsi (Nccls)* **26**, (2006).

11. Raggett, D., Le Hors, A., Jacobs, I., & others. HTML 4.01 Specification. *W3C recommendation* **24**, (1999).
12. Lamiable, A. *et al.* PEP-FOLD3: faster de novo structure prediction for linear peptides in solution and in complex. *Nucleic Acids Research* **44**, W449–W454 (2016).
13. Rey, J., Murail, S., de Vries, S., Derreumaux, P. & Tuffery, P. PEP-FOLD4: a pH-dependent force field for peptide structure prediction in aqueous solution. *Nucleic Acids Research* **51**, W432–W437 (2023).
14. Kabsch, W. & Sander, C. Dictionary of protein secondary structure: Pattern recognition of hydrogen-bonded and geometrical features. *Biopolymers* **22**, 2577–2637 (1983).
15. Hekkelman, M. L., Salmoral, D. Á., Perrakis, A. & Joosten, R. P. DSSP 4: FAIR annotation of protein secondary structure. *Protein Science* **34**, e70208 (2025).
16. Fingerhut, L. C. H. W., Miller, D. J., Strugnell, J. M., Daly, N. L. & Cooke, I. R. ampir: an R package for fast genome-wide prediction of antimicrobial peptides. *Bioinformatics* **36**, 5262–5263 (2021).
17. Hochreiter, S. & Schmidhuber, J. LSTM can solve hard long time lag problems. *Advances in neural information processing systems* **9**, (1996).
18. Huang, J. *et al.* Identification of potent antimicrobial peptides via a machine-learning pipeline that mines the entire space of peptide sequences. *Nat. Biomed. Eng* **7**, 797–810 (2023).
19. Su, X., Xu, J., Yin, Y., Quan, X. & Zhang, H. Antimicrobial peptide identification using multi-scale convolutional network. *BMC Bioinformatics* **20**, 730 (2019).

20. Li, C. *et al.* AMPlify: attentive deep learning model for discovery of novel antimicrobial peptides effective against WHO priority pathogens. *BMC Genomics* **23**, 77 (2022).
21. Witten, J. & Witten, Z. Deep learning regression model for antimicrobial peptide design. 692681 Preprint at https://doi.org/10.1101/692681 (2019).
22. Muller, P. Y. & Milton, M. N. The determination and interpretation of the therapeutic index in drug development. *Nat Rev Drug Discov* **11**, 751–761 (2012).
23. Finkel, R., Clark, M. A. & Cubeddu, L. X. *Pharmacology*. (Lippincott Williams & Wilkins, 2009).
24. Alamdari, S. *et al.* Protein generation with evolutionary diffusion: sequence is all you need. 2023.09.11.556673 Preprint at https://doi.org/10.1101/2023.09.11.556673 (2024).
25. Austin, J., Johnson, D. D., Ho, J., Tarlow, D. & van den Berg, R. Structured Denoising Diffusion Models in Discrete State-Spaces. in *Advances in Neural Information Processing Systems* (eds Ranzato, M., Beygelzimer, A., Dauphin, Y., Liang, P. S. & Vaughan, J. W.) vol. 34 17981–17993 (Curran Associates, Inc., 2021).
26. Dynkin, E. B. Markov processes. in *Markov Processes: Volume 1* 77–104 (Springer, 1965).
27. Sohl-Dickstein, J., Weiss, E., Maheswaranathan, N. & Ganguli, S. Deep unsupervised learning using nonequilibrium thermodynamics. in *International conference on machine learning* 2256–2265 (pmlr, 2015).
28. Ho, J., Jain, A. & Abbeel, P. Denoising diffusion probabilistic models. *Advances in neural information processing systems* **33**, 6840–6851 (2020).

29. Hoogeboom, E., Nielsen, D., Jaini, P., Forré, P. & Welling, M. Argmax flows and multinomial diffusion: Towards non-autoregressive language models. *arXiv preprint arXiv:2102.05379* **3**, 5 (2021).

30. Kalchbrenner, N. *et al.* Neural machine translation in linear time. *arXiv preprint arXiv:1610.10099* (2016).

31. Suzek, B. E. *et al.* UniRef clusters: a comprehensive and scalable alternative for improving sequence similarity searches. *Bioinformatics* **31**, 926–932 (2015).

32. Needleman, S. B. & Wunsch, C. D. A general method applicable to the search for similarities in the amino acid sequence of two proteins. *Journal of Molecular Biology* **48**, 443–453 (1970).

33. Henikoff, S. & Henikoff, J. G. Amino acid substitution matrices from protein blocks. *Proceedings of the National Academy of Sciences* **89**, 10915–10919 (1992).

34. Lin, Z. *et al.* Evolutionary-scale prediction of atomic-level protein structure with a language model. *Science* **379**, 1123–1130 (2023).

35. Li, Y. *et al.* BroadAMP-GPT: AI-Driven generation of broad-spectrum antimicrobial peptides for combating multidrug-resistant ESKAPE pathogens. *Gut Microbes* **17**, 2523811.

36. Wang, Y. *et al.* Artificial intelligence using a latent diffusion model enables the generation of diverse and potent antimicrobial peptides. *Science Advances* **11**, eadp7171 (2025).

37. esunAI/FlowAMP · Hugging Face. https://huggingface.co/esunAI/FlowAMP.

38. Han, J., Kong, T. & Liu, J. PepNet: an interpretable neural network for anti-inflammatory and antimicrobial peptides prediction using a pre-trained protein language model. *Commun Biol* **7**, 1–13 (2024).

39. Wang, R. *et al.* Diff-AMP: tailored designed antimicrobial peptide framework with all-in-one generation, identification, prediction and optimization. *Briefings in Bioinformatics* **25**, bbae078 (2024).

40. Elnaggar, A. *et al.* ProtTrans: Toward Understanding the Language of Life Through Self-Supervised Learning. *IEEE Trans Pattern Anal Mach Intell* **44**, 7112–7127 (2022).

41. Davison, A. C. & Hinkley, D. V. *Bootstrap Methods and Their Application*. (Cambridge university press, 1997).
