# Supplementary material for: Discovery of potent low-toxicity antimicrobial peptides through diffusion modeling
Source: Nat Commun. 2026 Jul 7;17:8396. doi: 10.1038/s41467-026-75030-8 (PMC13473622; doi:10.1038/s41467-026-75030-8)
Supplement: Supplementary file 2 [file 41467_2026_75030_MOESM2_ESM.pdf]

nature portfolio

Corresponding author(s): Ilias Tagkopoulos

Last updated by author(s): 2026/06/01

Reporting Summary

Nature Portfolio wishes to improve the reproducibility of the work that we publish. This form provides structure for consistency and transparency in reporting. For further information on Nature Portfolio policies, see our Editorial Policies and the Editorial Policy Checklist.

Statistics

For all statistical analyses, confirm that the following items are present in the figure legend, table legend, main text, or Methods section.

n/a	Confirmed	
[ ]	[x]	The exact sample size (*n*) for each experimental group/condition, given as a discrete number and unit of measurement
[ ]	[x]	A statement on whether measurements were taken from distinct samples or whether the same sample was measured repeatedly
[ ]	[x]	The statistical test(s) used AND whether they are one- or two-sided<br>*Only common tests should be described solely by name; describe more complex techniques in the Methods section.*
[x]	[ ]	A description of all covariates tested
[ ]	[x]	A description of any assumptions or corrections, such as tests of normality and adjustment for multiple comparisons
[ ]	[x]	A full description of the statistical parameters including central tendency (e.g. means) or other basic estimates (e.g. regression coefficient) AND variation (e.g. standard deviation) or associated estimates of uncertainty (e.g. confidence intervals)
[ ]	[x]	For null hypothesis testing, the test statistic (e.g. *F*, *t*, *r*) with confidence intervals, effect sizes, degrees of freedom and *P* value noted<br>*Give P values as exact values whenever suitable.*
[x]	[ ]	For Bayesian analysis, information on the choice of priors and Markov chain Monte Carlo settings
[x]	[ ]	For hierarchical and complex designs, identification of the appropriate level for tests and full reporting of outcomes
[ ]	[x]	Estimates of effect sizes (e.g. Cohen's *d*, Pearson's *r*), indicating how they were calculated

Our web collection on statistics for biologists contains articles on many of the points above.

Software and code

Policy information about availability of computer code

Data collection	BioTek Synergy H1 and Synergy HTX (Gen5 software) microplate readers, Zeiss LSM 980 with Airyscan 2 (confocal microscopy), ThermoFisher Quattro S (SEM), TFS Glacios (Cryo-TEM), ImageJ (wound area quantification)
Data analysis	SigmaPlot (statistical analysis), Microsoft Excel, ImageJ (wound area quantification), AAT Bioquest LD50 calculator (hemolysis LC50 fitting), R (statistical analysis and figure generation), python (statistical analysis and figure generation), PEPFOLD4 (peptide 3D structure prediction), CHARMM-GUI (molecular dynamics configuration), Gromacs (2024.03, molecular dynamics simulation)

For manuscripts utilizing custom algorithms or software that are central to the research but not yet described in published literature, software must be made available to editors and reviewers. We strongly encourage code deposition in a community repository (e.g. GitHub). See the Nature Portfolio guidelines for submitting code & software for further information.

Data

Policy information about availability of data

All manuscripts must include a data availability statement. This statement should provide the following information, where applicable:

- Accession codes, unique identifiers, or web links for publicly available datasets
- A description of any restrictions on data availability
- For clinical datasets or third party data, please ensure that the statement adheres to our policy

The training and testing data of the models in this study were deposited in the corresponding GitHub repository: [https://github.com/IBPA/ARCADIAMP]. All the processed data shown in the Figures and Tables are available in the source data files. The protein structures used in this study are available in the PDB database

under accession codes 2MLT [https://www.rcsb.org/structure/2MLT] and 7CSS [https://www.rcsb.org/structure/7CSS].
Publicly available databases used for peptide sequences and structural analysis include DBAASP (https://dbaasp.org), CAMPR4 (http://www.camp.bicnirrh.res.in), UniProt (https://www.uniprot.org), and the Protein Data Bank (PDB; https://www.rcsb.org). Source data for the figures and tables are provided with this paper. Any other raw data, including microscopy images and simulation trajectories, are available from the corresponding author upon request.

Research involving human participants, their data, or biological material

Policy information about studies with human participants or human data. See also policy information about sex, gender (identity/presentation), and sexual orientation and race, ethnicity and racism.

Reporting on sex and gender	Not applicable
Reporting on race, ethnicity, or other socially relevant groupings	Not applicable
Population characteristics	Not applicable
Recruitment	Not applicable
Ethics oversight	Not applicable

Note that full information on the approval of the study protocol must also be provided in the manuscript.

Field-specific reporting

Please select the one below that is the best fit for your research. If you are not sure, read the appropriate sections before making your selection.

[x] Life sciences [ ] Behavioural & social sciences [ ] Ecological, evolutionary & environmental sciences

For a reference copy of the document with all sections, see nature.com/documents/nr-reporting-summary-flat.pdf

Life sciences study design

All studies must disclose on these points even when the disclosure is negative.

Sample size	For in vitro studies, assays were replicated in triplicate to ensure reproducibility. For in vivo studies, n = 7 mice per group were used to provide sufficient statistical power. For qualitative imaging (SEM, TEM, confocal, and histopathology), one representative biological sample (n = 1) was used per group
Data exclusions	Outliers were identified using Grubbs' test. Data identified as statistical outliers at multiple time points were excluded to preserve the continuity of the healing profile
Replication	In vitro experiments were performed in three independent biological replicates. For qualitative imaging, the consistency of the reported features was verified by capturing and examining at least three different fields of view per biological sample to ensure that the observations were representative of the entire specimen.
Randomization	For in vitro studies, samples were randomly collected. For in vivo studies, mice were randomly allocated.
Blinding	Blinding was not performed during the experiments. Investigator bias was minimized by relying on objective, instrument-based measurements throughout the study: in vitro assays (MIC, hemolysis, time-kill, membrane depolarization) used automated microplate readers; imaging measurements (SEM, TEM, confocal, wound) were quantified using built-in instrumental scale bars or physical reference rulers, and at least three independent fields of view per biological sample were examined to ensure representativeness. Wound area was measured in ImageJ from standardized digital photographs with a reference scale.

Reporting for specific materials, systems and methods

We require information from authors about some types of materials, experimental systems and methods used in many studies. Here, indicate whether each material, system or method listed is relevant to your study. If you are not sure if a list item applies to your research, read the appropriate section before selecting a response.

Materials & experimental systems

n/a	Involved in the study
☒	☐ Antibodies
☐	☒ Eukaryotic cell lines
☒	☐ Palaeontology and archaeology
☐	☒ Animals and other organisms
☒	☐ Clinical data
☒	☐ Dual use research of concern
☒	☐ Plants

Methods

n/a	Involved in the study
☒	☐ ChIP-seq
☒	☐ Flow cytometry
☒	☐ MRI-based neuroimaging

Eukaryotic cell lines

Policy information about cell lines and Sex and Gender in Research

Cell line source(s)	HEK293: Provided by the Facciotti lab (University of California, Davis). Human Red Blood Cells: Purchased from Zen-Bio, Inc. (Research Triangle Park, NC, USA).
Authentication	he HEK293 cell line was not further authenticated after receipt from the source lab. Human red blood cells were used within four days of receipt as per the manufacturer's quality control.
Mycoplasma contamination	The cell lines were not tested for mycoplasma contamination.
Commonly misidentified lines (See ICLAC register)	No cell lines used in this study are listed in the ICLAC database of commonly misidentified cell lines.

Animals and other research organisms

Policy information about studies involving animals; ARRIVE guidelines recommended for reporting animal research, and Sex and Gender in Research

Laboratory animals	All animal experiments were approved by the Institutional Animal Care and Use Committee (IACUC) of the University of California, Davis. Male inbred BALB/c mice (BALB/cAnNCrl) were purchased from Charles River Laboratories (Hollister, CA, USA) at 7–8 weeks of age. Following an acclimation period, mice at 8–9 weeks of age were used for all in vivo studies. Animals were housed in a pathogen-free facility at the University of California, Davis, under a standard 12:12-hour light/dark cycle. The ambient temperature was maintained at 68°F to 79°F with a relative humidity of 30–70%, and all mice were provided ad libitum access to a standard laboratory diet and water.
Wild animals	This study did not involve wild animals
Reporting on sex	We only applied male mice in this animal experiment
Field-collected samples	This study did not involve field-collected samples.
Ethics oversight	All animal experiments were approved by and conducted in accordance with the guidelines of the Institutional Animal Care and Use Committee (IACUC) of the University of California, Davis.

Note that full information on the approval of the study protocol must also be provided in the manuscript.

Plants

Seed stocks	This study did not used seed stocks
Novel plant genotypes	This study did not used novel plant
Authentication	Not applicable
