# Supplementary material for: Discovery of potent low-toxicity antimicrobial peptides through diffusion modeling
Source: Nat Commun. 2026 Jul 7;17:8396. doi: 10.1038/s41467-026-75030-8 (PMC13473622; doi:10.1038/s41467-026-75030-8)
Supplement: Supplementary file 3 [file 41467_2026_75030_MOESM3_ESM.pdf]

nature portfolio

Peer Review File

Discovery of potent low-toxicity antimicrobial peptides through diffusion modeling

Corresponding Author: Professor Ilias Tagkopoulos

This file contains all reviewer reports in order by version, followed by all author rebuttals in order by version.

Version 0:

Reviewer comments:

Reviewer #1

(Remarks to the Author)
Overall Evaluation
This manuscript presents a diffusion-based generative framework for antimicrobial peptide (AMP) discovery, claiming improvements in activity and toxicity prediction through therapeutic-index weighting and iterative learning. While the topic is timely, the work offers only incremental methodological contributions, lacks systematic benchmarking against established generative paradigms, and provides limited guidance on how its iterative strategy generalizes.

1. Limited novelty and lack of systematic evaluation of generative frameworks
The generative component of this work presents limited methodological innovation.
The authors employ the EvoDiff-style discrete diffusion framework (D3PM variant) for unconditional peptide generation, yet the original EvoDiff paper introduced two distinct frameworks—OADM and D3PM—and explicitly reported that OADM generally outperforms D3PM in unconditional generation, while D3PM is more suitable for guidance-based conditioning. The rationale for selecting D3PM instead of OADM is not provided and appears suboptimal: OADM is empirically stronger for unconditional sequence modeling, and no evidence is given that D3PM offers any advantage in this setting.
Moreover, the manuscript omits a systematic comparison with other plausible and widely adopted generative paradigms—such as autoregressive transformers (e.g., GPT-style models), latent-space diffusion, or flow-matching frameworks—that are increasingly standard in biomolecular sequence design.
Without benchmarking across these baselines, it is impossible to evaluate whether D3PM is the most effective or efficient choice.
Finally, the method involves full fine-tuning of the pretrained backbone, which is computationally heavy and risks overfitting to the limited AMP dataset. Lightweight alternatives—such as soft-prompt tuning or adapter-based conditioning that freeze the backbone—could offer more efficient and interpretable optimization. The authors should either explore or justify the exclusion of these established techniques.

2. Limited statistical significance of reported improvements
The headline improvements (e.g., average Log_2MIC 5.81 → 6.07; novelty 0.34 → 0.81) appear to be derived from only 2,000 generated sequences per model variant in Fig. 2a, a small sample relative to the enormous combinatorial search space. Such limited sampling may not reflect the robustness of these results, especially because diversity typically decreases as more sequences are generated.
The paper should therefore disclose the total number of generated sequences, the sampling protocol (temperature, randomness), and report variance across multiple generation runs and random seeds to establish statistical confidence in the claimed gains.

3. Ambiguity and limited generalizability of the active iterative-learning procedure
The manuscript emphasizes an iterative “generate–evaluate–learn” cycle but provides no clear operational guidance. Only two training iterations were actually performed, without any explicit stopping rule and convergence metric.
It remains unclear how the authors determined that training had converged or that additional iterations would not further improve performance.

(Remarks on code availability)
The authors did not provide any source code, repository link, or code availability statement in either the main text or the

Supplementary Information.
Therefore, I was unable to review or test any implementation.
Given that the study relies heavily on custom diffusion and iterative-learning pipelines, the absence of accessible code prevents any assessment of reproducibility or usability for the community.

Reviewer #2

(Remarks to the Author)
In this manuscript, authors made use of AI programs to identify two novel sequences with activity (MIC 8 ug/ml) and 50% hemolytic concentration (LC50 > 512 ug/ml) comparable to pexiganan. These peptides would have high therapeutic index > 64. They then confirmed peptide membrane targeting to produce a pore. Simulation studies implied Arcinin may self-associate, different from melittin. In addition, they found the peptide kept activity in cation-adjusted MHB and 50% serum. Unfortunately, this study is insufficient for Nature Communication. Identification of highly selective peptides is the excitement here but does not garantee in vivo efficacy. Without efficacy and toxicity data in animals, this manuscriot did not reach the mninimal impact level required for a high-impact journal. In addition, the authors mentioned resistance development is the next step. They know what is needed but did not do the work, either. The observation of peptide activity in serum is useful but not a substitute of detailed PK/PD studies in vivo. Without further work, therefore, this manuscript may be transferred to a sister journal.

(Remarks on code availability)

Reviewer #3

(Remarks to the Author)
SUMMARY

Markakis et al describe ARCADIAMP, a generative-discriminative ML framework to discover novel AMPs that are antibacterial and non-toxic. ARCADIAMP applies a D3PM trained on 13,950 curated AMP sequences. The model is augmented through an active learning strategy. 16,334 generated sequences predicted to have MIC < 32 µg/mL are incorporated into the training set and weighted according to an estimated therapeutic index. The authors also trained a two-stage classifier built on ESM2 embeddings. The first is a coarse-grained model distinguishing AMPs from non-AMPs. The second is a fine-grained classifier identifying strong AMPs from weak AMPs. A novelty filter based on global pairwise alignment against 27,636 known AMPs further prioritizes candidates.

From 1,000,000 generated sequences, ARCADIAMP prioritized 76 peptides with high predicted potency and novelty. Ten were selected for synthesis and eight exhibited MIC ≤ 32 µg/mL across at least four ESKAPE species in MHB. The lead peptide, Arcinin (Peptide-7), displayed MIC = 8-16 µg/mL in MHB (except in E. faecalis) and MIC ≤ 32 µg/mL in CaMHB, retained modest activity in 50% bovine serum (MIC =32 µg/mL in four species), and showed low toxicity (RBC LC_{50} > 512 µg/mL and HEK293 LC50 = 50.8 µg/mL). To probe Arcinin's mechanism of action, the authors first applied MD simulations, which show that Arcinin inserts into a DMPC membrane within sub-microsecond timescales. In vitro time-kill curves show bactericidal activity within 60 minutes at 2x-4x MIC in S. aureus, K. pneumoniae, and E. coli. DiSC3(5) assays show rapid depolarization upon Arcinin exposure. SEM and TEM also support a mechanism of action based on membrane disruption.

COMMENTS

1. Given that the paper describes a complex AMP generation framework, much more detail of each component of the architecture is required in the main text. I realize that the SI file contains some additional computational details, but for readers to have a good understanding of each component of the model, it is essential that the main text contains additional information. What is the specific architecture of each module? What was each trained on (specifically)? What was the training scheme? How was each module of ARCADIAMP independently validated? What ablation studies were conducted to show the contribution of each component of ARCADIAMP for non-toxic AMP discovery? These questions do not seem to be accessibly answered in the main text.

2. The color scheme for Figure 1c and Figure 3a needs to be revised to make the differences in colors much more obvious.

3. I am unable to see text references to multiple panels in Figure 2; I can only see Fig 2a, b, d referenced in the text. Given that Figure 2 is the only benchmarking figure showing the performance of ARCADIAMP relative to other AMP discovery methods, this needs far more elaboration in the text. Furthermore, the authors should benchmark their study against this architecture (https://www.science.org/doi/10.1126/sciadv.adp7171), which generated reasonably potent AMPs.

4. Regarding the in vitro potency experiments in Figure 2d and Figure 2f, what are the details of these assays? What bacterium is being assessed? Moreover, the authors emphasize that that advantage of ARCADIAMP is its ability to generate AMPs with low potency. In these benchmarking experiments, the authors should therefore test the AMPs in Figure 2d and Figure 2f for both antibacterial potency and HEK293 toxicity + hemolysis. Are AMPs discovered through benchmarked methods more toxic?

5. There is no mention of Figure 3f in the text.

6. Using bovine serum as a proxy for ADME properties is overly simplistic. This assay shows non-specific AMP binding, which is only one part of highly complex ADME relationships. I remain unconvinced of the ADME claims in the paper. Can the authors clarify whether serum binding (or other ADME properties) was modelled in ARCADIAMP, or whether this was determined entirely post hoc?

7. Based on the data shown in Table 1, it seems that peptide-2 has a more favorable therapeutic window than Arcinin. Why was this peptide not investigated in greater detail?

8. The authors need to provide links to their GitHub repo so code can be tested, and the results can be reproduced. I was unable to find a GitHub link in the main text file or the SI file.

RECOMMENDATION

Modelling toxicity (and ADME properties, if reasonably possible) is essential to develop peptides that overcome many of the limitations of existing AMPs. I think the focus of the paper is really important to advance the AMP field. However, numerous computational and experimental questions remain.

(Remarks on code availability)
I was unable to find a link to the codebase used for this study.

Reviewer #4

(Remarks to the Author)
The manuscript presents the biological characterisation of an Antimicrobial peptide (AMP), which has been discovered using special AI methods.
While the scientific background might be solid, its presentation is quite confusing, lacking significant details or explanations. In addition, the manuscript has quite inconsistencies on which information is provided in the Methods or in the Results.

For example the Result section (line 70-102) is full of jargon, some unexplained details (like 'pretrained model (EvoDiff-D3pm, see SOM)' or 'sample-weights'). Only by reading the Methods section, the reader is somehow able to understand the corresponding Result section. In addition some presentation in Figure 2 are not explained in the manuscript: (panels e and f; what's PEPNET (Seq only) or PEPNET (Structure feature added))

The authors would need to re-write and re-structure the the Dataset and AI section, to make it more comprehensible for non-AI or even AI experts: better descriptions of the datasets (peptide length distribution, secondary structures, biological activity of DBAASP, CAMPR4 dataset and non-AMP (similar sized peptides or proteins), which type of antimicrobial data (single concentration or dose response data) and which toxicity data has been used in the training; performance (if relevant) of the generative and predictive models; conceptual difference between coarse and fine-grained models.
It would also be interesting if the generative and predicative models have any preferences (or bias) for certain properties common for AMP's, like amphiphilicity, number of positive charges, secondary structures (helical or beta-turns,...), based on the train set.

Microbiology: Better introduction of acronyms (like ESKAPE, which organisms they include and why are they important); Why is the difference in activity between MHB and CaMHB and what is its significance in this context; which activity data has been used for the AI training (from MHB or CaMHB); Which labware has been used, especially any surface treatments of micro-titre plates, which affect the in vitro activities of AMP (i.e NBS plate, untreated PS plates or addition of Tween). For the determination of the MIC the language is unclear if visual growth or OD600 (line 279) has been used (and which threshold for percentage growth inhibition, in case of OC600.

The descripted in vitro ADME assay (line 281) is not an ADME assay, but is Growth Inhibition Assay in the presence of Serum (aka serum reversal), a surrogate for Plasma Protein Binding (PPB). What was used as positive control for cytotox or haemolysis (i. Melittin could have been used for HC50).

Figure 3a/b/c better table with corresponding MIC values and better explanation of which (MHB or CaMHB) values have been used for the therapeutic index, and how much 'serum reversal' is observed.

MD simulations: better descriptions of the starting structures of Melittin and the non-AMP (a-helical structure as well?), Some unclear terminologies like 'aqueous conditions' (line 126), or 'central loop between two a-helices' (line 130, both melittin and arcinin have 2 helices with a loop in between), 'thinner DMPC' (line 255; by how much compared to a regular membrane). In addition, very little details on the structural changes (if any) of melittin or arcinin on penetrating the membrane.
The secondary structure of arcinin should have evaluated by Circular Dichroism (or NMR) to validate the predicted helical structure. Also no description if arcinin (or the other 6 peptides) has any amphiphillic nature.

In summary, while the work has some merit (even though marginal potency and some important experiments are missing, such as serum/plasma stability, or microsome stability or even some in vivo validation, the presentation of the work needs better structure, especially for the AI part.

(Remarks on code availability)
No code available

Version 1:

Reviewer comments:

Reviewer #1

(Remarks to the Author)
The revised manuscript has been substantially improved, and the authors have addressed my main concerns from the previous round. I still view the methodological advance as more application-driven than fundamentally algorithmic, and some aspects of the benchmarking could remain more comprehensive. However, in my view, the current revision adequately supports the main claims of the manuscript, and the combination of computational development and experimental validation makes the work suitable for publication.

(Remarks on code availability)
I inspected the repository structure and documentation, but did not fully install or execute the pipeline, so my assessment is limited to code availability, organization, and apparent usability rather than end-to-end reproducibility.

Reviewer #2

(Remarks to the Author)
The manuscript has been revised and strengthened. This reviewer appreciate the new addition that illustrate peptide effect in one animal model with 3 log CFU decrease compared to the control group (Fig. 5). However, problems perist. The animal study does not convey the signifiance of the great peptide claimed in the manuscript. The authors used only a classic wound model without testing any potential therapeutic effects on bloodstream infection since the peptide is stable in serum. Intraveous injection will tell the toxicity of the peptide. In addition, the wound model was not conducted properly. By ignoring splinting in the process of surgery, the self contraction of wound tissues in the murine model cannot be excluded. Indeed, wound closure difference between the negative control (40%) and the treated group (60%) is not impressive. In summary, the animal data is not sufficent to support the claim that the peptidde can work in blood.

In addition, the authors refused to do the standard multiple passage experiment to prove no resistance development. The use of literature for argument is weak and not convincing.

Finally, it is interesting to note that the program has predicted peptide MIC at 0.007 which is much much lower than what they observed at 8 ug/ml. It appears that the prediction is a computer game that can pushes the MIC as low as posisble. However, the predicted low MIC values did not appear in real experiments. It will be really good to see the correlation between the prediction and experiment in future studies.

(Remarks on code availability)

Reviewer #3

(Remarks to the Author)
I appreciate the additional work put into this paper. Many of my questions have been addressed. However, I have some remaining comments that should be addressed.

1. I find the computational aspects of the paper poorly described in the Results section of the paper. I strongly believe that the authors should expand their descriptions of (a) model training schemes [including pre-training and iterative training rounds, as well as their data splitting method]; and (b) more specifically how their final model architecture compared to the two existing AMP design models referenced [BroadAMP-GPT and latent diffusion]. These Results section text and figure panels will be important for readers to understand where ACARDIAMP is preferable to other methods, and where it comparatively lacks performance. Moreover, a more concrete discussion of the novelty of Arcinin relative to known AMPs is warranted. Given that it is a linear membrane-active peptide, which most AMPs are, the authors should more clearly describe the novelty of this AMP. Why is it more promising than existing AMPs that function the same and likely have similar physicochemical properties?

2. The paragraph from line 128-137 is still confusing. Figure 2d and Figure 2f still read as if these are prospective validations. The authors must more throughly explain these computational experiments, as well as the importance of the results in the context for forthcoming prospective AMP identification for in vitro experiments.

3. In their rebuttal, the authors state:

"We used toxicity data to tune the training weights so that the generative model would preferentially produce lower-toxicity peptides. Because the available toxicity data are limited and heterogeneous, for example lacking a universally reported hemolysis percentage at a given peptide concentration across most studies, we did not construct a standalone toxicity

predictor for the pipeline. Instead, to demonstrate that the revised pipeline enriches for lower-toxicity candidates, we compared two experimental batches: candidates from the initial screening pipeline, which used equal sample weights during generative-model training (Supplementary Table 11), and candidates from the final screening pipeline, which used therapeutic-index-based sample-wise weights (Table 1).

Can the authors provide more details about how "equal sample weights" and "therapeutic-index-based sample-wise weights" are constructed? I am unable to find a clear/detailed explanation in the main text or methods.

4. As an editorial note, the Supplementary file has all edits tracked.

(Remarks on code availability)
Overall, the codebase is very clear and the installation/Full pipeline ran smoothly. I appreciate how the authors have made it easy to run the code by including Jupyter notebooks for the full pipeline and each modular step in training and inference. This codebase is of sufficient quality for publication and community use.

Reviewer #4

(Remarks to the Author)
The author implemented most/all of the reviewers suggestions, making the manuscript much more readable and comprehensive. In addition they added efficacy from an in vivo wound model, making the manuscript more acceptable for Nat Comm.

Some remaining issues:
Please provide more details on how the compounds were administered in the wound model, like type (Water or PBS) and volume of solution, concentration of positive controls and AMP in the solution, and any process after administration (i.e drying or waiting for absorption).

(Remarks on code availability)
Most of the code is in jupyter notebook style, with good help text and instruction on how to replicate the training and prediction of the AMPClassifier and Generative models, providing all data and pre-trained models within the github repository. The installation is straight forward.
The repository would benefit from a proper test of a short/small training or prediction step, rather than just a validation of installed torch and evodiff packages.

Open Access This Peer Review File is licensed under a Creative Commons Attribution 4.0 International License, which permits use, sharing, adaptation, distribution and reproduction in any medium or format, as long as you give appropriate credit to the original author(s) and the source, provide a link to the Creative Commons license, and indicate if changes were made.
In cases where reviewers are anonymous, credit should be given to 'Anonymous Referee' and the source.
The images or other third party material in this Peer Review File are included in the article's Creative Commons license, unless indicated otherwise in a credit line to the material. If material is not included in the article's Creative Commons

license and your intended use is not permitted by statutory regulation or exceeds the permitted use, you will need to obtain permission directly from the copyright holder.
To view a copy of this license, visit https://creativecommons.org/licenses/by/4.0/

Reviewers' comments:

Reviewer #1 (Remarks to the Author):

Overall Evaluation

This manuscript presents a diffusion-based generative framework for antimicrobial peptide (AMP) discovery, claiming improvements in activity and toxicity prediction through therapeutic-index weighting and iterative learning. While the topic is timely, the work offers only incremental methodological contributions, lacks systematic benchmarking against established generative paradigms, and provides limited guidance on how its iterative strategy generalizes.

1. Limited novelty and lack of systematic evaluation of generative frameworks

The generative component of this work presents limited methodological innovation.

The authors employ the EvoDiff-style discrete diffusion framework (D3PM variant) for unconditional peptide generation, yet the original EvoDiff paper introduced two distinct frameworks—OADM and D3PM—and explicitly reported that OADM generally outperforms D3PM in unconditional generation, while D3PM is more suitable for guidance-based conditioning.

The rationale for selecting D3PM instead of OADM is not provided and appears suboptimal: OADM is empirically stronger for unconditional sequence modeling, and no evidence is given that D3PM offers any advantage in this setting.

Response:

We compared OADM and D3PM using sequence novelty metrics (similarity and identity relative to the closest well-known AMPs; ***Supplementary Table 6****) together with predicted MIC values generated by the published MIC predictor of Huang et al. (Nat. Biomed. Eng. 7, 797–810 (2023)) on 30,000 generated sequences. Across the full set of 30,000 sequences, OADM performed better overall: sequences generated by OADM had significantly lower predicted MIC values than those generated by D3PM*

*(**Supplementary Figure 5a**), although D3PM produced sequences with slightly better novelty (median similarity: 0.67 vs 0.68; median identity: 0.71 vs 0.74; $p < 0.001$ in both cases; **Supplementary Figure 5b,c**). However, in the practical screening scenario, in which we selected the top 100 candidate sequences with the lowest predicted MIC values, D3PM outperformed OADM by yielding significantly lower predicted MIC values (median predicted MIC: 0.007 vs 0.014; $p < 0.001$; **Supplementary Figure 6a**) together with slightly better similarity (median similarity: 0.954 vs 0.966; $p < 0.001$). Based on these results, we concluded that D3PM was the better choice for the practical screening setting used in this study.*

*We also found that fine-tuning pretrained models, or removing the cross-entropy loss, produced sequences with significantly better novelty but substantially lower predicted antibacterial activity. Because we prioritized antibacterial activity over sequence novelty, we chose to train the model from randomly initialized weights rather than fine-tuning a pretrained model. Sequence novelty can be further improved through iterative learning, and those comparison results are now shown in **Supplementary Figure 10**.*

Moreover, the manuscript omits a systematic comparison with other plausible and widely adopted generative paradigms—such as autoregressive transformers (e.g., GPT-style models), latent-space diffusion, or flow-matching frameworks—that are increasingly standard in biomolecular sequence design. Without benchmarking across these baselines, it is impossible to evaluate whether D3PM is the most effective or efficient choice.

Response:

This variant of the discrete diffusion model offers several advantages over alternative generative architectures. First, it natively supports the generation of sequences with predefined lengths, which is essential for designing peptides within a therapeutically relevant range. Second, it can be repurposed as an inpainting model, allowing specific amino acid residues to be anchored at defined positions while the remaining sequence is generated — a feature that may be valuable for future structure-guided

design. Third, the D3PM framework offers considerable flexibility in shaping the training objective through a parameterizable hybrid loss function, enabling sample-wise weighting of the kind employed in ARCADIAMP.

We compared our model against a GPT-style model, BroadAMP-GPT (Li et al., Gut Microbes 17(1), 2523811), and a latent-space diffusion model, PepDiffusion (Wang et al., Science Advances 11(6), eadp7171). PepDiffusion generated sequences with significantly higher novelty than our approach; however, our model produced sequences with significantly lower predicted MIC values than either comparison model (now shown in ***Supplementary Figure 9****). Because we prioritized antibacterial potency over sequence novelty, we consider our approach to be the most effective among the models evaluated.*

Regarding flow-matching-based models, we were unable to identify a published implementation suitable for rigorous benchmarking. The only accessible example we found, FlowAMP (available at https://huggingface.co/esunAI/FlowAMP), lacks peer-reviewed documentation and sufficient methodological detail to permit a fair and reproducible comparison. We therefore excluded it from the benchmark.

Finally, the method involves full fine-tuning of the pretrained backbone, which is computationally heavy and risks overfitting to the limited AMP dataset. Lightweight alternatives—such as soft-prompt tuning or adapter-based conditioning that freeze the backbone—could offer more efficient and interpretable optimization. The authors should either explore or justify the exclusion of these established techniques.

Response:

Thank you for the comment. We would like to clarify that our approach does not fine-tune a pretrained backbone. We retained only the model architecture and trained it from randomly initialized weights. Although training from a pretrained D3PM model yielded sequences with significantly better novelty — that is, lower similarity and identity relative to the closest well-known AMPs (similarity: 0.67 without pretraining vs. 0.46 with pretraining; identity: 0.71 without pretraining vs. 0.52 with pretraining) — it

also produced sequences with significantly higher predicted MIC values, indicating weaker predicted antibacterial activity (495.3 µM without pretraining vs. 3,390.5 µM with pretraining; ***Supplementary Figure 5****).*

This reflects an inherent trade-off: because the vast majority of naturally occurring peptide sequences are non-antimicrobial, a model pretrained on general protein sequences is not biased toward antibacterial activity. Training exclusively on experimentally validated AMPs from random initialization corrects this bias, but at the cost of lower sequence diversity — the model has fewer examples to learn from and tends to generate sequences that closely resemble its training data. Using a pretrained backbone, or training with the variational lower bound loss term alone, reduces this similarity (i.e., improves novelty) but substantially increases predicted MIC values.

Mitigating this diversity–activity trade-off is precisely the motivation for our iterative learning strategy. In each subsequent training iteration, we incorporate generated sequences that are both sufficiently novel and predicted to have low MICs — that is, candidates that the current model already places in the potent, unexplored region of sequence space. By retraining on these sequences, the model progressively extends its coverage of the sequence space of potent AMPs, improving novelty without sacrificing predicted antibacterial activity. In this way, iterative learning achieves what neither random-initialization training nor pretrained-model fine-tuning can accomplish alone: a generative model that is simultaneously biased toward high antibacterial potency and capable of exploring diverse, previously unseen sequence space.

2. Limited statistical significance of reported improvements
The headline improvements (e.g., average Log□MIC 5.81 → 6.07; novelty 0.34 → 0.81) appear to be derived from only 2,000 generated sequences per model variant in Fig. 2a, a small sample relative to the enormous combinatorial search space.
Such limited sampling may not reflect the robustness of these results,

especially because diversity typically decreases as more sequences are generated.
The paper should therefore disclose the total number of generated sequences, the sampling protocol (temperature, randomness), and report variance across multiple generation runs and random seeds to establish statistical confidence in the claimed gains.

Response:

Thank you for the suggestion. We increased the comparison sample size to at least 30,000 generated sequences and clarified the sampling procedure, particularly sequence length selection. The D3PM model does not use a temperature parameter during generation.

We also quantified variance by generating different numbers of sequences (122, 520, 1019, and 3021; the non-round values reflect our attempt to match the length distribution of the training set) 100 times for each setting. For these 100 replicates, we calculated the 95th and 5th quantiles of the average or median predicted MIC values and defined variance as the relative difference $\left(\frac{95\%\ \boldsymbol{Quantile} - 5\%\ \boldsymbol{Quantile}}{5\%\ \boldsymbol{Quantile}}\right)$ *between those two quantiles. The variance decreased as the number of generated sequences increased and fell below 20% once the number of generated sequences reached 3,000 (**Supplementary Figure 41a,b**).*

*We applied the same analysis to sequence similarity and identity. The variance of these two novelty-related metrics was even smaller (< 5%). We did observe a slight but statistically significant decrease in novelty as the number of generated sequences increased (**Supplementary Figure 41c,d**). Nevertheless, based on experiments with even larger generated sets, novelty does not continue to change substantially once the number of sequences becomes sufficiently large.*

Based on these results, we believe that with a sufficiently large number of generated sequences (> 3,000), the variance of the average or median predicted MIC, similarity, and identity values does not materially affect our conclusions.

3. Ambiguity and limited generalizability of the active iterative-learning procedure

The manuscript emphasizes an iterative “generate–evaluate–learn” cycle but provides no clear operational guidance. Only two training iterations were actually performed, without any explicit stopping rule and convergence metric. It remains unclear how the authors determined that training had converged or that additional iterations would not further improve performance.

Response:

We found that an additional training iteration further improved performance, both in sequence novelty and predicted antibacterial activity, especially when sample-wise weighting was not applied during the first iteration. We therefore performed an additional experiment evaluating one to five training iterations. We observed that predicted MIC values decreased as more iterative-learning rounds with augmented training sequences were applied. Depending on the sample-wise-weighting configuration, the model trained for five iterations produced predicted MIC values that were 33% to 45% lower than those of the model trained for only two iterations which is now shown in ***Supplementary Figure 10a****. Sequence novelty began to deteriorate after the fourth training iteration for the current setting (****Supplementary Figure 10b,c****). In addition, we have added a formal stopping criterion to the Methods section. Training iterations continue until sequence novelty (median similarity to known AMPs) begins to degrade relative to the previous iteration, which empirically occurred after iteration 4 in our experiments (Supplementary Figure 10b,c).*

Reviewer #1 (Remarks on code availability):

The authors did not provide any source code, repository link, or code availability statement in either the main text or the Supplementary Information. Therefore, I was unable to review or test any implementation.

Response:

The repository has now been published and is cited in the revised

manuscript. We apologize for this oversight.

Given that the study relies heavily on custom diffusion and iterative-learning pipelines, the absence of accessible code prevents any assessment of reproducibility or usability for the community.

Reviewer #2 (Remarks to the Author):

In this manuscript, authors made use of AI programs to identify two novel sequences with activity (MIC 8 ug/ml) and 50% hemolytic concentration (LC50 > 512 ug/ml) comparable to pexiganan. These peptides would have high therapeutic index > 64. They then confirmed peptide membrane targeting to produce a pore. Simulation studies implied Arcinin may self-associate, different from melittin. In addition, they found the peptide kept activity in cation-adjusted MHB and 50% serum. Unfortunately, this study is insufficient for Nature Communication. Identification of highly selective peptides is the excitement here but does not garantee in vivo efficacy. Without efficacy and toxicity data in animals, this manuscriot did not reach the mninimal impact level required for a high-impact journal. In addition, the authors mentioned resistance development is the next step. They know what is needed but did not do the work, either. The observation of peptide activity in serum is useful but not a substitute of detailed PK/PD studies in vivo. Without further work, therefore, this manuscript may be transferred to a sister journal.

Response:

We appreciate the reviewer's comment regarding the need for in vivo validation to establish the clinical impact of ARCADIAMP. We fully agree that demonstrating efficacy and safety in a complex physiological environment is essential for a high-impact study. To address this point and further substantiate the therapeutic potential of our lead candidate, ***we performed additional in vivo experiments using a murine full-thickness excisional wound model infected with S. aureus and E. coli, which are now included as Figure 5.***

These results demonstrate that Arcinin is not only potent in vitro but

also highly effective in a living system.

- *Significant in vivo efficacy: Arcinin treatment significantly accelerated wound closure, reaching approximately 60% closure by Day 6 compared with only 40% in the negative-control group ($p < 0.01$).*
- *Robust antibacterial activity: Arcinin reduced the viable bacterial burden in wound tissue by more than three orders of magnitude ($p < 0.001$).*
- *In vivo biocompatibility: Because Arcinin was administered topically, we focused our safety assessment on local biocompatibility at the treatment site. Histopathological analysis (H&E staining) of skin tissue showed no observable tissue damage or necrosis following Arcinin treatment. Instead, Arcinin created a cytological environment conducive to healing by promoting re-epithelialization and reducing inflammatory-cell infiltration relative to the negative control.*

In addition, while serial passage experiments are beyond the scope of the current study, Arcinin's membrane-disruption mechanism — targeting the physical integrity of the lipid bilayer rather than a specific protein target — is broadly recognized to impose a substantially higher genetic barrier to resistance than conventional antibiotics, and we have noted this as a priority for future investigation.

Given our in vivo mouse findings and given our hit rate, we believe that our therapeutic-index-weighted optimization identified a candidate capable of overcoming key physiological hurdles, such as high ionic strength and proteolytic conditions, that often limit the clinical translation of AMPs.

Reviewer #3 (Remarks to the Author):
SUMMARY

Markakis et al describe ARCADIAMP, a generative-discriminative ML framework to discover novel AMPs that are antibacterial and non-toxic. ARCADIAMP applies a D3PM trained on 13,950 curated AMP sequences. The model is augmented through an active learning strategy. 16,334

generated sequences predicted to have MIC < 32 μg/mL are incorporated into the training set and weighted according to an estimated therapeutic index. The authors also trained a two-stage classifier built on ESM2 embeddings. The first is a coarse-grained model distinguishing AMPs from non-AMPs. The second is a fine-grained classifier identifying strong AMPs from weak AMPs. A novelty filter based on global pairwise alignment against 27,636 known AMPs further prioritizes candidates.

From 1,000,000 generated sequences, ARCADIAMP prioritized 76 peptides with high predicted potency and novelty. Ten were selected for synthesis and eight exhibited MIC ≤ 32 μg/mL across at least four ESKAPE species in MHB. The lead peptide, Arcinin (Peptide-7), displayed MIC = 8-16 μg/mL in MHB (except in E. faecalis) and MIC ≤ 32 μg/mL in CaMHB, retained modest activity in 50% bovine serum (MIC =32 μg/mL in four species), and showed low toxicity (RBC LC□□ > 512 μg/mL and HEK293 LC50 = 50.8 μg/mL). To probe Arcinin's mechanism of action, the authors first applied MD simulations, which show that Arcinin inserts into a DMPC membrane within sub-microsecond timescales. In vitro time-kill curves show bactericidal activity within 60 minutes at 2x-4x MIC in S. aureus, K. pneumoniae, and E. coli. DiSC3(5) assays show rapid depolarization upon Arcinin exposure. SEM and TEM also support a mechanism of action based on membrane disruption.

COMMENTS

1. Given that the paper describes a complex AMP generation framework, much more detail of each component of the architecture is required in the main text. I realize that the SI file contains some additional computational details, but for readers to have a good understanding of each component of the model, it is essential that the main text contains additional information. What is the specific architecture of each module? What was each trained on (specifically)? What was the training scheme? How was each module of ARCADIAMP independently validated? What ablation studies were conducted to show the contribution of each component of ARCADIAMP for non-toxic

AMP discovery? These questions do not seem to be accessibly answered in the main text.

Response:

We separated the training procedures into two subsections, one for the generative model and one for the antibacterial activity classifier, and added substantially more detail to the main text on the generative model, including the architecture (D3PM), the training strategy (training from random initialization rather than fine-tuning a pretrained model), and the validation procedures. Specifically, we now describe four complementary comparisons: against the OADM-based model, against a model trained without augmented sequences, against three published models, and against models trained with different numbers of iterative-learning rounds.

By separating these sections and adding clearer training and validation details for both the generative model and the antibacterial activity classifier, we believe the revised main text now more clearly conveys the contribution of each component of ARCADIAMP to the discovery of non-toxic AMPs.

2. The color scheme for Figure 1c and Figure 3a needs to be revised to make the differences in colors much more obvious.

Response:

We changed the color scheme from light green to dark blue to improve contrast and make the differences more visually apparent.

3. I am unable to see text references to multiple panels in Figure 2; I can only see Fig 2a, b, d referenced in the text. Given that Figure 2 is the only benchmarking figure showing the performance of ARCADIAMP relative to other AMP discovery methods, this needs far more elaboration in the text. Furthermore, the authors should benchmark their study against this architecture (https://www.science.org/doi/10.1126/sciadv.adp7171), which generated reasonably potent AMPs.

Response:

*We corrected the text references to the Figure 2 panels in the manuscript. In addition, we incorporated benchmarking against the GPT-style model BroadAMP-GPT (Li et al., Gut Microbes 17(1), 2523811) and the latent-space diffusion model of Wang et al. (Science Advances 11(6), eadp7171), as suggested by the reviewer. We found that PepDiffusion generated sequences with significantly higher novelty than our approach (**Supplementary Figure 9b,c**); however, our model produced sequences with significantly lower predicted MIC values than the three comparison models (**Supplementary Figure 9a**). Because antibacterial activity was our primary design objective, we consider our model the most effective among those compared.*

4. Regarding the in vitro potency experiments in Figure 2d and Figure 2f, what are the details of these assays? What bacterium is being assessed? Moreover, the authors emphasize that that advantage of ARCADIAMP is its ability to generate AMPs with low potency. In these benchmarking experiments, the authors should therefore test the AMPs in Figure 2d and Figure 2f for both antibacterial potency and HEK293 toxicity + hemolysis. Are AMPs discovered through benchmarked methods more toxic?

Response:

***Figure 2** was intended to compare classifier performance against published computational models rather than to present in vitro validation results. The test dataset consists of 195 AMPs targeting E. coli together with 19,143 non-AMPs. We revised the text to make this distinction explicit and avoid further confusion.*

We used toxicity data to tune the training weights so that the generative model would preferentially produce lower-toxicity peptides. Because the available toxicity data are limited and heterogeneous, for example lacking a universally reported hemolysis percentage at a given peptide concentration across most studies, we did not construct a standalone toxicity predictor for

*the pipeline. Instead, to demonstrate that the revised pipeline enriches for lower-toxicity candidates, we compared two experimental batches: candidates from the initial screening pipeline, which used equal sample weights during generative-model training (**Supplementary Table 11**), and candidates from the final screening pipeline, which used therapeutic-index-based sample-wise weights (**Table 1**). The final-screening candidates showed lower toxicity than the candidates from the initial screening.*

5. There is no mention of Figure 3f in the text.

Response:

*We added a description of membrane-hole formation in the revised manuscript and cited the corresponding panel. Because we also added a CD spectrum, the former **Figure 3f** is now labeled **Figure 3g**.*

6. Using bovine serum as a proxy for ADME properties is overly simplistic. This assay shows non-specific AMP binding, which is only one part of highly complex ADME relationships. I remain unconvinced of the ADME claims in the paper. Can the authors clarify whether serum binding (or other ADME properties) was modelled in ARCADIAMP, or whether this was determined entirely post hoc?

Response:

We agree with the reviewer that "ADME" is a broad term encompassing complex systemic processes. Accordingly, we replaced "in vitro ADME" with "serum stability assay" throughout the manuscript so that the terminology more accurately reflects the scope of the experiment.

Regarding the modeling approach, serum binding was not explicitly modeled as an independent input parameter during the ARCADIAMP generative phase. Instead, it was addressed indirectly through our therapeutic-index-weighted training scheme, which prioritizes sequences with high activity and low toxicity, traits that are often associated with clinically viable and stable AMPs. We then used the serum stability assay

as a post hoc validation to test whether the AI-prioritized candidates retained efficacy in a physiological environment.

*Although we agree that ADME is multifaceted, we focused on serum stability because it represents the most immediate physiological hurdle for fast-acting peptides such as Arcinin (**Figure 4a**). In contrast to hepatic metabolism or renal excretion, which operate over longer timescales, serum protein binding and protease degradation occur immediately after administration. Because AMP-mediated killing typically occurs within 30 minutes (**Figure 4a**), retention of activity in serum is a key determinant of whether a peptide can act before systemic clearance. Our in vivo results (**Figure 5**) support this interpretation: Arcinin was able to withstand this initial challenge of high ionic strength and serum-associated factors and consequently achieved substantial bacterial reduction in the murine wound model, where immediate local stability is essential.*

7. Based on the data shown in Table 1, it seems that peptide-2 has a more favorable therapeutic window than Arcinin. Why was this peptide not investigated in greater detail?

Response:

*The decisive factor in selecting Arcinin over Peptide-2 was its superior serum stability, that is, its lower degree of serum reversal, as shown in **Supplementary Figure 14** and summarized in **Figure 3a,b**.*

Although both peptides showed identical MICs (8 µg/mL) against E. coli in standard MHB, their performance diverged markedly in the presence of 50% bovine serum:

- Arcinin (Peptide-7): maintained a potent MIC of 32 µg/mL, corresponding to only a 4-fold increase.
- Peptide-2: showed a pronounced loss of activity, with an MIC of 128 µg/mL, corresponding to a 16-fold increase.

These results indicate that Peptide-2 is substantially more susceptible to inactivation by serum proteins or other physiological factors, which often

causes candidates to fail when moving from in vitro testing to in vivo evaluation. In contrast, Arcinin retained strong activity under serum-rich conditions, suggesting a more favorable therapeutic window under physiologically relevant conditions.

This rationale was ultimately supported by Arcinin's robust efficacy in our murine wound infection model.

8. The authors need to provide links to their GitHub repo so code can be tested, and the results can be reproduced. I was unable to find a GitHub link in the main text file or the SI file.

Response:

The repository has now been published and is cited in the revised manuscript. We apologize for this oversight.

RECOMMENDATION

Modelling toxicity (and ADME properties, if reasonably possible) is essential to develop peptides that overcome many of the limitations of existing AMPs. I think the focus of the paper is really important to advance the AMP field. However, numerous computational and experimental questions remain.

Reviewer #3 (Remarks on code availability):
I was unable to find a link to the codebase used for this study.

Response:

The repository has now been published and is cited in the revised manuscript. We apologize for this oversight.

Reviewer #4 (Remarks to the Author):
The manuscript presents the biological characterisation of an Antimicrobial peptide (AMP), which has been discovered using special AI methods. While the scientific background might be solid, its presentation is quite confusing, lacking significant details or explanations. In addition, the manuscript has quite inconsistencies on which information is provided in the Methods or in the Results.

For example the Result section (line 70-102) is full of jargon, some unexplained details (like 'pretrained model (EvoDiff-D3pm, see SOM)' or 'sample-weights'). Only by reading the Methods section, the reader is somehow able to understand the corresponding Result section. In addition some presentation in Figure 2 are not explained in the manuscript: (panels e and f; what's PEPNET (Seq only) or PEPNET (Structure feature added))

Response:

We substantially revised the first Results section. Specifically, we added an additional paragraph after the opening paragraph to justify the selection of D3PM over OADM, the alternative framework proposed in the original EvoDiff study. That new paragraph also presents the comparison between fine-tuned pretrained models and models trained from randomly initialized weights.

*We also revised the text that refers to **Figure 2** and clarified the description of the classifier comparisons in the Methods section by separating the training of the generative model from the training of the classifiers.*

The authors would need to re-write and re-structure the the Dataset and AI section, to make it more comprehensible for non-AI or even AI experts: better descriptions of the datasets (peptide length distribution, secondary structures, biological activity of DBAASP, CAMPR4 dataset and non-AMP (similar sized peptides or proteins), which type of antimicrobial data (single concentration or dose response data) and which toxicity data has been used in the training; performance (if relevant) of the generative and predictive models; conceptual difference between coarse and fine-grained models.

Response:

*We separated the description of generative-model training from that of classifier training to improve clarity. We also added the length distributions of the training and test sets as well as the secondary-structure distributions of the AMPs in both sets (**Supplementary Figure 32-35**). Because of limitations in data availability and inconsistencies in data formatting, it was not feasible to comprehensively collect biological-activity annotations for all sequences. The revised text now also explains the training procedure more clearly: for the coarse-grained binary classifier, sequences were labeled as positive or negative depending on whether they came from AMP databases or the filtered UniProt set, respectively; for the fine-grained binary classifier, labels were assigned according to MIC values (threshold < 8 µg/ml) against E. coli; and only AMP sequences were used to train the generative model. Toxicity data were used to determine sample weights, but they were not used as direct input features and therefore influenced training only indirectly.*

Finally, the revised sections now introduce the conceptual distinction between the coarse-grained and fine-grained models at the start of the relevant section: "The first classifier (coarse-grained) distinguished AMPs from non-AMPs, while the second (fine-grained) identified strong AMPs (MIC < 8 µg/mL, targeting E. coli)."

It would also be interesting if the generative and predicative models have any preferences (or bias) for certain properties common for AMP's, like amphiphilicity, number of positive charges, secondary structures (helical or beta-turns,...), based on the train set.

Response:

We performed an additional analysis comparing sample-training weights in the generative model, the coarse-grained classifier, and the fine-grained classifier with sequence-level characteristics such as predicted secondary structure, amphiphilicity, and number of positive charges. We observed that

*the models showed preferences with respect to predicted secondary structure. In the generative model, sequences predicted to adopt beta-strand structure tended to receive the highest weights, followed by loop structures or alpha-helices, then irregular structures, whereas sequences with unknown secondary structure received the lowest weights (**Supplementary Figure 38a**). These results suggest that sequences with beta-strand structure may have a higher average therapeutic index than sequences with other predicted structures. We observed a similar preference for beta-strand-containing sequences in both the coarse-grained and fine-grained classifiers (**Supplementary Figure 39a, Supplementary Figure 40a**).*

Microbiology: Better introduction of acronyms (like ESKAPE, which organisms they include and why are they important); Why is the difference in activity between MHB and CaMHB and what is its significance in this context; which activity data has been used for the AI training (from MHB or CaMHB); Which labware has been used, especially any surface treatments of micro-titre plates, which affect the in vitro activities of AMP (i.e NBS plate, untreated PS plates or addition of Tween). For the determination of the MIC the language is unclear if visual growth or OD600 (line 279) has been used (and which threshold for percentage growth inhibition, in case of OC600.

Response:

1. *Introduction of ESKAPE pathogens: We revised the introduction to define the ESKAPE acronym more clearly, namely Enterococcus faecalis, Staphylococcus aureus, Klebsiella pneumoniae, Acinetobacter baumannii, Pseudomonas aeruginosa, and Enterobacter species. These pathogens are recognized by the WHO as high-priority targets because of their ability to "escape" the effects of conventional antibiotics through diverse multidrug-resistance mechanisms. In this study, we used representative strains from this group to validate the broad-spectrum efficacy of our generated candidates.*
2. *MHB versus CaMHB: The difference in activity between Mueller-Hinton*

broth (MHB) and cation-adjusted MHB (CaMHB) is primarily due to the presence of divalent cations (Ca2+ and Mg2+). These ions can stabilize the bacterial outer membrane or compete with cationic AMPs for negatively charged binding sites, such as lipopolysaccharides in Gram-negative bacteria. Testing in CaMHB is therefore highly informative because it better approximates the high ionic strength of physiological fluids. Arcinin's ability to retain activity under these conditions and in the in vivo model indicates robustness against cation-mediated interference.

3. *Labware and surface treatments: All MIC assays were performed in standard untreated polystyrene 96-well microtiter plates (SPL). We deliberately avoided surface-treated plates, such as NBS plates, and avoided adding surfactants such as Tween-20 so that the observed antimicrobial activity would reflect peptide-bacterium interactions rather than stabilization effects or altered peptide-plastic binding.*
4. *Visual versus OD600 MIC determination: We observed that sterile medium without buffer typically exhibits an OD600 of approximately 0.045, whereas the addition of buffers and peptides introduces baseline fluctuations ranging from 0.030 to 0.060 because of minor refractive-index differences and instrumental noise. To account for this empirical variation, we used OD600 ≤ 0.060 as a conservative no-growth cutoff. This ensured that any reported MIC corresponded to absorbance values indistinguishable from the upper bound of sterile background noise and therefore represented complete growth inhibition.*

The descripted in vitro ADME assay (line 281) is not an ADME assay, but is Growth Inhibition Assay in the presence of Serum (aka serum reversal), a surrogate for Plasma Protein Binding (PPB). What was used as positive control for cytotox or haemolysis (i. Melittin could have been used for HC50).

Response:

We replaced the term "in vitro ADME assay" with "serum stability assay" throughout the revised manuscript.

We also clarified the positive controls used in the experimental design.

- *For the cytotoxicity assay in HEK293 cells, we used melittin (5.32 µg/mL) and DMSO (IC50: 971.83 µg/mL) as positive controls to confirm assay sensitivity to cell death.*
- *For the hemolysis assay, we used 1% Triton X-100 as the technical positive control to define 100% lysis.*

Although we used pexiganan as a clinical-grade reference in both in vitro toxicity assays, the technical validity of the hemolysis measurements was established using the Triton X-100 control.

Because melittin showed substantial toxicity in our cytotoxicity assay, and because our candidates showed negligible hemolysis up to 512 µg/mL relative to the 100% lysis reference defined by Triton X-100, we believe the current experimental design adequately supports the reported safety profiles of our AMP candidates.

We updated the Methods section to explicitly state the use of Triton X-100 and melittin as positive controls in the respective assays.

Figure 3a/b/c better table with corresponding MIC values and better explanation of which (MHB or CaMHB) values have been used for the therapeutic index, and how much 'serum reversal' is observed.

Response:

Although Figure 3 was intended to provide a visual comparison of antimicrobial trends and toxicity, we agree that precise numerical values are also important.

*Accordingly, we added **Supplementary Figure 15**, which provides a comprehensive table of MIC values for all candidates across three media: MHB, CaMHB, and 50% bovine serum.*

Regarding therapeutic-index calculation, we would like to clarify our approach:

- *Model training: Because standard-medium data are most abundant in public resources such as DBAASP, the initial generative model and weighting scheme used MHB-based MIC values.*
- *Lead selection: In contrast, the decision to select Arcinin (Peptide 7) for in vivo studies was driven specifically by its performance in 50% bovine serum. As shown in **Supplementary Figure 15**, although Peptide-2 and Arcinin had comparable potency in standard MHB, Arcinin retained significantly greater activity in the presence of serum. Against E. coli in particular, Arcinin maintained a much more favorable therapeutic window under physiologically relevant conditions. In other words, Arcinin showed remarkably low serum reversal: whereas many AMPs lose activity entirely or exhibit >10-fold MIC increases in serum, Arcinin's MIC increased by only twofold to fourfold.*

We believe that this robust serum performance is a major contributor to Arcinin's strong therapeutic efficacy in the murine wound infection model.

MD simulations: better descriptions of the starting structures of Melittin and the non-AMP (a-helical structure as well?), Some unclear terminologies like 'aqueous conditions' (line 126), or 'central loop between two a-helices' (line 130, both melittin and arcinin have 2 helices with a loop in between), 'thinner DMPC' (line 255; by how much compared to a regular membrane). In addition, very little details on the structural changes (if any) of melittin or arcinin on penetrating the membrane.

Response:

We revised the terminology and added clarifying detail throughout this section. For example, we replaced "aqueous conditions" with "100 mM NaCl solution" and specified that 74 DMPC molecules per leaflet constitute a thinner membrane than the standard CHARMM-GUI configuration, which

uses 100 DMPC molecules per leaflet for an 8×8 nm^2 membrane.

*For the phrase "central loop between two α-helices," we performed DSSP-based secondary-structure analysis throughout the simulation. At the beginning of the simulation (t = 0 ns), Arcinin adopted two helical segments connected by a loop. The first helix began to transition into an irregular turn at approximately 450 ns, whereas the second helix began to penetrate the membrane at around 800 ns and completed penetration by 850 ns (**Supplementary Figure 18a**). Melittin, by contrast, underwent a different structural transition during penetration and retained a two-helix architecture connected by a loop throughout the process (**Supplementary Figure 18b**).*

*We also included a negative-control case using enterotoxin. In that simulation, no major structural changes were observed, and the peptide ultimately detached from the membrane (**Supplementary Figure 19**).*

The secondary structure of arcinin should have evaluated by Circular Dichroism (or NMR) to validate the predicted helical structure. Also no description if arcinin (or the other 6 peptides) has any amphiphillic nature.

Response:

*Thank you for the suggestion. We measured the CD spectrum of Arcinin in a bacterial membrane-mimetic environment (SDS micelles) and added the result to the revised figure 3 (**Figure 3e**). The double minima at 208 and 222 nm support the predicted α-helical secondary structure. To address the question of amphipathicity, we also included helical wheel projections for the candidate peptides in **Supplementary Figure 16**. With the exception of Peptide-9, these projections highlight the amphiphilic nature of the generated sequences.*

In summary, while the work has some merit (even though marginal potency and some important experiments are missing, such as serum/plasma stability, or microsome stability or even some in vivo validation, the presentation of the work needs better structure, especially for the AI part.

Response:

Thank you for all the suggestions. Taken together, the revised manuscript addresses all major reviewer concerns through rigorous benchmarking against published generative architectures, experimental validation of Arcinin in a murine wound infection model demonstrating three-log bacterial burden reduction and accelerated wound closure, biophysical characterization via CD spectroscopy and helical wheel analysis, and full code release. All the above establish ARCADIAMP as a complete, reproducible, and translationally grounded platform for therapeutic AMP discovery. We would like to thank all reviewers for their thoughtful suggestions that made this work better and more impactful!

Reviewer #4 (Remarks on code availability):

No code available

Response:

The repository has now been published and is cited in the revised manuscript. We apologize for this oversight.

REVIEWERS' COMMENTS

Reviewer #1 (Remarks to the Author):

The revised manuscript has been substantially improved, and the authors have addressed my main concerns from the previous round. I still view the methodological advance as more application-driven than fundamentally algorithmic, and some aspects of the benchmarking could remain more comprehensive. However, in my view, the current revision adequately supports the main claims of the manuscript, and the combination of computational development and experimental validation makes the work suitable for publication.

Response:
We thank the reviewer for the careful evaluation of the revised manuscript and for recognizing the substantial improvements made in response to the previous review round. We appreciate the reviewer's acknowledgement that the revised manuscript now adequately supports the main claims of the study and that the combination of computational development and experimental validation makes the work suitable for publication.

Reviewer #1 (Remarks on code availability):
I inspected the repository structure and documentation, but did not fully install or execute the pipeline, so my assessment is limited to code availability, organization, and apparent usability rather than end-to-end reproducibility.

Response:
We appreciate the reviewer's inspection of the code repository and documentation, as well as the assessment of its availability, organization, and apparent usability. Although the pipeline was not fully executed during review, we are pleased that the repository structure, documentation, and accompanying notebooks were considered useful for supporting accessibility and reproducibility of the ARCADIAMP pipeline.

We thank the reviewer again for the constructive feedback throughout the review process, which substantially strengthened both the manuscript and the accompanying software resources.

Reviewer #2 (Remarks to the Author):
The manuscript has been revised and strengthened. This reviewer appreciate the new addition that illustrate peptide effect in one animal model with 3 log CFU decrease compared to the control group (Fig. 5). However, problems perist. The animal study does not convey the signifiance of the great peptide claimed in the manuscript. The authors used only a classic wound model without testing any potential therapeutic effects on bloodstream infection since the peptide is stable in serum. Intraveous injection will tell the toxicity of the peptide.

Response:
We acknowledge the significance of systemic toxicity as the therapeutic potential criteria. However, while IV-based models and PK/PD profiling are essential for subsequent clinical development, we believe our current assessment is sufficient for the scope of this discovery-stage study.

First, our machine learning pipeline was specifically trained using cell-level toxicity and serum-supplemented MIC as primary inputs. Therefore, validating efficacy through a localized wound model provides the most direct and rigorous demonstration of the model's predictive accuracy and its ability to identify candidates with a high therapeutic index.

Second, consistent with established discovery protocols, we ensured a foundational safety profile by evaluating hemocompatibility in human red blood cells and cytotoxicity against the HEK293 cell line. These metrics provided the necessary justification for proceeding to in vivo validation without the immediate need for IV-based systemic toxicity profiling.

Finally, the serum stability data was primarily intended to ensure Arcinin's persistence within the wound environment, where serum-derived proteases

in exudates often neutralize peptide activity.

In addition, the wound model was not conducted properly. By ignoring splinting in the process of surgery, the self contraction of wound tissues in the murine model cannot be excluded.

Response:

We emphasize that our choice of the non-splinted excisional model was deliberate and aligns with the established methodology for evaluating antimicrobial efficacy in bacteria-infected wounds. While silicone splinting, introduced by Galiano et al. (2004), is a standard in regenerative research (e.g., scar biology), it is not the convention for AMP research. The field of antimicrobial therapy predominantly relies on non-splinted models where bacterial burden is the primary endpoint.

This is reflected in the prevailing trend of recent Nature Communications studies. For instance, multiple studies evaluating bacteria-infected wounds used non-splinted models to measure both wound area and CFU (Pranantyo et al., 2024; Ye et al., 2024; Chen et al., 2026; Xuan et al., 2025; Zhang et al., 2025) or focused exclusively on CFU reduction (Li et al., 2024; Shehadeh et al., 2026) to support their primary therapeutic claims. This field-specific standard can address two critical factors: (1) silicone-induced biofilm formation, which can act as a significant confounding variable; and (2) the chronic stress associated with the single-cage housing required for splint retention, which is known to alter systemic immune responses.

To provide clearer context for the readers, we have refined the Results section ("Arcinin demonstrates therapeutic efficacy in a murine wound infection model") and rearranged figures order (Fig 5b ↔ Fig 5c) in the manuscript to emphasize antimicrobial efficacy as our principal claim, with wound closure data presented as a supporting functional observation.

Now, our primary claim is based on the 4-log reduction in bacterial burden, which is a definitive, contraction-independent measure of Arcinin's potency. Since all experimental groups were subjected to identical conditions, any contraction-related contribution applies equally across treatment and control arms, ensuring the validity of our between-group comparisons.

Indeed, wound closure difference between the negative control (40%) and the treated group (60%) is not impressive. In summary, the animal data is not sufficent to support the claim that the peptidde can work in blood.

Response:

While the reviewer notes the numerical difference on a specific day, we believe the observed results are more significant when interpreted through the kinetics of recovery in an infected environment.

*First, while the reviewer cites a 40% vs 60% closure difference at a single time point, this comparison may not fully capture the kinetic, cumulative therapeutic benefit. Figure 5c shows that the Arcinin-treated group consistently maintains a superior closure rate across the entire 14-day course, translating to an approximately 2-day reduction in time-to-closure. In clinical practice, particularly for infected soft tissues, a 48-hour reduction in 'open-wound time' is a vital therapeutic achievement [**Pranantyo et al., 2024; Ye et al., 2024**], significantly narrowing the window of vulnerability to secondary environmental infections.*

Second, it is crucial to distinguish the primary mechanism of our candidate. Arcinin is an Antimicrobial Peptide, not an exogenous growth factor. Its primary function is the rapid elimination of pathogens—the fundamental bottleneck in infected wound healing. Our 4-log CFU reduction data (Fig. 5b) provides the principal evidence of this potency in the animal model. The acceleration of wound closure is downstream functional indicators that Arcinin has successfully cleared the pathological barrier, allowing the tissue to transition from a stalled inflammatory state to the proliferative phase of healing.

Finally, the histological evidence in (Fig. 5e) further supports this. The H&E staining at Day 8 shows more advanced re-epithelialization and reduced inflammatory cell infiltration in the Arcinin group compared to the control, confirming that the physical closure we observed is backed by superior tissue-level recovery.

In addition, the authors refused to do the standard multiple passage experiment to prove no resistance development. The use of literature for argument is weak and not convincing.

Response:

We appreciate the reviewer's critical feedback regarding the necessity of empirical data to support resistance-profile claims. We recognize that while the membrane-disrupting mechanism of Arcinin presents a distinct physiological challenge to bacteria, theoretical arguments based on general literature are insufficient to claim absolute resistance immunity for a novel de novo peptide.

To address this concern, we have revised the manuscript to pivot from asserting 'resistance avoidance' to describing a 'high fitness barrier' to resistance. This shift is directly supported by the mechanistic evidence (MD simulations and morphological evidence (SEM/TEM)) and bactericidal kinetics. We believe this revised framing provides a more accurate and balanced representation of the peptide's current developmental stage.

1) Introduction Section

Original: "AMPs primarily disrupt bacterial membranes, a mechanism believed to be less susceptible to resistance development"

Revised: "AMPs primarily disrupt bacterial membranes, a mechanism that offers a high fitness barrier to the rapid evolution of resistance observed with traditional protein-targeting, RNA-targeting antibiotics."

2) Discussion Section

Addition: "While this targeted disruption suggests a robust barrier to resistance, formal experimental evolution studies remain necessary to define the long-term resistance landscape of Arcinin and its clinical potential."

Finally, it is interesting to note that the program has predicted peptide MIC at 0.007 which is much much lower than what they observed at 8 ug/ml. It appears that the prediction is a computer game that can pushes the MIC as low as posisble. However, the predicted low MIC values did not appear in real experiments. It will be really good to see the correlation between the prediction and experiment in future studies.

Response:

We appreciate the reviewer's observation regarding the discrepancy between predicted and experimentally measured MIC values. We would like to clarify that quantitative inconsistency between predicted and experimental MIC values is a known limitation of MIC regression models across AMP prediction studies, particularly because MIC values are highly heterogeneous and depend strongly on assay conditions, bacterial strains, and media composition. Importantly, this limitation was one of the primary motivations for our transition from regression-based screening toward the two-stage cascade classifier architecture used in the final ARCADIAMP pipeline. Rather than relying solely on absolute MIC regression outputs, the final framework employs a coarse-grained AMP/non-AMP classifier followed by a fine-grained strong-AMP classifier, which together act primarily as relative ranking and prioritization tools for candidate enrichment. Despite imperfect quantitative MIC calibration, the platform achieved a high experimental hit rate, with 8 out of 10 screened candidates demonstrating antimicrobial activity and multiple candidates achieving single-digit MIC values experimentally.

Reviewer #3 (Remarks to the Author):
I appreciate the additional work put into this paper. Many of my questions have been addressed. However, I have some remaining comments that should be addressed.

Response:

We thank the reviewer for the careful re-evaluation of our revised manuscript and for recognizing the substantial improvements introduced in the current version. We appreciate the remaining comments and have further clarified several aspects of the computational framework and benchmarking analyses.

1. I find the computational aspects of the paper poorly described in the Results section of the paper. I strongly believe that the authors should expand their descriptions of (a) model training schemes [including pre-training and iterative training rounds, as well as their data splitting method]; and (b) more specifically how their final model architecture compared to the two existing AMP design models referenced [BroadAMP-GPT and latent diffusion]. These Results section text and figure panels will be important for readers to understand where ACARDIAMP is preferable to other methods, and where it comparatively lacks performance. Moreover, a more concrete discussion of the novelty of Arcinin relative to known AMPs is warranted. Given that it is a linear membrane-active peptide, which most AMPs are, the authors should more clearly describe the novelty of this AMP. Why is it more promising than existing AMPs that function the same and likely have similar physicochemical properties?

Response:

We appreciate the reviewer's request for clearer discussion regarding the novelty of Arcinin. We agree that Arcinin shares several physicochemical properties with many membrane-active AMPs, including amphipathicity and α-helical propensity. However, the novelty of Arcinin does not arise from a

fundamentally distinct membrane-targeting mechanism, but rather from the combined optimization of multiple therapeutically desirable properties within a de novo generated sequence. Specifically, Arcinin simultaneously demonstrated strong antimicrobial activity across multiple ESKAPE pathogens, low hemolytic activity, low HEK293 toxicity, retention of activity in 50% serum, and in vivo efficacy in a murine wound infection model. In addition, sequence novelty filtering confirmed limited similarity and identity relative to known AMPs. We have clarified this distinction in the revised manuscript to avoid implying mechanistic novelty, which will require further investigation, while emphasizing that the primary innovation lies in the integrated therapeutic profile and the AI-guided discovery process.

2. The paragraph from line 128-137 is still confusing. Figure 2d and Figure 2f still read as if these are prospective validations. The authors must more throughly explain these computational experiments, as well as the importance of the results in the context for forthcoming prospective AMP identification for in vitro experiments.

Response:

Regarding the benchmarking experiments shown in Figure 2d and Figure 2f, we apologize for the remaining ambiguity. These analyses represent retrospective computational benchmarking experiments rather than prospective experimental validation studies. The “top-50 MICs” refer to the experimentally reported MIC values associated with the highest-ranked candidates selected from the independent test dataset. The purpose of these analyses was to evaluate whether different screening architectures preferentially enrich for stronger AMP candidates prior to synthesis and experimental testing. We have revised the corresponding text to clarify the retrospective nature and interpretation of these experiments (e.g., adding the description of the test set, and replacing the text “screened AMPs” with “identified candidates”).

3. In their rebuttal, the authors state:

"We used toxicity data to tune the training weights so that the generative model would preferentially produce lower-toxicity peptides. Because the available toxicity data are limited and heterogeneous, for example lacking a universally reported hemolysis percentage at a given peptide concentration across most studies, we did not construct a standalone toxicity predictor for the pipeline. Instead, to demonstrate that the revised pipeline enriches for lower-toxicity candidates, we compared two experimental batches: candidates from the initial screening pipeline, which used equal sample weights during generative-model training (Supplementary Table 11), and candidates from the final screening pipeline, which used therapeutic-index-based sample-wise weights (Table 1).

Can the authors provide more details about how "equal sample weights" and "therapeutic-index-based sample-wise weights" are constructed? I am unable to find a clear/detailed explanation in the main text or methods.

Response:

We also appreciate the reviewer's request for clearer explanation of the sample-weighting strategy. We have revised the manuscript to more explicitly distinguish between the two training schemes. During the initial training round, all AMP sequences were assigned equal sample weights. During iterative retraining, therapeutic-index-based sample-wise weights were introduced by combining antimicrobial activity and hemolysis measurements into a normalized weighting function designed to prioritize peptides with both strong antibacterial activity and lower toxicity. We additionally provide clearer references to the corresponding algorithmic description and equations in the Supplementary Information.

4. As an editorial note, the Supplementary file has all edits tracked.

Response:

We thank the reviewer for identifying the tracked edits remaining in the Supplementary Information file. These have been removed in the revised

submission.

Reviewer #3 (Remarks on code availability):
Overall, the codebase is very clear and the installation/Full pipeline ran smoothly. I appreciate how the authors have made it easy to run the code by including Jupyter notebooks for the full pipeline and each modular step in training and inference. This codebase is of sufficient quality for publication and community use.

Response:
We appreciate the reviewer's inspection of the code repository and the positive assessment regarding code availability, organization, documentation quality, and apparent usability. We are pleased that the repository structure and accompanying notebooks improved the accessibility and reproducibility of the ARCADIAMP pipeline for the community.

Reviewer #4 (Remarks to the Author):
The author implemented most/all of the reviewers suggestions, making the manuscript much more readable and comprehensive. In addition they added efficacy from an in vivo wound model, making the manuscript more acceptable for Nat Comm.

Response:

We thank the reviewer for the careful re-evaluation of our manuscript and for recognizing that the revised version has become substantially more comprehensive and accessible following the implemented revisions.

Some remaining issues:
Please provide more details on how the compounds were administered in the wound model, like type (Water or PBS) and volume of solution, concentration of positive controls and AMP in the solution, and any process after administration

(i.e drying or waiting for absorption).

Response:

Specifically, we now clarify the vehicle composition, administration volume, peptide concentration, positive-control concentrations, and post-application handling procedures used during the murine wound infection experiments. Arcinin and control treatments were administered topically in PBS at the indicated concentrations and volumes immediately following infection and at the scheduled treatment intervals described in the revised Methods section. We additionally clarify the handling procedure following administration, including the absorption period and wound dressing conditions where applicable.

Reviewer #4 (Remarks on code availability):
Most of the code is in jupyter notebook style, with good help text and instruction on how to replicate the training and prediction of the AMPClassifier and Generative models, providing all data and pre-trained models within the github repository. The installation is straight forward.
The repository would benefit from a proper test of a short/small training or prediction step, rather than just a validation of installed torch and evodiff packages.

Response:

We thank the reviewer again for the constructive suggestions throughout the review process, which substantially improved the clarity and completeness of the manuscript and accompanying code repository.
